# Potent and Selective Benzothiazole-Based Antimitotics with Improved Water Solubility: Design, Synthesis, and Evaluation as Novel Anticancer Agents

**DOI:** 10.3390/pharmaceutics15061698

**Published:** 2023-06-09

**Authors:** Laura Gallego-Yerga, Valentín Ceña, Rafael Peláez

**Affiliations:** 1 Laboratorio de Química Orgánica y Farmacéutica, Departamento de Ciencias Farmacéuticas, Universidad de Salamanca, Campus Miguel de Unamuno, 37007 Salamanca, Spain; 2 Instituto de Investigación Biomédica de Salamanca (IBSAL), Facultad de Farmacia, Universidad de Salamanca, Campus Miguel de Unamuno, 37007 Salamanca, Spain; 3 Centro de Investigación de Enfermedades Tropicales de la Universidad de Salamanca (CIETUS), Facultad de Farmacia, Universidad de Salamanca, Campus Miguel de Unamuno, 37007 Salamanca, Spain; 4 Centro de Investigación Biomédica en Red Sobre Enfermedades Neurodegenerativas (CIBERNED), Instituto de Salud Carlos III, 28029 Madrid, Spain; valentin.cena@gmail.com; 5 Unidad Asociada Neurodeath, Facultad de Medicina, Universidad de Castilla-La Mancha, 02006 Albacete, Spain

**Keywords:** anticancer drugs, tubulin, colchicine site, antimitotic, solubility, drug resistance, glioblastoma

## Abstract

The design of colchicine site ligands on tubulin has proven to be a successful strategy to develop potent antiproliferative drugs against cancer cells. However, the structural requirements of the binding site endow the ligands with low aqueous solubility. In this work, the benzothiazole scaffold is used to design, synthesize, and evaluate a new family of colchicine site ligands exhibiting high water solubility. The compounds exerted antiproliferative activity against several human cancer cell lines, due to tubulin polymerization inhibition, showing high selectivity toward cancer cells in comparison with non-tumoral HEK-293 cells, as evidenced by MTT and LDH assays. The most potent derivatives, containing a pyridine moiety and ethylurea or formamide functionalities, displayed IC_50_ values in the nanomolar range even in the difficult-to-treat glioblastoma cells. Flow cytometry experiments on HeLa, MCF7, and U87MG cells showed that they arrest the cell cycle at the G2/M phases at an early time point (24 h), followed by apoptotic cell death 72 h after the treatment. Tubulin binding was confirmed by microtubule network disruption observed via confocal microscopy. Docking studies support favorable interaction of the synthesized ligands at the colchicine binding site. These results validate the proposed strategy to develop potent anticancer colchicine ligands with improved water solubility.

## 1. Introduction

Cancer is one of the main causes of death worldwide, responsible for approximately one of every six deaths and accounting for nearly ten million deaths in 2020 [1]. Cancer is defined as a group of diseases characterized by the uncontrolled division of abnormal cells beyond their usual boundaries as a consequence of failures of the systems of regulation of cell proliferation and homeostasis. Although cancer research in the past two decades has allowed us to better understand the cellular mechanisms involved in the development of the disease, and many new targets for cancer treatments were identified, the difficulties in achieving high anticancer potency and no side effects together with good pharmacokinetic profiles make the development of effective chemotherapy one of the biggest challenges of pharmaceutical sciences [2].

**Figure 1:**
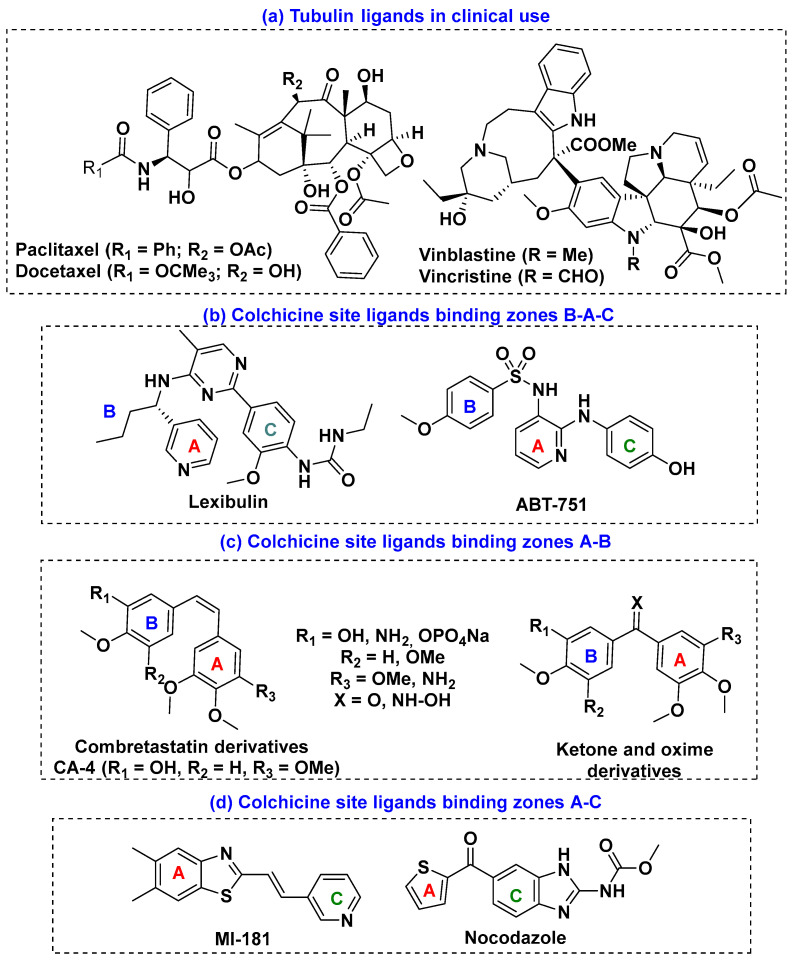
Structure of ligands binding different sites of tubulin.

A considerable number of anticancer drug targets are cell cycle related since it is one of the sources of cell proliferation deregulation. The cell cycle consists of a series of coordinated events, aimed at the formation of two cells from one mother cell, which is usually deregulated in cancer tissues following unscheduled cell divisions. It is composed of four main phases, including growth phase 1 (G1/G0), the DNA synthesis phase (S), growth phase 2 (G2), and mitosis (M), which is the most interesting phase for therapeutic targeting against aberrant cell proliferation since it is the event when cell division takes place. Antimitotic drugs are those that are able to perturb or inhibit cell proliferation by targeting any kind of mitosis machinery such as mitotic kinases, (e.g., the Aurora kinase B inhibitor Barasertib) [3], mitotic phosphatases (e.g., the phosphatase Cdc25 inhibitor NSC95397) [4], kinesins (e.g., the kinesin KSP/EG5 inhibitor Ispenesib) [5], microtubule-associated proteins, MAPs (e.g., the Map2 inhibitor Estracyt) [6], or tubulin (e.g., the microtubule stabilizing agent Paclitaxel) [7]. The polymerization–depolymerization equilibria of tubulin dimers are responsible for the assembly and disassembly of microtubules, which are the main components of the mitotic spindle. Tubulin-binding drugs alter microtubule dynamics, leading to mitotic arrest, which is usually followed by the induction of cell apoptosis. Targeting tubulin has proven to be one of the most successful strategies to develop antimitotic agents since many anticancer drugs in clinical use, such as taxanes [8] (e.g., paclitaxel and docetaxel, Figure 1a) or Vinca alkaloids [9] (e.g., Vinblastine and Vinorelbine, Figure 1a) are tubulin ligands. However, these large and hydrophobic molecules are substrates of efflux pumps such as the P-glycoprotein, thus developing multidrug resistance mechanisms (MDR) [10]. The structural complexity of those natural products entails several difficulties to design and synthesize new analogs to overcome drug resistance mechanisms. 

Different from the taxanes and Vinca alkaloid sites in tubulin, the colchicine site, located at the interface between the α and β tubulin subunits, is appropriate for the design of ligands because it has synthetically accessible simple structures [11,12,13]. In recent years, the colchicine site ligands have gained attention since they behave not only as antiproliferative drugs with a high potency against cancer cells, but also as vascular disrupting agents [14]. According to structural studies, the colchicine site is composed of three connected sub-pockets, namely, B–A–C [15,16,17]. Few examples of ligands binding simultaneously to the three zones were reported, such as Lexibulin [18] and ABT-751 [19] (Figure 1b). The most frequently reported colchicine ligands consist of two aromatic rings linked by different functional groups that bind to two of the colchicine zones, usually zones A–B, such as combretastatin A-4 (CA-4) and its derivatives [20] (Figure 1c), known as classical ligands, which were intensively studied due to their high antimitotic potency [21,22,23]. However, some concerns are involved in the clinical limitations of CA-4 derivatives such as their low water solubility and their susceptibility to phase II metabolic transformations by UDP-glycosyltransferases through the hydroxy groups, which is usually followed by the development of drug resistance [24]. Structural modifications of combretastatin analogs aimed to improve water solubility were attempted by the functionalization of the aromatic rings with more polar groups, but this strategy is usually accompanied by a loss of antiproliferative potency. For example, substitutions of the olefin bridge of combretastatins by more water-soluble linkers, such as ketones or oximes (Figure 1c), did not succeed in maintaining high antiproliferative potencies [25]. 

The incorporation of heterocyclic moieties in the design of new colchicine site ligands was reported as a successful alternative to combretastatin A-4 derivatives [26]. Furthermore, the benzothiazole moiety is a versatile scaffold for the development of new anticancer drugs [27,28] that are able to act on different targets [29]. It has played an important role in the design of new polar compounds targeting tubulin [30], as reported 2-anilinopyridinyl-benzothiazole [31], triazole-benzothiazole [32], naphthalimide-benzothiazole [33], isoxazole-benzothiazole [34], trimethoxyphenyl-benzothiazole [35], and bis-benzothiazole [36] derivatives; most of them are adapted to bind at the A–B zones of the colchicine site. The benzothiazole scaffold was also used to develop photoswitchable tubulin polymerization inhibitors that are able to spatiotemporally control the microtubule dynamics and target different regions of the colchicine domain depending on a photoisomerization process of styrylbenzothiazole derivatives [37]. 

Another approach to obtain more soluble and potent tubulin ligands is the design of molecules targeting the colchicine zones A–C since their amino acid residues are more polar compared with zones A–B [38]. The success of this strategy can be illustrated by the antimitotic agents nocodazole [39] and MI-181 (Figure 1d) [40], a benzothiazole derivative.

MI-181 is a potent antitumor agent acting as an inhibitor of tubulin polymerization. It can inhibit the cell proliferation of HeLa cells with an IC_50_ value of 17 nM. Even though it is more soluble than combretastatin A4 (5.6 µg/mL vs. 1 µg/mL for CA-4), its water solubility is still limited [41]. However, its interaction in the tubulin site was investigated, and its simple structure offers great opportunities to perform chemical modifications aimed to improve its physicochemical and pharmacological properties. 

**Figure 2:**
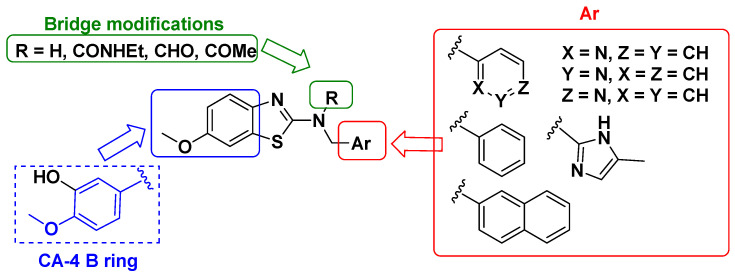
Design of new benzothiazole derivatives reported in this work.

The main objective of this work is the design, synthesis, and biological evaluation of new colchicine site ligands with an improved aqueous solubility, together with a high binding affinity to tubulin, that trigger an antimitotic effect on cancer cells. With this aim, the olefin linker of combretastatins and MI-181 was successfully substituted by a methylamino bridge. As a combination of structural features of the benzothiazole ring of MI-181 and the *p*-methoxyphenyl moiety of Combretastatin A-4, we propose 6-methoxy-benzothiazole as the B ring. We further explored the incorporation of the following different aromatic A rings: 2-, 3-, or 4-pyridine; benzene; 5-metyl-1*H*-imidazole; and naphthalene. Finally, substitutions on the methylamino group to obtain the corresponding formamide, acetamide, or ethylurea derivatives were undertaken (Figure 2). Some of the newly synthesized compounds are more water-soluble than the references MI-181 and ABT-751. Their antiproliferative activity was studied in a selection of human tumor cell lines representative of the kinds of cancer that have the highest incidence worldwide, including cervix uterine, breast, colon, liver, prostate, and brain cancer. Embryonic kidney HEK-293 cells were used to study toxicity in healthy tissues. Multidrug resistance and the mechanism of action were investigated for the most potent compounds. Docking studies were performed for all the new molecules.

## 2. Materials and Methods

### 2.1. Chemistry

#### 2.1.1. General Chemical Techniques

Reagents were used as purchased without further purification. Solvents (toluene, ethanol, EtOAc, CH_2_Cl_2_, MeOH) were dried and stored over molecular sieves. TLC was performed on precoated silica gel polyester plates (0.25 mm thickness) with a UV fluorescence indicator 254 (Polychrom SI F254). Chromatographic separations were performed on silica gel columns via flash (Kieselgel 40, 0.040–0.063; Merck, Rahway, NJ, USA) chromatography. ^1^H NMR and ^13^C NMR spectra were recorded in CDCl_3_, CD_3_OD, DMSO-d_6_, or acetone-d_6_ on a Bruker SY spectrometer at 400/100 MHz or on a Varian Mercury 400/100 MHz spectrometer. Chemical shifts (δ) are given in ppm downfield from tetramethylsilane, and coupling constants (J values) are given in Hz. IR spectra in KBr disks were run on a Nicolet Impact 410 Spectrophotometer. A hybrid QSTAR XL quadrupole/time of flight spectrometer was used for HRMS analyses. The aqueous solubility of the compounds was determined in a Helios Alfa Spectrophotometer from Thermo-Spectronic.

#### 2.1.2. Chemical Synthesis and Characterization

##### Method A. Reductive Amination (Compounds **1**–**6**)

A catalytic amount of *p*-toluenesulfonic acid (0.1 eq) was added to a solution of 6-methoxybenzo[d]thiazol-2-amine (1 eq) and the aromatic aldehyde derivative (1 eq) in dry toluene (30 mL). A Dean–Stark system was used to remove the water, and the mixture was stirred for 16 h under reflux. Then, the solvent was evaporated under reduced pressure, and the residue was re-dissolved in absolute ethanol (20 mL). NaBH_4_ (1.5 eq) was added at 0 °C, and the mixture was stirred at room temperature for 2 h. Then, the solvent was removed under vacuum, and the residue was dissolved in CH_2_Cl_2_. The organic layer was washed with saturated NaCl water, dried over anhydrous Na_2_SO_4_, filtered, and evaporated under vacuum.

##### Method B. Synthesis of Urea Derivatives (Compounds **7**–**12**)

Ethylisothiocyanate (2.5 eq) and triethylamine (2.5 eq) were added to a solution of the 6-methoxybenzo[d]thiazol-2-amine derivative **1**, **2**, **3**, **4**, **5**, or **6** in dry CH_2_Cl_2_ (12–13 mL). The solution was stirred overnight, at room temperature, under N_2_ atmosphere. Then, the solvent was removed under vacuum, and the residue was dissolved in CH_2_Cl_2_. The organic layer was washed with saturated NaHCO_3_ water solution and saturated NaCl water solution, dried over anhydrous Na_2_SO_4_, filtered, and evaporated under vacuum.

##### Method C. Synthesis of Formamide Derivatives (Compounds **13**–**18**)

Formic acid (2 eq), EDC (2 eq), and HOBt (2 eq) were added to a solution containing DMAP (2 eq), and the 6-methoxybenzo[d]thiazol-2-amine derivative **1**, **2**, **3**, **4**, **5**, or **6** in dry CH_2_Cl_2_ (20–22 mL). The mixture was stirred overnight, at room temperature, under a N_2_ atmosphere. Then, the solvent was removed under vacuum, and the residue was dissolved in CH_2_Cl_2_. The organic layer was washed with saturated NaHCO_3_ water solution and saturated NaCl water solution, dried over anhydrous Na_2_SO_4_, filtered, and evaporated under vacuum.

##### Method D. Synthesis of Acetamide Derivatives (Compounds **19**–**24**)

Acetic anhydride (3 eq) and DMAP (3 eq) were added to a solution of the 6-metho-xybenzo[d]thiazol-2-amine derivative **1**, **2**, **3**, **4**, **5** or **6** in dry CH_2_Cl_2_ (12–13 mL). The mixture was stirred for 4 h, at room temperature, under N_2_. Then, the solvent was removed under vacuum, and the residue was dissolved in CH_2_Cl_2_. The organic layer was washed with saturated NaHCO_3_ water solution and saturated NaCl water solution, dried over anhydrous Na_2_SO_4_, filtered, and evaporated under vacuum.

6-methoxy-*N*-(pyridin-2-ylmethyl)benzo[d]thiazol-2-amine (**1**). Compound **1** was synthesized following method A as previously described using commercial 6-methoxybenzo[d]thiazol-2-amine (2 g, 11 mmol) and pyridine-2-carbaldehyde (1.1 mL, 11 mmol) in dry toluene (30 mL) and NaBH_4_ (624 mg, 16.5 mmol) in ethanol (20 mL). The crude residue was purified via flash column chromatography (EtOAc/hexane 3:2–DCM/MeOH 9:1) to yield 2.09 g (7.70 mmol, 70%) of compound **1**. IR (KBr): 3239, 2899, 1623, 1471, 1421, 1335, 1219, 1030, 834 cm^−1^. ^1^H-NMR (400 MHz, MeOD): δ 3.77 (3H, s), 4.72 (2H, s), 6.84 (1H, dd, J = 2.8 and 8.0 Hz), 7.18 (1H, d, J = 2.8 Hz), 7.30 (2H, m), 7.47 (1H, d, J = 7.6 Hz), 7.86 (1H, td, J = 2.0 and 8.0 Hz), 8.50 (1H, bd, J = 4.8 Hz). ^13^C-NMR (100 MHz, CDCl_3_): δ 49.3 (CH_2_), 55.9 (CH_3_), 105.4 (CH), 113.4 (CH), 119.2 (CH), 121.9 (CH), 122.5 (CH), 131.5 (C), 136.8 (CH), 146.3 (C), 148.9 (CH), 155.2 (C), 156.1 (C), 165.3 (C). HRMS (C_14_H_13_N_3_OS) *m*/*z*: calculated 272.0852 (M + H^+^), 294.0672 (M + Na^+^); found 272.0746 (M + H^+^), 294.0661 (M + Na^+^).

6-methoxy-*N*-(pyridin-3-ylmethyl)benzo[d]thiazol-2-amine (**2**). Compound **2** was synthesized following method A as previously described using commercial 6-metho-xybenzo[d]thiazol-2-amine (2 g, 11 mmol) and pyridine-3-carbaldehyde (1.03 mL, 11 mmol) in dry toluene (30 mL) and NaBH_4_ (624 mg, 16.5 mmol) in ethanol (20 mL). The crude residue was purified via flash column chromatography (EtOAc/hexane 3:2–DCM/MeOH 9:1) to yield 2.24 g (8.25 mmol, 75%) of compound **2**. IR (KBr): 3194, 2904, 1607, 1157, 1473, 1222, 1024, 815 cm^−1^. ^1^H-NMR (400 MHz, MeOD): δ 3.76 (3H, s), 4.64 (2H, s), 6.85 (1H, dd, J = 2.8 and 8.8 Hz), 7.17 (1H, d, J = 2.0 Hz), 7.31 (1H, d, J = 8.8 Hz), 7.39 (1H, dd, J = 6.0 and 8.0 Hz), 7.87 (1H, bd, J = 8.0 Hz), 8.42 (1H, dd, J = 6.0 and 1.6 Hz), 8.57 (1H, bs). ^13^C-NMR (100 MHz, CDCl_3_): δ 46.4 (CH_2_), 55.9 (CH_3_), 105.4 (CH), 113.6 (CH), 119.2 (CH), 123.7 (CH), 131.4 (C), 133.6 (C), 135.6 (CH), 146.1 (C), 148.9 (CH), 149.1 (CH), 155.2 (C), 165.5 (C). HRMS (C_14_H_13_N_3_OS) *m*/*z*: calculated 272.0852 (M + H^+^), 294.0672 (M + Na^+^); found 272.0834 (M + H^+^), 294.0664 (M + Na^+^).

6-methoxy-*N*-(pyridin-4-ylmethyl)benzo[d]thiazol-2-amine (**3**). Compound **3** was synthesized following method A as previously described using commercial 6-metho-xybenzo[d]thiazol-2-amine (2 g, 11 mmol) and pyridine-4-carbaldehyde (1.04 mL, 11 mmol) in dry toluene (30 mL) and NaBH_4_ (624 mg, 16.5 mmol) in ethanol (20 mL). The crude residue was purified by flash column chromatography (EtOAc/hexane 3:2–DCM/MeOH 15:1) to yield 2.12 g (7.81 mmol, 71%) of compound **3**. IR (KBr): 3179, 2902, 1624, 1600, 1469, 1415, 1272, 1215, 1060, 801 cm^−1^. ^1^H-NMR (400 MHz, MeOD): δ 3.76 (3H, s), 4.67 (2H, s), 6.84 (1H, dd, J = 2.8 and 8.8 Hz), 7.18 (1H, d, J = 2.8 Hz), 7.28 (1H, d, J = 8.8 Hz), 7.41 (2H, bd, J = 5.6 Hz), 8.45 (2H, bd, J = 4.4 Hz). ^13^C-NMR (100 MHz, CDCl_3_): δ 47.7 (CH_2_), 55.9 (CH_3_), 105.4 (CH), 113.6 (CH), 119.2 (CH), 122.2 (2CH), 131.4 (C), 146.2 (C), 147.5 (C), 149.9 (2CH), 155.2 (C), 165.7 (C). HRMS (C_14_H_13_N_3_OS) *m*/*z*: calculated 272.0852 (M + H^+^), 294.0672 (M + Na^+^); found 272.0765 (M + H^+^), 294.0667 (M + Na^+^).

6-methoxy-*N*-((4-methyl-1*H*-imidazol-5-yl)methyl)benzo[d]thiazol-2-amine (**4**). Compound **4** was synthesized following method A as previously described using commercial 6-methoxybenzo[d]thiazol-2-amine (2 g, 11 mmol) and 4-methyl-1*H*-imidazole-5-carbaldehyde (1.21 g, 11 mmol) in dry toluene (30 mL) and NaBH_4_ (624 mg, 16.5 mmol) in ethanol (20 mL). The crude residue was purified via flash column chromatography (DCM/MeOH 30:1 to 9:1) to yield 2.08 g (7.59 mmol, 69%) of compound **4**. IR (KBr): 3435, 3137, 2932, 1150, 1433, 1223, 1049, 812 cm^−1^. ^1^H-NMR (400 MHz, DMSO-d_6_): δ 2.15 (3H, s), 3.65 (3H, s), 4.33 (2H, d, J = 4.4 Hz), 6.77 (1H, dd, J = 2.8 and 8.8 Hz), 7.23 (1H, d, J = 2.8 Hz), 7.26 (1H, d, J = 8.8 Hz), 7.43 (1H, s), 7.93 (1H, bt, J = 4.4 Hz). ^13^C-NMR (100 MHz, DMSO-d_6_): δ 10.45 (CH_3_), 38.1 (CH_2_), 55.9 (CH_3_), 105.9 (CH), 113.3 (CH), 118.7 (CH), 131.8 (C), 132.5 (C), 133.8 (CH), 134.1 (C), 146.9 (C),154.7 (C), 164.7 (C). HRMS (C_13_H_14_N_4_OS) *m*/*z*: calculated 275.0961 (M + H^+^), 297.0781 (M + Na^+^); found 275.0827 (M + H^+^), 297.0785 (M + Na^+^).

*N*-benzyl-6-methoxybenzo[d]thiazol-2-amine (**5**). Compound **5** was synthesized following method A as previously described using commercial 6-methoxybenzo[d]thiazol-2-amine (1.7 g, 9.43 mmol) and benzaldehyde (0.677 mL, 9.43 mmol) in dry toluene (30 mL) and NaBH_4_ (535 mg, 14.1 mmol) in ethanol (20 mL). The crude residue was purified via flash column chromatography (EtOAc/hexane 1:1–DCM/MeOH 20:1) to yield 1.89 g (6.97 mmol, 74%) of compound **5**. IR (KBr): 3091, 1612, 1442, 1221, 1053, 840 cm^−1^. ^1^H-NMR (400 MHz, CDCl_3_): δ 3.78 (3H, s), 4.58 (2H, s), 6.85 (1H, dd, J = 2.8 and 8.6 Hz), 7.09 (bd, J = 2.4 Hz), 7.38–7.23 (7H, m). ^13^C-NMR (100 MHz, CDCl_3_): δ 45.9 (CH_2_), 55.8 (CH_3_), 105.2 (CH), 113.4 (CH), 119.1 (CH), 127.6 (2CH), 127.7 (2CH), 128.8 (CH), 131.3 (C), 137.6 (C), 146.4 (C), 155.2 (C), 166.0 (C). HRMS (C_15_H_14_N_2_OS) *m*/*z*: calculated 271.0900 (M + H^+^); found 271.0901 (M + H^+^).

6-methoxy-*N*-(naphthalen-2-ylmethyl)benzo[d]thiazol-2-amine (**6**). Compound **6** was synthesized following method A as previously described using commercial 6-metho-xybenzo[d]thiazol-2-amine (1.9 g, 10.54 mmol) and naphthalene-2-carbaldehyde (1.65 g, 10.54 mmol) in dry toluene (30 mL) and NaBH_4_ (598 mg, 15.81 mmol) in ethanol (20 mL). The crude residue was purified via flash column chromatography (EtOAc/hexane 1:2–DCM/MeOH 20:1) to yield 2.26 g (7.065 mmol, 67%) of compound **6**. IR (KBr): 3435, 3196, 1613, 1454, 1219, 1052, 822 cm^−1^. ^1^H-NMR (400 MHz, DMSO-d_6_): 3.59 (3H, s), 4.70 (2H, s), 6.79 (1H, dd, J = 2.4 and 8.8 Hz), 7.21–7.25 (2H, m), 7.41–7.51 (3H, m), 7.81–7.87 (4H, m), 8.53 (1H, bt, J = 7.8 Hz). ^13^C-NMR (100 MHz, DMSO-d_6_): 47.8 (CH_2_), 55.8 (CH_3_), 105.9 (CH), 113.4 (CH), 118.6 (CH), 125.9 (CH), 126.1 (CH), 126.3 (CH), 126.6 (CH), 127.9 (2CH), 128.4 (CH), 131.8 (C), 132.7 (C), 133.3 (C), 137.0 (C), 146.8 (C), 154.8 (C), 165.3 (C). HRMS (C_19_H_16_N_2_OS) *m*/*z*: calculated 321.1056 (M + H^+^); found 321.1055 (M + H^+^).

3-ethyl-1-(6-methoxybenzo[d]thiazol-2-yl)-1-(pyridin-2-ylmethyl)urea (**7**). Compound **7** was synthesized following method B as previously described using compound **1** (250 mg, 0.921 mmol), ethylisothiocyanate (182 µL, 2.30 mmol), and triethylamine (320 µL, 2.30 mmol) in CH_2_Cl_2_ (12 mL). The crude residue was purified via flash column chromatography (EtOAc/hexane 1:3–DCM/MeOH 9:1) to yield 299 mg (0.874 mmol, 95%) of compound **7**. IR (KBr): 3422, 3246, 1667, 1508, 1269, 1061, 802 cm^−1^. ^1^H-NMR (400 MHz, CDCl_3_): δ 1.27 (3H, t, J = 7.2 Hz), 3.44 (2H, bq, J = 7.2 Hz), 3.83 (3H, s), 5.38 (2H, s), 6.98 (1H, dd, J = 2.8 and 8.8 Hz), 7.19 (1H, d, J = 2.4 Hz), 7.24 (1H, bt, J = 5.2 Hz), 7.63–7.70 (3H, m), 8.53 (dd, J = 4.4 and 1.2 Hz), 8.82 (1H, bs). ^13^C-NMR (100 MHz, CDCl_3_): δ 15.0 (CH_3_), 35.2 (CH_2_), 52.3 (CH_2_), 55.8 (CH_3_), 104.0 (CH), 114.4 (CH), 121.1 (CH), 123.0 (CH), 123.7 (CH), 133.8 (C), 137.3 (CH), 143.6 (C), 148.6 (CH), 155.7 (C), 156.2 (C), 156.5 (C), 160.9 (C). HRMS (C_17_H_18_N_4_O_2_S) *m*/*z*: calculated 343.1223 (M + H^+^), 365.1043 (M + Na^+^); found 343.1223 (M + H^+^), 365.1037 (M + Na^+^).

3-ethyl-1-(6-methoxybenzo[d]thiazol-3-yl)-1-(pyridin-3-ylmethyl)urea (**8**). Compound **8** was synthesized following method B as previously described using compound **2** (265 mg, 0.976 mmol), ethylisothiocyanate (193 µL, 2.44 mmol), and triethylamine (340 µL, 2.44 mmol) in CH_2_Cl_2_ (12 mL). The crude residue was purified via flash column chromatography (EtOAc/hexane 1:3–DCM/MeOH 9:1) to yield 301 mg (0.888 mmol, 91%) of compound **8**. IR (KBr): 3435, 3235, 1686, 1557, 1474, 1355, 1026, 835 cm^−1^. ^1^H-NMR (400 MHz, CDCl_3_): δ 1.29 (3H, t, J = 7.6 Hz), 3.48 (2H, bq, J = 7.6 Hz), 3.81 (3H, s), 5.20 (2H, s), 6.98 (1H, dd, J = 2.4 and 8.8 Hz), 7.15 (1H, d, J = 2.4 Hz), 7.25–7.22 (1H, m), 7.60 (1H, d, J = 9.2 Hz), 7.68 (1H, bd, J = 8.4 Hz), 8.51 (1H, bd, J = 4.4 Hz), 8.65 (1H, bs). ^13^C-NMR (100 MHz, CDCl_3_): δ 15.0 (CH_3_), 35.8 (CH_2_), 48.9 (CH_2_), 55.8 (CH_3_), 104.2 (CH), 114.9 (CH), 121.2 (CH), 123.6 (CH), 131.7 (C), 132.4 (C), 134.9 (CH), 149.0 (2CH), 154.1 (C), 156.2 (C), 156.5 (C), 162.2 (C). HRMS (C_17_H_18_N_4_O_2_S) *m*/*z*: calculated 343.1223 (M + H^+^), 365.1043 (M + Na^+^); found 343.1216 (M + H^+^), 365.1035 (M + Na^+^).

3-ethyl-1-(6-methoxybenzo[d]thiazol-4-yl)-1-(pyridin-3-ylmethyl)urea (**9**). Compound **9** was synthesized following method B as previously described using compound **3** (255 mg, 0.939 mmol), ethylisothiocyanate (186 µL, 2.35 mmol), and triethylamine (327 µL, 2.35 mmol) in CH_2_Cl_2_ (12 mL). The crude residue was purified via flash column chromatography (EtOAc/hexane 1:3–DCM/MeOH 9:1) to yield 312 mg (0.911 mmol, 97%) of compound **9**. IR (KBr): 3213, 2970, 1690, 1531, 1469, 1222, 1064, 807 cm^−1^. ^1^H-NMR (400 MHz, CDCl_3_): δ 1.30 (3H, t, J = 7.6 Hz), 3.49 (2H, bq, J = 7.6 Hz), 3.82 (3H, s), 5.20 (2H, s), 6.99 (1H, dd, J = 2.4 and 8.8 Hz), 7.14 (1H, d, J = 2.4 Hz), 7.22 (2H, bd, J = 5.6 Hz), 7.62 (1H, d, J = 8.8 Hz), 8.54 (2H, bd, J = 6.4 Hz), 9.65 (1H, bs). ^13^C-NMR (100 MHz, CDCl_3_): δ 15.0 (CH_3_), 35.8 (CH_2_), 50.3 (CH_2_), 55.8 (CH_3_), 104.2 (CH), 115.0 (CH), 121.2 (CH), 121.7 (2CH), 131.7 (C), 144.3 (C), 145.9 (C), 150.1 (2CH), 153.9 (C), 156.2 (C), 162.2 (C). HRMS (C_17_H_18_N_4_O_2_S) *m*/*z*: calculated 343.1223 (M + H^+^), 365.1043 (M + Na^+^); found 343.1223 (M + H^+^), 365.1023 (M + Na^+^).

3-ethyl-1-(6-methoxybenzo[d]thiazol-2-yl)-1-((4-methyl-1*H*-imidazol-5-yl)methyl)-urea (**10**). Compound **10** was synthesized following method B as previously described using compound **4** (250 mg, 0.911 mmol), ethylisothiocyanate (180 µL, 2.27 mmol), and triethylamine (316 µL, 2.27 mmol) in CH_2_Cl_2_ (13 mL). The crude residue was purified via flash column chromatography (DCM/MeOH 20:1–9:1) to yield 289 mg (0.838 mmol, 92%) of compound **10**. IR (KBr): 3210, 2937, 1649, 1516, 1469, 1271, 1063, 806 cm^−1^. ^1^H-NMR (400 MHz, DMSO-d_6_): δ 1.16 (3H, t, J = 7.2 Hz), 2.37 (1H, s), 3.24 (2H, m), 3.76 (3H, s), 5.13 (2H, s), 6.94 (1H, dd, J = 2.4 and 9.0 Hz), 7.42 (1H, s), 7.56 (2H, m), 8.91 (1H, bs), 11.9 (1H, bs). ^13^C-NMR (100 MHz, DMSO-d_6_): δ 9.7 (CH_3_), 15.3 (CH_3_), 35.7 (CH_2_), 43.3 (CH_2_), 56.0 (CH_3_), 104.8 (CH), 114.6 (CH), 120.1 (CH), 132.1 (C), 132.5 (C), 133.8 (C), 134.4 (CH), 143.1 (C), 156.1 (C), 156.6 (C), 159.6 (C). HRMS (C_16_H_19_N_5_O_2_S) *m*/*z*: calculated 346.1332 (M + H^+^), 368.1152 (M + Na^+^); found 346.1327 (M + H^+^), 368.1115 (M + Na^+^).

1-benzyl-3-ethyl-1-(6-methoxybenzo[d]thiazol-2-yl)urea (**11**). Compound **11** was synthesized following method B as previously described using compound **5** (260 mg, 0.962 mmol), ethylisothiocyanate (190 µL, 2.40 mmol), and triethylamine (334 µL, 2.40 mmol) in CH_2_Cl_2_ (12 mL). The crude residue was purified via flash column chromatography (EtOAc/hexane 1:3–DCM/MeOH 20:1) to yield 308 mg (0.904 mmol, 94%) of compound **11**. IR (KBr): 3263, 2930, 1686, 1552, 1526, 1357, 1230, 1061, 827 cm^−1^. ^1^H-NMR (400 MHz, CDCl_3_): δ 1.28 (3H, bt, J = 7.2 Hz), 3.47 (2H, bq, J = 5.6 Hz), 5.23 (2H, s), 6.97 (1H, dd, J = 2.8 and 8.8 Hz), 7.14 (bd, J = 2.8 Hz), 7.20–7.36 (5H, m), 7.60 (1H, d, J = 9.2 Hz), 9.40 (1H, bs). ^13^C-NMR (100 MHz, CDCl_3_): δ 15.1 (CH_3_), 35.8 (CH_2_), 51.1 (CH_2_), 55.8 (CH_3_), 104.2 (CH), 114.7 (CH), 121.0 (CH), 126.9 (2CH), 127.5 (CH), 128.7 (2CH), 132.1 (C), 136.6 (C), 144.2 (C), 154.3 (C), 153.3 (C), 162.7 (C). HRMS (C_18_H_19_N_3_O_2_S) *m*/*z*: calculated 342.1271 (M + H+), 364.1090 (M + Na^+^); found 342.1264 (M + H^+^), 364.1084 (M + Na^+^).

3-ethyl-1-(6-methoxybenzo[d]thiazol-2-yl)-1-(naphthalen-2-ylmethyl)urea (**12**). Compound **12** was synthesized following method B as previously described using compound **6** (266 mg, 0.830 mmol), ethylisothiocyanate (170 µL, 2.07 mmol), and triethylamine (289 µL, 2.07 mmol) in CH_2_Cl_2_ (12 mL). The crude residue was purified via flash column chromatography (EtOAc/hexane 1:4–DCM/MeOH 25:1) to yield 299 mg (0.763 mmol, 92%) of compound **12**. IR (KBr): 3378, 2932, 1684, 1525, 1456, 1230, 1062, 811 cm^−1^. ^1^H-NMR (400 MHz, CDCl_3_): 1.31 (3H, t, J = 6.8 Hz), 3.51 (2H, q, J = 6.8 Hz), 3.79, (3H, s), 5.40 (2H, s), 6.97 (1H, dd, J = 2.4 and 8.6 Hz), 7.11 (1H, d, J = 2.4 Hz), 7.40–7.47 (3H, m), 7.60 (1H, d, J = 9.2 Hz), 7.55–7.81 (4H, m), 9.46 (1H, bs). ^13^C-NMR (100 MHz, CDCl_3_): 15.1 (CH_3_), 35.8 (CH_2_), 51.2 (CH_2_), 55.8 (CH_3_), 104.2 (CH), 114.8 (CH), 121.1 (CH), 125.0 (CH), 125.7 (CH), 125.9 (CH), 126.2 (CH), 127.7 (CH), 127.8 (CH), 128.6 (CH), 132.1 (C), 132.8 (C), 133.3 (C), 134.1 (C), 144.2 (C), 154.4 (C), 156.4 (C), 162.7 (C). HRMS (C_22_H_21_N_3_O_2_S) *m*/*z*: calculated 392.1427 (M + H^+^), 414.1247 (M + Na^+^); found 392.1427 (M + H^+^), 414.1231 (M + Na^+^).

*N*-(6-methoxybenzo[d]thiazol-2-yl)-*N*-(pyridin-2-ylmethyl)formamide (**13**). Compound **13** was synthesized following method C as previously described using compound **1** (269 mg, 0.992 mmol), formic acid (75 µL, 1.98 mmol), EDC (380 mg, 1.98 mmol), HOBt (268 mg, 1.98 mmol), and DMAP (242 mg, 1.98 mmol) in CH_2_Cl_2_ (22 mL). The crude residue was purified via flash column chromatography (EtOAc/hexane 1:3–DCM/MeOH 15:1) to yield 211 mg (0.705 mmol, 71%) of compound **13**. IR (KBr): 3425, 2932, 1703, 1612, 1520, 1350, 1220, 812 cm^−1^. ^1^H-NMR (400 MHz, acetone-d_6_): δ 3.85 (3H, s), 3.87 (s, minor), 5.32 (s, minor), 5.48 (2H, s), 6.99 (1H, dd, J = 2.4 and 8.8 Hz), 7.24 (1H, bt, J = 7.6 Hz), 7.47 (1H, d, J = 2.4 Hz), 7.53 (1H, d, J = 8 Hz), 7.61 (1H, d, J = 8.8 Hz), 7.76–7.69 (1H, m), 8.49 (1H, d, J = 4.0 Hz), 9.01 (1H, s), 9.39 (s, minor). ^13^C-NMR (100 MHz, DMSO-d_6_): δ 52.0 (CH_2_), 55.9 (CH_3_), 105.1 (CH), 115.6 (CH), 121.9 (CH), 122.1 (CH), 123.5 (CH), 133.4 (CH, minor), 133.5 (C), 137.4 (CH), 138.7 (CH, minor), 141.9 (C), 149.5 (CH), 155.3 (C), 156.3 (C), 156.9 (C), 163.7 (C, minor), 164.6 (C). HRMS (C_15_H_13_N_3_O_2_S) *m*/*z*: calculated 300.0801 (M + H^+^), 322.0621 (M + Na^+^); found 300.0802 (M + H^+^), 322.0622 (M + Na^+^).

*N*-(6-methoxybenzo[d]thiazol-2-yl)-*N*-(pyridin-3-ylmethyl)formamide (**14**). Compound **14** was synthesized following method C as previously described using compound **2** (257 mg, 0.948 mmol), formic acid (72 µL, 1.90 mmol), EDC (363 mg, 1.90 mmol), HOBt (256 mg, 1.90 mmol), and DMAP (232 mg, 1.90 mmol) in CH_2_Cl_2_ (22 mL). The crude residue was purified via flash column chromatography (EtOAc/hexane 1:3–DCM/MeOH 15:1) to yield 199 mg (0.664 mmol, 70%) of compound **14**. IR (KBr): 3434, 2919, 1700, 1605, 1515, 1373, 1255, 1026, 801 cm^−1^. ^1^H-NMR (400 MHz, acetone-d_6_): δ 3.79 (3H, s, minor), 3.86 (3H, s), 5.27 (s, minor), 5.49 (2H, s), 7.04 (1H, dd, J = 2.4 and 9.2 Hz), 7.35 (1H, dd, J = 4.5 and 7.4 Hz), 7.50 (1H, d, J = 2.8 Hz), 7.71 (1H, d, J = 9.2 Hz), 7.92 (1H, d, J = 7.2 Hz), 8.49 (1H, d, J = 5.2 Hz), 8.72 (bs, minor), 8.79 (1H, bs), 9.11 (1H, s), 9.26 (s, minor). ^13^C-NMR (100 MHz, DMSO-d_6_): δ 44.6 (CH_2_, minor), 48.7 (CH_2_), 55.2 (CH_3_), 103.9 (CH), 104.7 (CH, minor), 115.2 (CH), 122.1 (CH), 124.1 (CH), 133.6 (C), 135.3 (CH), 142.1 (C), 148.9 (CH), 149.6 (CH), 154.7 (C), 157.2 (C), 161.2 (C), 162.7 (C). HRMS (C_15_H_13_N_3_O_2_S) *m*/*z*: calculated 300.0801 (M + H^+^), 322.0621 (M + Na^+^); found 300.0790 (M + H^+^), 322.0623 (M + Na^+^).

6-methoxy-*N*-(pyridin-4-ylmethyl)benzo[d]thiazol-2-amine (**15**). Compound **15** was synthesized following method C as previously described using compound **3** (263 mg, 0.948 mmol), formic acid (73 µL, 1.94 mmol), EDC (372 mg, 1.94 mmol), HOBt (262 mg, 1.94 mmol), and DMAP (237 mg, 1.94 mmol) in CH_2_Cl_2_ (22 mL). The crude residue was purified via flash column chromatography (EtOAc/hexane 1:3–DCM/MeOH 15:1) to yield 218 mg (0.728 mmol, 75%) of compound **15**. IR (KBr): 3420, 2951, 1695, 1622, 1540, 1403, 1220, 1057, 815 cm^−1^. ^1^H-NMR (400 MHz, acetone-d_6_): δ 3.65 (s, minor), 3.72 (3H, s), 5.15 (s, minor), 5.35 (2H, s), 6.91 (1H, dd, J = 2.8 and 8.8 Hz), 6.74 (d, minor), 7.37 (1H, d, J = 2.8 Hz), 7.56 (2H, d, J = 8.4 Hz), 7.72 (d, minor), 7.77 (1H, d, J = 8.8 Hz), 8.46 (2H, d, J = 8.4 Hz), 8.97 (1H, s), 9.12 (s, minor). ^13^C-NMR (100 MHz, DMSO-d_6_): δ 46.2 (CH_2_, minor), 47.6 (CH_2_), 55.9 (CH_3_), 105.4 (CH), 113.5 (CH), 119.2 (CH), 122.2 (2CH), 131.4 (C), 144.2 (C), 146.1 (C), 148.9 (2CH), 156.3 (C), 157.8 (C), 162.1 (C, minor), 162.8 (C). HRMS (C_15_H_13_N_3_O_2_S) *m*/*z*: 300.0801 (M + H^+^), 322.0621 (M + Na^+^); found 300.0801 (M + H^+^), 322.0619 (M + Na^+^).

*N*-(6-methoxybenzo[d]thiazol-2-yl)-*N*-((4-methyl-1*H*-imidazol-5-yl)methyl)formamide (**16**). Compound **16** was synthesized following method C as previously described using compound **4** (250 mg, 0.911 mmol), formic acid (69 µL, 1.82 mmol), EDC (349 mg, 1.82 mmol), HOBt (246 mg, 1.82 mmol), and DMAP (223 mg, 1.82 mmol) in CH_2_Cl_2_ (20 mL). The crude residue was purified via flash column chromatography (EtOAc/hexane 1:1–DCM/MeOH 9:1) to yield 190 mg (0.629 mmol, 69%) of compound **16**. IR (KBr): 3400, 3140, 2948, 1605, 1541, 1463, 1224, 1049, 812 cm^−1^. ^1^H-NMR (400 MHz, CDCl_3_): δ 2.44 (3H, s), 3.76 (3H, s), 5.05 (s, minor), 5.18 (2H, s), 6.85 (2H, m), 7.15 (1H, d, J = 2.8 Hz), 7.45 (1H, s), 8.91 (1H, s), 9.15 (s, minor). ^13^C-NMR (100 MHz, MeOD): δ 10.5 (CH_3_), 39.4 (CH_3_), 54.8 (CH_2_), 104.9 (CH), 112.9 (CH), 117.9 (CH), 128.1 (C), 128.2 (C), 130.9 (C), 133.4 (CH), 145.8 (C), 155.4 (C), 161.9 (C), 165.9 (C). HRMS (C_14_H_14_N_4_O_2_S) *m*/*z*: calculated 303.0910 (M + H^+^); found 303.0915 (M + H^+^).

*N*-benzyl-*N*-(6-methoxybenzo[d]thiazol-2-yl)formamide (**17**). Compound **17** was synthesized following method C as previously described using compound **5** (265 mg, 0.980 mmol), formic acid (74 µL, 1.96 mmol), EDC (376 mg, 1.96 mmol), HOBt (265 mg, 1.96 mmol), and DMAP (240 mg, 1.96 mmol) in CH_2_Cl_2_ (20 mL). The crude residue was purified via flash column chromatography (EtOAc/hexane 1:1–DCM/MeOH 20:1) to yield 219 mg (0.735 mmol, 75%) of compound **17**. IR (KBr): 3467, 1701, 1515, 1465, 1375, 1259, 1201, 1068, 961, 814 cm^−1^. ^1^H-NMR (400 MHz, DMSO-d_6_): δ 3.78 (3H, s), 5.17 (s, minor), 5.35 (2H, s), 7.00 (1H, dd, J = 2.4 and 8.4 Hz), 7.24 (1H, m), 7.33 (2H, t, J = 7.2 Hz), 7.38 (2H, d, J = 7.2 Hz), 7.56 (1H, d, J = 2.8 Hz), 7.64 (1H, d, J = 8.4 Hz), 9.01 (1H, s), 9.37 (s, minor). ^13^C-NMR (100 MHz, DMSO-d_6_): δ 50.8 (CH2), 56.1 (CH_3_), 105.0 (CH), 115.6 (CH), 122.3 (CH), 127.7 (2CH), 127.9 (CH), 128.9 (2CH), 133.5 (C), 137.5 (C), 142.0 (C), 155.3 (C), 156.9 (C), 164.2 (C). HRMS (C_16_H_14_N_2_O_2_S) *m*/*z*: calculated 299.0849 (M + H^+^), 321.0668 (M + Na^+^); found 299.0865 (M + H^+^), 321.0666 (M + Na^+^).

*N*-(6-methoxybenzo[d]thiazol-2-yl)-*N*-(naphthalen-2-ylmethyl)formamide (**18**). Compound **18** was synthesized following method C as previously described using compound **6** (260 mg, 0.811 mmol), formic acid (61 µL, 1.62 mmol), EDC (311 mg, 1.61 mmol), HOBt (219 mg, 1.62 mmol), and DMAP (240 mg, 1.62 mmol) in CH_2_Cl_2_ (20 mL). The crude residue was purified via flash column chromatography (EtOAc/hexane 1:3–DCM/MeOH 20:1) to yield 198 mg (0.568 mmol, 70%) of compound **18**. IR (KBr): 3435, 1674, 1515, 1469, 1263, 1210, 831 cm^−1^. ^1^H-NMR (400 MHz, DMSO-d_6_): 3.77 (3H, s), 5.36 (s, minor), 5.51 (2H, s), 6.99 (1H, dd, J = 2.4 and 9.0 Hz), 7.45–7.47 (2H, m), 7.53–7.56 (2H, m), 7.63 (2H, d, J = 9.0 Hz), 7.84–7.89 (4H, m), 9.09 (1H, s), 9.36 (s, minor). ^13^C-NMR (100 MHz, DMSO-d_6_): 51.1 (CH_2_), 56.1 (CH_3_), 105.0 (CH), 115.6 (CH), 122.2 (CH), 125.9 (CH), 126.2 (CH), 126.5 (CH), 126.8 (CH), 127.9 (CH), 128.1 (CH), 128.6 (CH), 132.8 (C), 133.2 (C), 133.5 (C), 135.1 (C), 142.0 (C), 155.3 (C), 156.9 (C), 164.3 (C). HRMS (C_20_H_16_N_2_O_2_S) *m*/*z*: calculated 349.1005 (M + H^+^), 371.0825 (M + Na^+^); found 349.0994 (M + H^+^), 371.0823 (M + Na^+^).

*N*-(6-methoxybenzo[d]thiazol-2-yl)-*N*-(pyridin-2-ylmethyl)acetamide (**19**). Compound **19** was synthesized following method D as previously described using compound **1** (260 mg, 0.811 mmol), acetic anhydride (52 µL, 1.38 mmol), and DMAP (169 mg, 1.38 mmol) in CH_2_Cl_2_ (12 mL). The crude residue was purified via flash column chromatography (EtOAc/hexane 1:3–3:2) to yield 280 mg (0.895 mmol, 97%) of compound **19**. IR (KBr): 3436, 1672, 1603, 1472, 1280, 1227, 1030, 829 cm^−1^. ^1^H-NMR (400 MHz, DMSO-d_6_): δ 2.38 (3H, s), 3.77 (3H, s), 5.58 (2H, s), 6.95 (1H, dd, J = 2.0 and 8.8 Hz), 7.26 (1H, bt, J = 6.8 Hz), 7.39 (2H, d, J = 8 Hz), 7.51 (2H, d, J = 2.4 Hz), 7.55 (1H, d, J = 8.4 Hz), 7.77 (1H, dt, J = 1.6 and 7.6 Hz), 8.46 (1H, bd, J = 5.2 Hz). ^13^C-NMR (100 MHz, DMSO-d_6_): δ 23.5 (CH_3_), 52.4 (CH_2_), 56.1 (CH_3_), 104.7 (CH), 115.3 (CH), 121.9 (CH), 122.0 (CH), 123.1 (CH), 134.4 (C), 137.5 (CH), 142.1 (C), 149.7 (CH), 156.2 (C), 156.7 (C), 157.6 (C), 172.1 (C). HRMS (C_16_H_15_N_3_O_2_S) *m*/*z*: calculated 314.0958 (M + H^+^), 336.0777 (M + Na^+^); found 314.0946 (M + H^+^), 336.0773 (M + Na^+^).

*N*-(6-methoxybenzo[d]thiazol-2-yl)-*N*-(pyridin-3-ylmethyl)acetamide (**20**). Compound **20** was synthesized following method D as previously described using compound **2** (252 mg, 0.930 mmol), acetic anhydride (53 µL, 1.39 mmol), and DMAP (170 mg, 1.39 mmol) in CH_2_Cl_2_ (12 mL). The crude residue was purified via flash column chromatography (EtOAc/hexane 1:3–3:2) to yield 285 mg (0.911 mmol, 98%) of compound **20**. IR (KBr): 3435, 1663, 1601, 1515, 1395, 1233, 1029, 816 cm^−1^. ^1^H-NMR (400 MHz, CDCl_3_): δ 2.37 (3H, s), 3.87 (3H, s), 5.56 (2H, s), 7.01 (1H, dd, J = 2.4 and 8.8 Hz), 7.29–7.23 (2H, m), 7.59 (1H, bd, J = 7.6 Hz), 7.66 (1H, bd, J = 8.8 Hz), 8.53 (1H, bd, J = 4.8 Hz), 8.61 (1H, bs). ^13^C-NMR (100 MHz, CDCl_3_): δ 23.3 (CH_3_), 49.2 (CH_2_), 55.8 (CH_3_), 103.8 (CH), 115.2 (CH), 122.2 (CH), 123.8 (CH), 132.1 (C), 134.4 (CH), 134.8 (C), 148.4 (C), 149.2 (2CH), 157.0 (C), 156.3 (C), 170.4 (C). HRMS (C_16_H_15_N_3_O_2_S) *m*/*z*: calculated 314.0958 (M + H^+^), 336.0777 (M + Na^+^); found 314.0948 (M + H^+^), 336.0779 (M + Na^+^).

*N*-(6-methoxybenzo[d]thiazol-2-yl)-*N*-(pyridin-4-ylmethyl)acetamide (**21**). Compound **21** was synthesized following method D as previously described using compound **3** (262 mg, 0.967 mmol), acetic anhydride (55 µL, 1.45 mmol), and DMAP (177 mg, 1.45 mmol) in CH_2_Cl_2_ (12 mL). The crude residue was purified via flash column chromatography (EtOAc/hexane 1:3–3:2) to yield 300 mg (0.967 mmol, 99%) of compound **21**. IR (KBr): 3435, 1686, 1599, 1499, 1472, 1221, 1031, 815 cm^−1^. ^1^H-NMR (400 MHz, CDCl_3_): δ 2.32 (3H, s), 3.86 (3H, s), 5.55 (2H, s), 6.99 (1H, dd, J = 2.8 and 8.4 Hz), 7.15 (2H, d, J = 6.0 Hz), 7.27 (1H, d, J = 2.8 Hz), 7.62 (2H, d, J = 8.4 Hz), 8.56 (2H, d, J = 6.0 Hz). ^13^C-NMR (100 MHz, CDCl_3_): δ 23.2 (CH_3_), 50.5 (CH_2_), 55.8 (CH_3_), 103.7 (CH), 115.2 (CH), 122.1 (CH), 122.2 (2CH), 134.8 (C), 142.1 (C), 145.4 (C), 150.4 (2CH), 157.0 (C), 157.1 (C), 170.4 (C). HRMS (C_16_H_15_N_3_O_2_S) *m*/*z*: calculated 314.0958 (M + H^+^), 336.0777 (M + Na^+^); found 314.0941 (M + H^+^), 336.0776 (M + Na^+^).

*N*-(6-methoxybenzo[d]thiazol-2-yl)-*N*-((4-methyl-1*H*-imidazol-5-yl)methyl)acetamide (**22**). Compound **22** was synthesized following method D as previously described using compound **4** (238 mg, 0.868 mmol), acetic anhydride (49 µL, 1.30 mmol), and DMAP (159 mg, 1.30 mmol) in CH_2_Cl_2_ (12 mL). The crude residue was purified via flash column chromatography (EtOAc/hexane 1:3–DCM/MeOH 9:1) to yield 263 mg (0.833 mmol, 96%) of compound **22**. IR (KBr): 3481, 3164, 1647, 1470, 1407, 1242, 1069, 821 cm^−1^. ^1^H-NMR (400 MHz, DMSO-d_6_): δ 2.31 (3H, s), 2.72 (3H, s), 3.77 (3H, s), 5.23 (2H, s), 7.00 (1H, dd, J = 2.4 and 8.8 Hz), 7.40 (1H, s), 7.49 (1H, d, J = 2.4 Hz), 7.62 (1H, d, J = 8.8 Hz). ^13^C-NMR (100 MHz, DMSO-d_6_): δ 9.9 (CH_3_), 24.0 (CH_3_), 44.5 (CH_2_), 56.0 (CH_3_), 104.7 (CH), 115.3 (CH), 121.8 (CH), 134.0 (CH), 134.4 (C), 134.4 (C), 135.6 (C), 142.2 (C), 156.6 (C), 157.1 (C), 172.3 (C). HRMS (C_15_H_16_N_4_O_2_S) *m*/*z*: calculated 317.1067 (M + H^+^); found 317.1066 (M + H^+^).

*N*-benzyl-*N*-(6-methoxybenzo[d]thiazol-2-yl)acetamide (**23**). Compound **23** was synthesized following method D as previously described using compound **5** (260 mg, 0.959 mmol), acetic anhydride (54 µL, 1.43 mmol), and DMAP (176 mg, 1.43 mmol) in CH_2_Cl_2_ (13 mL). The crude residue was purified via flash column chromatography (EtOAc/hexane 1:3–2:1) to yield 297 mg (0.950 mmol, 99%) of compound **23**. IR (KBr): 3448, 1663, 1601, 1512, 1443, 1234, 1029, 817 cm^−1^. ^1^H-NMR (400 MHz, CDCl_3_): δ 2.33 (3H, s), 3.86 (3H, s), 5.57 (2H, bs), 7.00 (1H, dd, J = 2.4 and 8.8 Hz), 7.20–7.41 (6H, m), 7.66 (1H, d, J = 8.8 Hz). ^13^C-NMR (100 MHz, CDCl_3_): δ 23.3 (CH_3_), 51.3 (CH_2_), 55.8 (CH_3_), 103.8 (CH), 115.0 (CH), 122.2 (CH), 126.3 (CH), 127.6 (2CH), 128.9 (2CH), 134.9 (C), 136.4 (C), 142.3 (C), 156.8 (C), 157.2 (C), 171.0 (C). HRMS (C_17_H_16_N_2_O_2_S) *m*/*z*: calculated 313.1005 (M + H^+^), 335.0825 (M + Na^+^); found 313.0995 (M + H^+^), 335.0821 (M + Na^+^).

*N*-(6-methoxybenzo[d]thiazol-2-yl)-*N*-(naphthalen-2-ylmethyl)acetamide (**24**). Compound **24** was synthesized following method D as previously described using compound **6** (270 mg, 0.843 mmol), acetic anhydride (48 µL, 1.26 mmol), and DMAP (154 mg, 1.26 mmol) in CH_2_Cl_2_ (12 mL). The crude residue was purified via flash column chromatography (EtOAc/hexane 1:3–2:1) to yield 299 mg (0.826 mmol, 98%) of compound **24**. IR (KBr): 3435, 1664, 1511, 1400, 1235, 1030, 818 cm^−1^. ^1^H-NMR (400 MHz, CDCl_3_): 2.36 (3H, s), 3.87 (3H, s), 5.73 (2H, s), 7.01 (1H, dd, J = 2.4 and 9.0 Hz), 7.30 (1H, d, J = 2.4 Hz), 7.39 (1H, d, J = 8.4 Hz), 7.44–7.46 (2H, m), 7.61 (1H, s), 7.63 (1H, d, J = 9.2 Hz), 7.74 (1H, m), 7.81 (2H, m). ^13^C-NMR (100 MHz, CDCl_3_): 23.4 (CH_3_), 51.5 (CH_2_), 55.8 (CH_3_), 103.8 (CH), 115.0 (CH), 122.2 (CH), 124.4 (CH), 124.7 (CH), 126.1 (CH), 126.4 (CH), 127.7 (CH), 127.8 (CH), 128.9 (CH), 132.8 (C), 133.6 (C), 133.9 (C), 134.9 (C), 142.0 (C), 156.9 (C), 164.3 (C), 171.1 (C). HRMS (C_21_H_18_N_2_O_2_S) *m*/*z*: calculated 363.1162 (M + H^+^), 385.0981 (M + Na^+^); found 363.1165 (M + H^+^), 385.0978 (M + Na^+^).

### 2.2. Determination of Aqueous Solubility

The aqueous solubility of compounds was measured in a Helios Alfa Spectrophotometer. Calibration lines at the maximum wavelength of absorbance were determined for each compound. Then, following the shake-flask method, a suspension of 5 mg of each compound in 500 µL in pH 7.0 buffer was stirred for 72 h at room temperature. The mixture was then filtrated over a 45 µm filter to remove the insoluble fraction. The concentration of the compound in the resulting saturated solution was determined via UV absorbance measurement and interpolation in the calibration line. The aqueous solubility values are given as the average of three independent measurements. 

### 2.3. Calculation of Properties

The total polar surface area (TPSA) and the logarithm of the partition coefficient (Log P*_o_*_/*w*_) were calculated using SwissADME software [42,43]. TPSA calculations follow a methodology based on a sum of fragment-based contributions [44]. The consensus Log P*_o_*_/*w*_, which is the arithmetic mean of the values predicted by the five proposed methods applied by Swiss-ADME, was used in this work.

### 2.4. Biology

#### 2.4.1. Cell Culture Conditions

The cell lines were obtained from ATTC (Manassas, VA, USA). HeLa (human cervix epithelioid carcinoma), MCF7 (human breast adenocarcinoma), HepG2 (human hepatocellular carcinoma), U87 MG (human glioblastoma), T98G (human glioblastoma), GL261 (murine glioma), LNCaP (human prostate cancer), PC-3 (human prostate cancer), and HEK-293 (human embryonic kidney) were grown in Dulbecco’s modified Eagle’s medium (DMEM, Gibco, Waltham, MA, USA), supplemented with 10% (*v*/*v*) heat-inactivated fetal bovine serum (HIFBS, Sigma-Aldrich, St. Louis, MO, USA)), 2 mM L-glutamine, 100 μg/mL streptomycin, and 100 units/mL penicillin (Gibco, Waltham, MA, USA) at 37 °C in saturated humidity atmosphere containing 95% air and 5% CO_2_. Cell line HT-29 (human colon carcinoma) was grown in Roswell Park Memorial Institute (RPMI) 1640 medium (Gibco, Whaltman, MA, USA) supplemented with 10% HIFBS, 100 μg/mL streptomycin, and 100 units/mL penicillin (Gibco, Whaltman, MA, USA) at 37 °C in humidified 95% air and 5% CO_2_ atmosphere. MycoAlert kit (Lonza, Norwest, Australia) was used to routinely check the presence of mycoplasma, and only mycoplasma-free cells were employed in the experiments.

#### 2.4.2. Cell Growth Inhibition Assay

Cells were plated in 96-well plates (100 µL/well) at the following concentrations: 30,000 cells/mL (HepG2, U87 MG, T98G, C6, LNCaP, PC3, and HT-29 cells) or 15,000 cells/mL (HeLa and MCF-7 cells). Previous experiments were performed to choose the optimal cell concentration (70–80% confluence) and avoid the formation of cell aggregates. Cells were incubated in complete DMEM or RPMI 1640 medium (see above) at 37 °C and 5% CO_2_ atmosphere for 24 h to allow for cell attachment to the plate. Then, every compound was added (10 µL/well) to a final concentration of 10 µM. Non-treated cells were used as negative controls. The antiproliferative activity of compounds was measured 72 h after the treatments using the MTT reagent (3-[4,5-dimethylthiazol-2-yl]-2,5-diphenyltetrazolium bromide), (MTT Thiazolyl Blue Tetrazolium Bromide, Sigma-Aldrich, St. Louis, MO, USA) dissolved in PBS at 5 mg/mL. Compounds showing antiproliferative effects at 10 µM were selected to determine their IC_50_ value. For that purpose, compounds were used at different concentrations ranging from 10^−10^ to 10^−2^ M. Measurements were performed in triplicate, and each experiment was repeated three times. The IC_50_ value was determined for every compound using Origin software (OriginLab, Washington, USA). To study the sensitivity to MDR efflux pumps of compounds, the IC_50_ values were determined by following the same procedure but in the presence of verapamil (final concentration of 10 µM).

#### 2.4.3. Tubulin Polymerization Inhibition

Microtubular protein was isolated from calf brain following the modified Shelanski method, based on temperature-dependent assembly/disassembly of tubulin [45]. The protein was stored at −80 °C, and its concentration was determined using the Bradford method before each use. Tubulin polymerization, which occurs when heating a buffered tubulin sample from 4 °C to 37 °C, is accompanied by an increase in the absorbance (at 450 nm) of the sample that was monitored by a Helios α spectrophotometer. The samples containing 1.5 mg/mL of microtubular protein at pH 6.7 buffer (0.1 M MES, 1 mM β-ME, 1 mM EGTA, 1 mM MgCl_2_, and 1.5 mM GTP) and the ligand at 10 µM (except for control samples) were incubated at 20 °C for 30 min to allow for the ligand to bind to the tubulin, and were subsequently cooled on ice for 10 min to make sure that polymerization did not take place at the initial time point. Then, the temperature was adjusted to 4 °C, and the temperature being shifted to 37 °C allowed for the assembly process of tubulin. The turbidity caused by tubulin polymerization was measured by the increased absorbance at 450 nm. The increase in absorbance obtained for the control sample (without the ligand), which we considered to be 100% tubulin polymerization (0% inhibition), compared with the increase in absorbance determined for the sample containing ligands yielded the degree of tubulin polymerization inhibition (TPI), which is expressed as a percentage value. For the compounds displaying TPI values over 50% at 10 µM, the IC_50_ value for TPI was calculated. Measurements were performed in triplicate in two independent experiments using microtubular protein from different preparations.

#### 2.4.4. Immunofluorescence Experiments

The 8 × 10^4^ HeLa, MCF7, and U87 MG cells were seeded on 0.01% poly-L-lysine pre-coated square glass coverslips (22 mm^2^) deposited on 6-well plates (1 coverslip/well) and incubated in complete DMEM medium (see above) at 37 °C and 5% CO_2_ atmosphere for 24 h. Then, the culture medium was replaced by fresh complete DMEM, and cells were incubated in the presence or absence of selected compounds for 24 h. Untreated cells were used as negative controls. The concentration of the compounds was selected according to their IC_50_ value for the antiproliferative activity in each cell line as follows: 50 nM if IC_50_ is in the low nanomolar range (below 10 nM); 100 nM when IC_50_ is in the two-digit nanomolar; 200 nM when IC_50_ is near 100 nM, and 500 nM for compounds with IC_50_ near 200 nM. In that way, compounds with similar antiproliferative potencies were tested at the same concentrations. HeLa cells were treated with 200 nM of compounds **8** and **14** and 500 nM of compound **9**; MCF7 cells were treated with 100 nM of compounds **8** and **14** and 500 nM of compound **9**; and U87 MG cells were treated with 50 nM of compound **8** and 100 nM of compounds **9** and **14**. After incubation, medium was removed and coverslips were washed three times with PBS, fixed in 4% formaldehyde in PBS for 10 min, permeabilized with 0.5% Triton X-100 (Sigma-Aldrich, St. Louis, MO, USA) in PBS for 90 s at 4 °C, and blocked with 10% BSA in PBS for 30 min. After four washes with PBS, the coverslips were incubated for 1 h with anti-α-tubulin mouse monoclonal antibody (Sigma-Aldrich, St. Louis, MO, USA), and diluted 1:200 in PBS containing 3% BSA. After four washes wish PBS, coverslips were incubated, in darkness, with fluorescent secondary antibody Alexa Fluor 488 goat anti-mouse IgG (Molecular Probes, Invitrogen, Eugenen, OR, USA), and diluted 1:400 in PBS containing 1% BSA for 1.5 h. After four washes with PBS, a drop of ProLong™ Gold Antifade Mountant containing DAPI (ThermoFisher, Waltham, MA, USA) was added for cell nuclei staining. Samples were analyzed via confocal microscopy using a LEICA SP5 microscope DMI-6000V model coupled to a LEICA LAS AF software computer. 

#### 2.4.5. Cell Cycle Analysis

An amount of 8 × 10^4^ cells/mL (HeLa, MCF7, or U87 MG cells) were seeded in 6-well plates (2 mL/well) and incubated in complete DMEM medium (see above) at 37 °C and 5% CO_2_ atmosphere for 24 h. Then, the medium was replaced with fresh complete DMEM in the presence or absence of the selected compounds (**8**, **9**, and **14**). The concentration of the compounds was selected according to their IC_50_ value for the antiproliferative activity in each cell line (see above, Section 2.4.4). HeLa cells were treated with 200 nM of compounds **8** and **14** and 500 nM of compound **9**; MCF7 cells were treated with 100 nM of compounds **8** and **14** and 500 nM of compound **9**; and U87 MG cells were treated with 50 nM of compound **8** and 100 nM of compounds **9** and **14**. Untreated cells were used as negative controls. Cells were harvested 24, 48, or 72 h following treatment and fixed in ice-cold ethanol/PBS (7:3) overnight. Cells were then washed twice with PBS, suspended in PBS, and incubated overnight in darkness with 0.2 mg/mL RNase A (Sigma-Aldrich, St. Louis, MO, USA), 50 µg/mL propidium iodide (Sigma-Aldrich, St. Louis, MO, USA), and Triton 10× at room temperature. BD Accuri™ C6 Plus Flow Cytometer (BD Biosciences) was used to analyze samples, and BD Accuri™ C6 Software (version 1.0.264.21) was used for data analysis.

#### 2.4.6. Apoptotic Cell Death Quantification

Annexin V-FITC/PI apoptosis detection kit (Immunostep, Salamanca, Spain) was used to quantify HeLa, MCF7, or U87 MG cells by following the manufacturer’s guidelines. An amount of 5 × 10^4^ cells/mL were seeded in 12-well plates (1 mL/well) and incubated in complete DMEM medium (see above) at 37 °C and 5% CO_2_ atmosphere for 24 h. Then, the medium was replaced with fresh complete DMEM in the presence or absence of the selected compounds (**8**, **9**, and **14**). The concentration of the compounds was selected according to their IC_50_ value for the antiproliferative activity in each cell line (see above, Section 2.4.4). HeLa cells were treated with 200 nM of compounds **8** and **14** and 500 nM of compound **9**; MCF7 cells were treated with 100 nM of compounds **8** and **14** and 500 nM of compound **9**; and U87 MG cells were treated with 50 nM of compound **8** and 100 nM of compounds **9** and **14**. Untreated cells were used as negative controls. After 72 h of incubation, cells were collected, centrifugated, resuspended in the Annexin V binding buffer, and stained with Annexin V-FITC/PI. Cells were then incubated in darkness for 15 min at room temperature. Samples were analyzed using BD Accuri™ C6 Plus Flow Cytometer (BD Biosciences), and acquired data were analyzed using BD Accuri™ C6 Software (version 1.0.264.21).

#### 2.4.7. Lactate Dehydrogenase Assay

CytoTox96^®^ Non-Radioactive Cytotoxicity Assay kit (Promega, Madison, WI, USA) was used to measure the release of lactate dehydrogenase (LDH) by following the previously described method in [46]. Briefly, U87 MG or HEK-293 cells were incubated for 72 h with compounds **8**, **9**, or **14** at concentrations ranging from 10^−9^ to 10^−6^ M for U87 MG cells, and from 10^−4^ to 10^−3^ M for HEK-293 cells. After treatment, the supernatants were collected, and the intact attached cells were lysed using 0.1% (*w*/*v*) Triton X-100 in (0.9%) NaCl solution. Both the LDH released to culture media, as well as the LDH content within the cells, were determined spectrophotometrically at 490 nm on a 96-well plate reader (Infinite 200, Tecan, Salzburg, Austria) by following the manufacturer’s instructions. LDH release was defined by the ratio of LDH released/total LDH present in the cells, with the total LDH being 100%. All the samples were run in quadruplicate.

### 2.5. Computational Studies

Structural preferences for the unsubstituted scaffolds (*N*-benzylbenzothiazole-2-amine, *N*-benzyl-*N*-(benzothiazole-2-yl)formamide, *N*-benzyl-*N*-(benzothiazole-2-yl)acetamide, and *N*-benzyl-*N*-(benzothiazole-2-yl)-*N*’-ethylurea) were calculated by means of RB3LYP DFT calculations at the 6-31G(D) level with Spartan 08 software package. The structures of every possible isomer for each ligand were built and subjected to conformational searches at the molecular mechanics (MMFF force field) level. The retrieved conformations were then minimized with RB3LYP DFT calculations at the 6-31G(D) level, and the lowest energy configurations amongst all the possible outcomes were selected as the most stable forms for every compound.

We carried out ensemble docking studies as previously described, with the tubulin flexibility accounted for by the use of different complexes that sample the protein conformational space [16,47]. Briefly, we docked the compounds into the colchicine sites of 65 different tubulin structures from complexes of tubulin with colchicine site ligands deposited in the pdb, and 5 representative structures from a previous molecular dynamics simulation run on a tubulin–podophyllotoxin complex. We performed parallel docking studies using AutoDock 4.2 with the Lamarckian genetic algorithm (LGA) 100−300 times for a maximum of 2.5 million energy evaluations, 150 individuals, and a maximum of 27,000 generations, and used PLANTS with default settings and 10 runs per ligand. For ligands with amide bonds and urea groups, we used all the possible configurations in the docking runs, and selected the best scored one as the docking results for every ligand. We converted the scores of the different programs into Z-scores to allow for the comparison of the different scoring scales. We selected the common poses for the two programs with the best consensus Z-scores as the docking results. For every pose, we automatically assigned them to the colchicine subzones using in-house KNIME pipelines [48]. The RMSD between every pose and model scaffolds with no substituents and with the colchicine binding site ligands representative of the binders occupying different subzones were calculated using LigRMSD [49]. Docked poses were analyzed using Chimera [50], Marvin [51], OpenEye [52], and JADOPPT [53].

## 3. Results and Discussion

### 3.1. Chemistry and Properties

#### 3.1.1. Chemical Synthesis

**Scheme 1:**
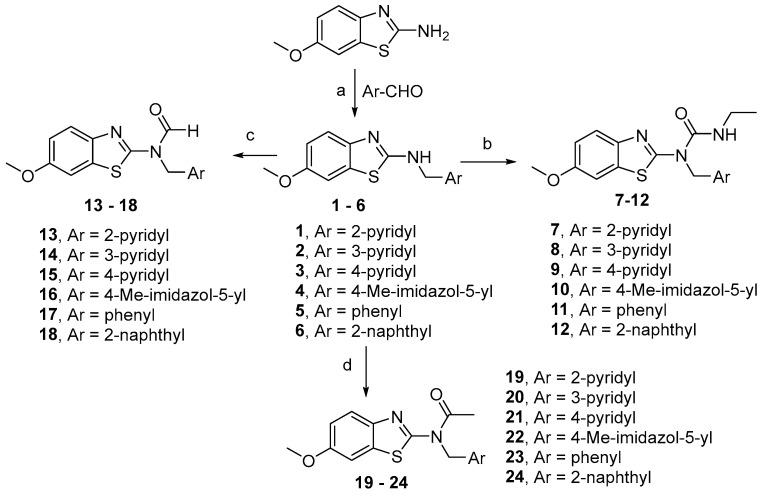
Synthesis of benzothiazole derivatives. Reagents and conditions: (a) 1. *p*TsOH, toluene, reflux, 16 h; 2. NaBH_4_, EtOH, 0 °C—rt, 2 h. (b) EtNCO, Et_3_N, DCM, 16 h, rt. (c) HCOOH, HOBt, EDC, DMAP, DCM, rt, 16 h. (d) Ac_2_O, DMAP, DCM, rt, 16 h.

The *N*-arylmethylbenzothiazole amines **1**–**6** were prepared via reductive amination in two steps. First, the imines were synthesized under acid catalysis (*p*-toluenesulphonic acid, *p*TsOH), and then sodium borohydride was used for the reduction in the intermediate imines. The structural modifications on the linker connecting the aromatic rings were accomplished via acylation of the secondary amino group of the benzothiazole derivatives, leading to the following three families of compounds: urea (**7**–**12**), formamide (**13**–**18**), and acetamide (**19**–**24**) derivatives (Scheme 1).

The transformations of the secondary amino groups into ethylurea to obtain compounds **7**–**12** were performed by the reaction between derivatives **1**–**6** and ethylisocyanate under basic conditions. The formamide derivatives **13**–**18** were obtained via the amide coupling reaction between the secondary amines of compounds **1**–**6** and formic acid, 1-ethyl-3-(3-dimethylaminopropyl)carbodiimide (EDC), 1-hidroxybenzotriazole (OHBt), and 4-dymethylaminopyridine (DMAP). The acetylation of the amines **1**–**6** with acetic anhydride afforded the acetamide derivatives **19**–**24** in a reaction catalyzed by DMAP. All the products were obtained in good or excellent yields. In the NMR spectra of the formamide derivatives, it was possible to distinguish the signals corresponding to both the *cis* and *trans* isomers. This can be explained by the energetic barrier to interconversion in the formamides that allows for the observation of both isomers in the NMR chemical shift timescale [54].

#### 3.1.2. Aqueous Solubility and Calculated Properties

One of the main drawbacks of the classical colchicine ligands is their low aqueous solubility. The high hydrophobicity of zones A and B in the colchicine site in the tubulin implies that the ligands with a high binding affinity, such as combretastatin A-4, exhibit non-drug-like physicochemical properties as a consequence of their hydrophobic structure. The development of moleculesadapted to interact with the more polar zone C is usually related to the design of polar ligands, such as the MI181, which binds zones A–C, or the sulfonamide ABT-751, which targets zones B–A–C. One of the goals of the design of 6-methoxybenzothiazol-2-amine derivatives prepared in this work is the substitution of the olefin bridge of combretastatins and MI-181 by amino, amido, or urea groups to improve the aqueous solubility. The thermodynamic water solubility of the synthesized compounds, in the phosphate buffer at pH 7.0, was determined using the shaking flask methodology. Combretastatin A4, MI-181, and ABT-751 were used as references, and the results are summarized in Table 1. The topological polar surface area (TPSA) and the logarithm of partition coefficient (Log P) were computationally calculated as descriptors related to water solubility, toxicity, and the ability of compounds to cross biological barriers (Table 1) [55].

The nature of the second aromatic ring of the benzothiazole derivatives was the main factor affecting the aqueous solubility. The presence of the naphthalene ring imposed low water solubilities below 9 µg/mL in all cases (e.g., compounds **6**, **12**, **18**, and **24**), similar to the less soluble reference compounds MI-181 and CA-4. The substitution of the large hydrophobic naphthalene ring by a phenyl moiety increased the solubility by about two-fold (e.g., compare compound **5** with **6**; **11** with **12**; **17** with **18**; and **23** with **24**) with water solubilities between 5 and 16 µg/mL for the phenyl derivatives. The replacement of the phenyl ring by the pyridine greatly increased the water solubility, ranging from 6- to 11-fold (e.g., compare compound **5** with **2**; **11** with **9**; **17** with **14**; and **23** with **20**), yielding values of 48–57 µg/mL for the aminomethyl-pyridine derivatives (**1**–**3**) and about 90–100 µg/mL for the amido- and urea-pyridine derivatives.

The position of the nitrogen of the 2-, 3-, or 4-pyridyl moieties did not make significant differences in the solubility values (e.g., compare compounds **13**, **14**, and **15**; or **7**, **8**, and **9**). The best solubility results were obtained with a 5-methyl-1*H*-imidazole moiety, with improvements between 1.5- and 2-fold (e.g., compare compound **3** with **4**, and **22** with **19**). The substitution on the amino group of benzothiazole derivatives was the second factor affecting the water solubility. Introducing an ethylurea group caused an increase in the water solubility of about 2-fold in comparison with the amino series. For the pyridine derivatives, the water solubility improved from around 50 µg/mL in the amino series to near 100 µg/mL in the urea family (e.g., compare compound **7** with **1**; **8** with **2**; or **9** with **3**). Similar trends were obtained for the substitutions of the amino groups by formamide (e.g., compare compound **13** with **1**; **14** with **2**; and **15** with **3**, showing improvements from 1.6- to 1.8-fold). The behavior of the acetamide derivatives was similar to their formamide analogs (e.g., compare compound **13** with **19**; **15** with **21**; and **16** with **22**). Therefore, the introduction of the ethylurea, formyl, or acetyl groups was favorable in terms of water solubility, which is probably explained by the higher topological polar surface area (TPSA) of the substituted derivatives with respect to the non-substituted ones (e.g., compare TPSA values of **1**, **7**, **13**, and **19**), as well as by the greater possibilities of the substituted derivatives to form hydrogen bonds with water. This last feature is the most relevant in the case of the urea derivatives having the highest solubility values (e.g., compare compound **7** with **13** or **19**; **8** with **14** or **20**; and **9** with **15** or **21**). Anywhere, all the synthesized compounds were more soluble than the references MI-181 and CA-4, and those with pyridine or imidazole rings showed higher solubilities than ABT-751. By comparing the structure of MI-181 with the 3-pyridyl derivatives (compounds **2**, **8**, **14**, and **20**), we can conclude that replacing the olefin with an amino, formamido, acetamido, or ethylurea group, together with the substitution of two methyl groups in positions 5 and 6 by a methoxy group in position 6 of the benzothiazole, results in large water solubility improvements.

The calculated TPSA values are consistent with the measured solubility since higher TPSA values were obtained for the more soluble molecules. The TPSA correlates well with the passive molecular transport through membranes, and it is also a good predictor of non-specific toxicity, since below the threshold of 75 Å^2^, the molecules are suspected to display unspecific cytotoxicity [55]. For all the pyridine and imidazole derivatives, the calculated TPSAs were over 75 Å^2^, with values ranging from 91 to 111 Å^2^ for the latter; 83–95 Å^2^ for the pyridine derivatives with urea, formamide, and acetamide groups; and 75 Å^2^ for the rest of the pyridine derivatives (compounds **1**, **2**, and **3**). The phenyl and naphthyl derivatives not only showed the lowest water solubility, but also unfavorable TPSA values below 70 Å^2^. 

The partition coefficient between n-octanol and water (Log P*_o_*_/*w*_) is the classical descriptor for lipophilicity. A good cell permeability is associated with LogP*_o_*_/*w*_ values between two and three [55]. The calculations predicted LogP*_o_*_/*w*_ values within that range for most of the soluble compounds. The only exception is compound **7**, with a higher LogP*_o_*_/*w*_ than its analogs, which is consistent with the possible formation of an intramolecular hydrogen bond between the endocyclic nitrogen of pyridine and the urea group.

### 3.2. Biology

#### 3.2.1. Antiproliferative Activity

The MTT method was used to measure the effect of the synthesized compounds **1**–**24** on the in vitro cell viability 72 h after drug exposure. The compounds were tested against a panel of ten different cell lines. Eight of them were human tumor cell lines representative of some of the cancers with the highest incidence worldwide, including HeLa cervix epithelioid carcinoma, MCF7 breast adenocarcinoma, U87MG glioblastoma, T98G temozolomide resistant glioblastoma, HepG2 hepatocellular carcinoma, HT-29 colon adenocarcinoma, and LNCaP and PC-3 prostate cancer. LNCaP is a hormone-sensitive cell line and represents an early stage of prostate cancer, whereas PC-3 is more resistant to chemotherapy and is a model for hormone-refractory prostate cancer. The compounds were also tested against the GL261 mice glioblastoma cell line that is usually studied previously to glioblastoma xenograft experiments in mice, and against the non-tumorigenic HEK-293 cell line from human embryonic kidney tissue to evaluate the selectivity of compounds between healthy cells and tumor cells. The compounds inhibiting cell proliferation by at least 40%, with respect to the negative control at 10 µM, were evaluated at concentrations in the range between 0.1 nM and 20 µM, and the half-maximal inhibitory concentrations (IC_50_) were calculated (Table 2). ABT-751, docetaxel (DTX), and temozolomide (TMZ) were used for comparative purposes since the first one is an example of an antimitotic agent binding at the colchicine site with moderately good water solubility, DTX is a tubulin-binding agent in clinical use, and TMZ is the only approved treatment for glioblastoma. The results are summarized in Table 2.

With regard to the effect of the other aromatic rings of the benzothiazole derivatives on antiproliferative activity, the pyridyl derivatives were the best-performing compounds within each series. The phenyl analogs were less potent than the equivalent pyridines, with most of them showing IC_50_ values in the low µM range. The imidazoles were all inactive, as well as the naphthalenes, with the only exception being the unsubstituted naphthyl methylamine **6**, with antiproliferative values in the low µM range.

The pyridines exerted antiproliferative activities in all tumor cell lines with IC_50_ va-lues ranging from micromolar to low nanomolar depending on the substituent on the amino group and the cell line. The pyridyl derivatives with an unsubstituted amino group (compounds **1**, **2**, and **3**) showed potencies in the one-digit micromolar range, circa 1 µM for HeLa, HepG2, MCF7, and U87MG, and between 2 and 3 µM for the rest of the tumor cell lines. Some differences were found for the different isomers of the pyridine analogs since the 3-pyridyl derivatives showed, in general, a lower IC_50_ than the 4-pyridyl isomers, which, in turn, showed higher potencies than the 2-pyridyl derivatives (e.g., compare compound **1**, **2**, and **3**; **7**, **8**, and **9**; **13**, **14**, and **15**; or **19**, **20**, and **21**).

For the pyridyl derivatives, the substitutions on the amino group greatly improved the antiproliferative potencies, particularly in the ethylurea (**7**, **8**, and **9**) and formamide series (**13**, **14**, and **15**), whereas a smaller improvement was observed in the acetylated analogs (**19**, **20**, and **21**). The functionalization with the ethylurea group decreased the IC_50_ values from micromolar to submicromolar or nanomolar in most cell lines (compare **1**, **2**, and **3** with **7**, **8**, and **9**, respectively), reaching higher potencies than the reference ABT-751 in all the cell lines. In the case of the 3-pyridyl derivatives, the changes were higher when moving from the amino (**2**) to the urea (**8**) derivative, resulting in double-digit nanomolar IC_50_ values for compound **8**, ranging from 25 to 94 nM in HeLa, HepG2, MCF7, T98G, GL261, and LNCaP, and rising to submicromolar values only for the more resistant cell lines PC-3 (161 nM) and HT-29 (208 nM). Interestingly, compound **8** was especially potent to inhibit cell proliferation in the glioblastoma cell lines, with an IC_50_ value of 9 nM in U87MG. For the less sensitive glioblastoma cell line T98G, the IC_50_ value was 52 nM, and 25 nM for GL261. These results are especially relevant, considering that the IC_50_ of the only approved drug for the treatment of glioblastoma, TMZ, is over 100 µM in these glioblastoma cell lines. Compound **8** also showed lower IC_50_ values than DTX against the U87 MG, T98G, GL261, and HT-29 cells. The ethylurea-pyridyl derivatives in compounds **7** and **9** also displayed lower IC_50_ values than their non-substituted counterparts in compounds **1** and **3**, respectively, showing between 6- and 20-fold decreases in the IC_50_ values with potencies lower or similar to ABT-751. Their behavior was similar to that observed for compound **8** in the sense that higher potencies were obtained for the glioblastoma cell lines (double-digit nanomolar range in U87MG and GL261) compared with the rest of the cell lines (IC_50_ values between 0.1 and 0.3 µM). However, in all the cell lines, compound **8** performed better than its analogues **7** and **9**. The higher sensitivity of the glioblastoma cell lines is also remarkable when compared to the HeLa cells, which are usually highly sensitive to microtubule inhibitors [56]. These results suggest that the glioblastoma cells have a special sensitivity to this class of microtubule drugs. Besides the high sensitivity of the glioblastoma cell lines, the breast cancer cell line MCF7 also shows a higher sensitivity to these compounds, which is in agreement with the previous results for the colchicine site tubulin inhibitors [57,58].

The amine formylation also increased the antiproliferative potencies (compare compounds **13**, **14**, and **15** with **1**, **2**, and **3**, respectively), shifting the IC_50_ values from the micromolar range for the unsubstituted compounds **1**–**3** to at least the submicromolar range for the formamide derivatives in compounds **13**–**15**, thus performing better than or similar to the reference ABT-751. As observed in the ethylurea series, the 3-pyridyl isomer in compound **14** was also the most potent in all the cell lines, demonstrating a particularly strong effect not only in the glioblastoma cell lines, reaching IC_50_ between 37 and 75 nM, but also in the MCF7 cells (15 nM). Similar trends were observed for the 2- and 4-pyridyl analogues (compounds **13** and **15**), with submicromolar IC_50_ values in all the cell lines (0.3–0.5 µM) and slightly lower values in the glioblastoma and MCF7 cells (near 0.2 µM).

The amine acetylation enhanced the IC_50_ values with respect to the non-substituted analogs (e.g., compare compounds **1**, **2**, and **3** with **19**, **20**, and **21**, respectively), but the effect was less pronounced than in the ethylurea or formamide series, since the improvements did not change the order or magnitude of the IC_50_ values in most cases. The 3-pyridyl derivative of the acetamide series (compound **20**) was again the most potent compound of the family, reaching submicromolar IC_50_ values against the HepG2, MCF7, U87MG, T98G, and GL261 cells. The superiority of the 3-pyridyl isomers (when the amino group is substituted) in the glioblastoma cells was also maintained in this family. The 2- and 4-pyridyl analogs (compounds **19** and **21**) led to little improvements with respect to their non-substituted counterparts (compounds **1** and **3**, respectively), with IC_50_ values in the low micromolar range.

Regarding the nature of the linker connecting the aromatic rings, the phenyl and pyridyl derivatives behave differently from the naphthalenes and imidazoles. For the first group, the amine acylation always results in potency improvements, while for the second one, acylation is detrimental. This suggests a size, shape, and probably polarity origin for the observed different behavior. For the six-membered rings, a higher effect is observed for the pyridines, which suggests the implication of a favorable polar interaction of the target with the azine nitrogen. The maximal effect within the pyridines is obtained for the 3-pyridyl isomers, with a geometry dependency that is also consistent with a polar directional interaction with the target that is optimal for the nitrogen in that position. The lower improvement observed for the 2- and 3-pyridyl isomers suggest a less optimal disposition for the directional bond, while the lower enhancement observed for the phenyl analogs is also consistent with the loss of such a favorable interaction. 

All the compounds were also tested against the non-tumorigenic HEK-293 cell line, and all of them showed lower potencies than when they were tested against the tumor cell lines. Most of the compounds displayed no cytotoxic effect below 10 µM (compounds **1**–**6**, **10**–**12**, **16**–**18**, **19**, and **21**–**24**). Interestingly, the most potent antiproliferative compounds against the tumor cells (the pyridyl derivatives in compounds **7**–**15**, **13**–**15**, and **20**) showed a low cytotoxicity toward the HEK-293 cells, with IC_50_ values between 8 and 10 µM and selectivity indexes between 25 and 100 for most of the cell lines, displaying selectivity indexes near 900 and 500 for the best-performing compounds (compound **8** against U87MG and compound **14** against MCF7, respectively). The selectivity indexes are much better than those for the reference compound ABT-751 and DTX, which had selectivity indexes between three and six for the tumor cells. These results suggest that the new benzothiazole derivatives have a significant safety profile for their use as antitumor agents.

#### 3.2.2. Sensitivity to MDR Efflux Pumps

The compounds exhibited higher IC_50_ values against the HT-29 cells compared with the rest of the cell lines, which agrees with the previously reported data for the colchicine site ligands. The low sensitivity of the HT-29 cells to the tubulin polymerization inhibitors were related to autophagy [59], activity blockage by UDP-glucuronidation [60], or multidrug resistance mechanisms (MDR efflux pump-like P glycoprotein, P-gp) [61]. The compounds lack functional groups that are compatible with glucuronidation reactions. Since an acquired resistance to antitumor drugs is one of the more important limitations of chemotherapy, we studied the possibility that these compounds are substrates of MDR efflux pumps. For that purpose, the IC_50_ values of the benzothiazole derivatives against the HT-29 cells pre-treated with verapamil, a P-glycoprotein 1 and multidrug resistance protein 1 inhibitor (MDR1), at 10 µM, which is a concentration that does not affect cell proliferation, were compared with the values in absence of the inhibitor. If a compound is susceptible to MDR efflux, its IC_50_ values will be significatively lower in the presence of verapamil. No differences were observed between the two experiments with or without verapamil (Table 3), as well as the reference compound ABT-751, thus suggesting that they are not MDR substrates.

#### 3.2.3. Tubulin Polymerization Inhibition (TPI)

To determine whether the tubulin polymerization inhibition (TPI) is the mechanism of action responsible for the antiproliferative effect of the active compounds, their effect on the in vitro assembly of bovine brain tubulin was studied. The degree of tubulin polymerization in the presence or absence (negative control) of the compounds was determined via turbidimetry. All the compounds were assayed at a concentration of 10 µM, and for those inhibiting tubulin polymerization by more than 50% compared to the untreated control, the IC_50_ values were determined. 

The TPI and the antiproliferative activity were strongly correlated, since compounds with TPI IC_50_ values lower than 5 µM showed IC_50_ values in the submicromolar range, and the non-cytotoxic compounds were not able to inhibit tubulin polymerization at 10 µM (Table 4). This is in agreement with tubulin inhibition being the mechanism of the antiproliferative effect. Additionally, this lack of TPI activity for the non-antiproliferative compounds is due to a lack of activity on tubulin and not to a reduced uptake, which is in good agreement with the antiproliferative and verapamil cotreatment experiments.

The pyridine derivatives behaved as the most potent inhibitors, following the same trends as in the antiproliferative activity. The pyridine derivatives with no substituents on the amino group (**1**, **2**, and **3**), showing micromolar antiproliferative activities, inhibited tubulin polymerization below 50% at 10 µM, whereas the pyridine derivatives with ethylurea (**7**, **8**, and **9**) or formamide functionalities (**13**, **14**, and **15**), whose antiproliferative IC_50_ values where submicromolar or nanomolar, exhibited TPI IC_50_ values between 2.9 and 5.0 µM, which is similar to or better than the reference compounds ABT-751 (4.4 µM), MI-181 (4.6 µM), and CA4 (2.8 µM). The acetylation of the bridge amino group did not greatly improve the TPI (e.g., compare **19**, **20**, and **21** with **1**, **2**, and **3**, respectively), nor did it decrease the antiproliferative IC_50_ by orders of magnitude. The best-performing ligands as tubulin polymerization inhibitors were again the most cytotoxic, namely, the 3-pyridyl derivatives in all series (compounds **2**, **8**, **14**, and **20**), and the most potent of them (compound **8**) showed a TPI IC_50_ value below that of the ABT-751 (2.9 vs. 4.4 µM). In the acetyl series, the 3-pyridyl derivative (**20**) was the only ligand with a TPI IC_50_ value below 10 µM, and it was also the only compound in that series with submicromolar antiproliferative activity. This correlation strongly suggests that tubulin inhibition is behind the observed antiproliferative effects. The differences in the TPI values for the pyridine isomers also parallel the observed differences in the antiproliferative potency, which is in good agreement with the ranking order due to different interactions with the target and is consistent with the proposed directional polar interaction of the pyridine nitrogen with tubulin. Again, the potency drops in the TPI for the phenyl analogs; the putative lack of this interaction further supports this argument. 

The naphthyl and imidazolyl derivatives (**4**, **6**, **10**, **12**, **16**, **18**, **22**, and **24**), the less potent cytotoxic compounds, were not able to inhibit tubulin polymerization at 10 µM regardless of the substituents on the amino group, thus confirming that the replacement of pyridine by 5-methyl-imidazole or naphthalene hinders the interaction with tubulin, probably because an imidazole ring is too small and/or too polar to fit in the tubulin pocket, whereas the naphthalene moiety is too hydrophobic and/or too large. Even though compound **6** showed antiproliferative activity in the micromolar range in some cell lines, it failed to inhibit tubulin polymerization at 10 µM. The differences in the order of magnitude between the two experiments can be explained by the fact that the observation of the antiproliferative effect only needs the alteration of the tubulin polymerization dynamics at a low drug concentration, whereas the polymer mass changes at high protein and compound (micromolar) concentrations, which are required in TPI assays.

#### 3.2.4. Effects on Cellular Microtubules

**Figure 3:**
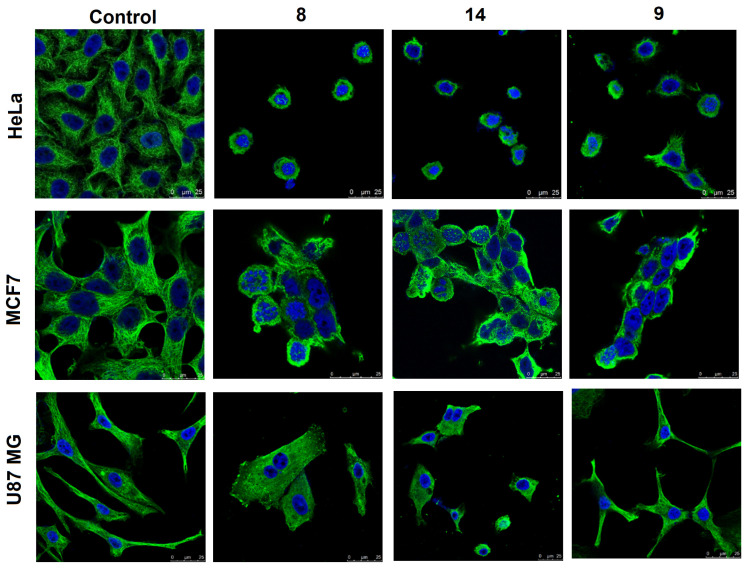
Effects of the treatment with compounds **8**, **14**, and **9** on the microtubule network in HeLa, MCF7, and U87MG cells as observed via confocal microscopy 24 h after incubations. For immunofluorescence labeling, α-tubulin was stained in green, and nuclei were stained in blue. Different concentrations were used according to the different antiproliferative IC_50_ values of compounds in HeLa (200 nM for compounds **8** and **14**, and 500 nM for compound **9**), MCF7 (100 nM for compounds **8** and **14**, and 500 nM for compound **9**), and U87MG cells (50 nM for compound **8**, and 100 nM for compounds **9** and **14**). Scale bar: 25 µm.

To confirm that the antiproliferative activity of the compounds in tumor cells is caused by the interference with tubulin polymerization, the effects of the representative compounds on cellular microtubules were investigated. For that purpose, we selected three of the most potent pyridyl derivatives with a substituent on the amino group, which are the ethylurea derivatives **8** (3-pyridyl derivative) and **9** (4-pyridyl derivative), and the 3-pyridyl formamide **14**. We studied the effects of the three compounds on the microtubule networks of the HeLa, MCF7, and U87MG cells 24 h after the treatment via immunofluorescence confocal microscopy, labeling α-tubulin and the nuclei in comparison with the untreated cells (Figure 3).

In the negative controls, for every kind of tumor cell, a characteristic morphology with hairy microtubule filaments, in green, covering all the cytoplasm around the nucleus, in blue, is observed. In the samples treated with compounds **8** and **14**, besides a decrease in the cell numbers due to the antiproliferative effect, a disruption of the microtubule network can be observed in the three cell lines. After treatment with compounds **8** and **14**, the HeLa and MCF7 cells acquired a rounded shape, whereas the U87MG cells lost their typical trigonal shape and adopted broader polygonal shapes. In the three cases, no microtubule fibers except for a green diffuse mass with no defined structure was observed in the treated cells. This loss of microtubules and the cell morphology changes are in good agreement with the compounds affecting tubulin. Compound **9** caused similar effects in the microtubule in the MCF7 and HeLa cells. It also caused a microtubule network disruption in the U87MG cells, but to a lesser extent than compounds **8** and **14** since some tubulin fibers remained, and the cell shapes were not affected. The MCF7 cells treated with compounds **8** and **14** exhibited multilobulated nuclei, thus supporting the argument that the mitotic arrest caused by the impairment of the microtubule assembly is the mechanism at play [57].

#### 3.2.5. Effects on the Cell Cycle

The effect of the selected compounds **8**, **9**, and **14** on the cell cycle distribution in the HeLa, MCF7, and U87MG cancer cell lines was studied via flow cytometry at different time points after the treatments (24, 48, and 72 h). The time-course analyses of the cell cycle showed different evolution depending on the compound and the cell line.

**Figure 4:**
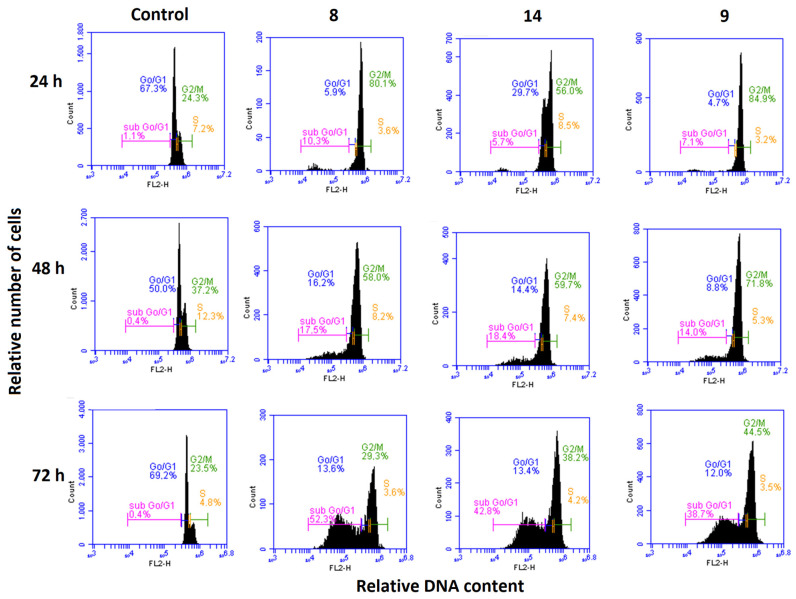
Histograms of the cell cycle of HeLa cells after 24, 48, or 72 h of treatment with compounds **8** (200 nM), **14** (200 nM), or **9** (500 nM). Untreated control cells were run in parallel. The positions of the subG0/G1 (pink), G0/G1 (blue), S (orange), and G2/M (green) peaks are indicated by bars of different colors. The histograms are representative of three independent experiments.

The non-treated HeLa cells (negative controls) showed similar cell cycle profiles regardless of the time point, with most of the cell population in the G0/G1 phases (50–69%), 24–37% of cells in the G2/M, and only 0.4–1.1% of cells at the subGo/G1 region (Figure 4). High percentages of cells were arrested at G2/M (80, 56, and 85% for compounds **8**, **14**, and **9**, respectively) 24 h after the treatments. Besides these differences, 48 h after incubation, the mitotic arrest was similar for the three compounds (58, 60, and 72% for compounds **8**, **14**, and **9**, respectively), and the subG0/G1 populations reached 14–18%. At a later time point of 72 h, the G2/M population decreased at the expense of an accumulation of sub G0/G1 fractions, which is indicative of cell death; this consisted of 52, 43, and 39% of cells in the subG0/G1 region for compounds **8**, **14**, and **9**, respectively. This reveals that the antiproliferative effect of the three compounds on the HeLa cells is due to a mitotic arrest that begins only 24 h after the treatment and is followed by cell death induction. 

**Figure 5:**
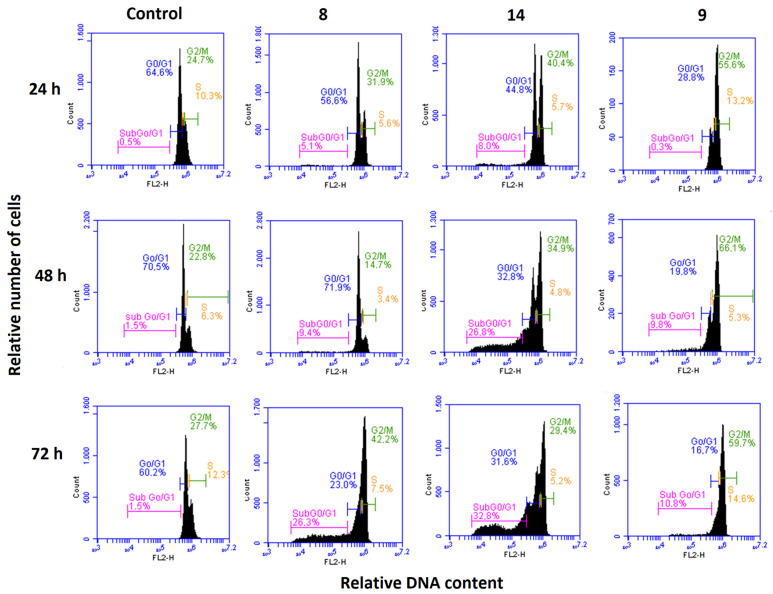
Histograms of the cell cycle of MCF7 cells after 24, 48, or 72 h of treatment with compounds **8** (100 nM), **14** (100 nM), or **9** (500 nM). Untreated control cells were run in parallel. The positions of the subG0/G1 (pink), G0/G1 (blue), S (orange), and G2/M (green) peaks are indicated by bars of different colors. The histograms are representative of three independent experiments.

The cell cycle profiles of the untreated MCF7 cells (negative controls) at 24, 48, and 72 h were similar to the HeLa profiles (Figure 5). However, the effects of the selected compounds on this cell line were different than on the HeLa cells. The three compounds triggered mitotic arrest 24 h after the treatments (32, 40, and 56% for compounds **8**, **14**, and **9**, respectively). Compound **9** was able to maintain the cell populations at G2/M, but the subG0/G1 fraction only reached 11% 72 h after the treatment, thus indicating that compound **9** disrupts mitosis in the MCF7 cells, but the cell death induction is not so strong. The profiles for compounds **8** and **14** evolved differently. The mitotic arrest caused 24 h after the treatment by compound **14**, the most potent against the MCF7 cells, slowly declined at 48 and 72 h (30–35%), accompanied by an accumulation of 27% of the cells at subG0/G1 48 h after incubation that increased to 33% at the final time point of 72 h. The mitotic arrest caused by compound **8** had a slow start as well as the cell death induction; 48 h and 72 h after incubation, the subGo/G1 population changed from 9 to 26%. The MCF7 cells are deficient in caspase-3, which can explain the difficulties of the compounds in inducing cell death compared to the HeLa cells [62].

**Figure 6:**
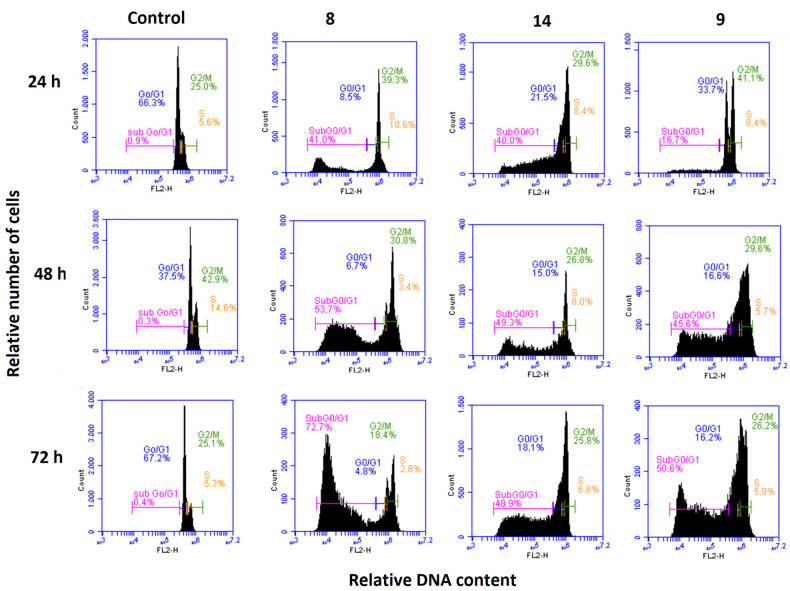
Histograms of the cell cycle of U87MG cells after 24, 48, or 72 h of treatment with compounds **8** (50 nM), **14** (200 nM), or **9** (200 nM). Untreated control cells were run in parallel. The positions of the subG0/G1 (pink), G0/G1 (blue), S (orange), and G2/M (green) peaks are indicated by bars of different colors. The histograms are representative of three independent experiments.

The untreated U87MG cells displayed similar cell cycle histograms to those of the HeLa and MCF7 cells at 24, 48, and 72 h (Figure 6). The most potent compound **8** caused a severe mitotic arrest (39% in G2/M) and cell death induction (41% in subG0/G1) only 24 h after the treatment, followed by a progressive increase in the latter at the expense of the former over time, with the combined populations staying roughly constant at 85%. Compound **14** showed a maintained G2/M arrest and cell death induction at the three time points, staying globally at roughly 75%. Compound **9**, after a slower start with a global G2/M (41%) plus subG0/G1 (17%) of 58%, also stabilized at later time points at 75%, again with the increased contribution of the apoptotic subG0/G1 population (46% at 48 h and 51% at 72 h) at the expense of the G2/M population (30% at 48 h and 26% at 72 h) observed for the other two compounds. This could explain the immunofluorescence results (observed 24 h after the treatments), where the U87MG cells did not appear to be too affected by compound **9** in comparison with the cells treated with compounds **8** and **14**.

In summary, although different cell cycle profiles were registered, the three compounds, **8**, **9**, and **14**, were able to arrest the cell cycle at G2/M in the HeLa, MCF7, and U87MG cells 24 h after the treatments. Then, a certain level of mitotic arrest was maintained for 48 and 72 h while the SubG0/G1 population gradually increased until it reached maximum values at the 72 h time point, which is in good agreement with the antiproliferative IC_50_ measurements. 

#### 3.2.6. Cell Death Mechanistic Studies

**Figure 7:**
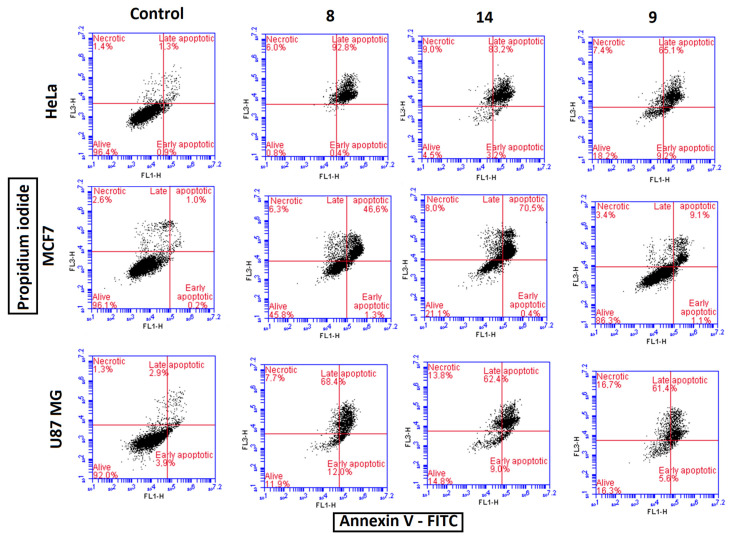
Annexin V-FITC and propidium iodide (PI) profiles in HeLa, MCF7, and U87MG cells 72 h after treatment with compounds **8**, **14**, or **9**. Different concentrations were used according to the different antiproliferative IC_50_ values of the compounds in HeLa (200 nM for compounds **8** and **14** and 500 nM for compound **9**), MCF7 (100 nM for compounds **8** and **14** and 500 nM for compound **9**) and U87MG cells (50 nM for compound **8** and 100 nM for compounds **9** and **14**). Untreated cells were used as negative controls.

To elucidate the cell death mechanism suggested by the accumulation in the subGo/G1 region 72 h after the treatments with compounds **8**, **9**, and **14**, we studied the mechanism of cell death via dual channel flow cytometry experiments using double staining by fluorescein isothiocyanate-labeled Annexin V (AnV) and propidium iodide (PI). The HeLa, MCF7, or U87MG cells were incubated with compounds **8**, **9**, or **14** for 72 h, stained with AnV and PI, and analyzed via flow cytometry (Figure 7). The untreated cells were used as negative controls. The apoptotic cells are Annexin V positive due to the translocation of phosphatidylserine from the inner leaflet to the surface of the plasmatic membrane, whereas necrosis causes membrane disintegration, leading to PI permeation. The response of the cells to both stains allow us to determine if they are viable (PI−, AnV−), early apoptotic (PI−, AnV+), late apoptotic, secondary necrotic (PI+, AnV+), or only necrotic (PI+, AnV−). The untreated (negative control) HeLa, MCF7, and U87MG cells showed live cell populations of over 92%. 

In agreement with the cell cycle results, most of the treated HeLa cells suffered from apoptotic induction 72 h after the treatments, and the three compounds had similar behaviors. A total of 93% of the HeLa cells treated with the most potent compound **8** underwent early and late apoptosis, whereas nearly 75% of apoptosis was observed for the less potent compound **9**, and 86% of apoptotic cells were caused by compound **14** (Figure 7).

The percentages of the early and late apoptotic cells were higher than the fractions of the cells in the subG0/G1 phase, which can be explained by the different techniques employed in each assay. For the cell cycle experiments, a single PI staining after permeabilization is used. This means that many of the cells whose DNA content corresponds to the G2/M or even G0/G1 phases are probably beginning the phosphatidylserine translocation, but since their DNA is not yet affected, PI staining does not discriminate apoptotic cells. When double PI/AnV staining is performed, the apoptotic cells that were included in the G2/M or G1/G0 fractions in the cell cycle analysis respond positively not only to PI, but also to Annexin V.

Regarding the MCF7 cells (Figure 7), only compound **14** was able to induce apoptosis to a similar degree as observed for the HeLa cells (71%). Compound **8** also caused cell apoptosis, but to a lesser extent (47%), showing 46% of alive cells, and compound **9** displayed a very similar profile to the control cells since only 12% of cells underwent apoptosis. These results are consistent with the cell cycle profiles whose analyses revealed that mitotic arrest in MCF7 is not always followed by cell death. This behavior of the MCF7 cells can be attributed to their deficiency in caspase-3, which plays an essential role in the signal cascade of apoptosis.

Higher levels of apoptosis were registered for the treated U87MG cells compared with the MCF7 cells, but for the three treatments, 12–16% of cells were still alive at 72 h. As expected, a strong apoptotic response was observed after the treatment with the most potent compound **8**, showing most cells in early or late apoptosis (80%). A lower response to Annexin V was displayed by the cells treated with compounds **9** and **14** (71% and 67%, respectively), showing similar profiles, which is in agreement with their similar antiproliferative potencies.

For the HeLa and MCF7 cells, the percentage of alive cells after 72 h of treatment correlates with the antiproliferative IC_50_ values. On the other hand, the percentage of alive U87MG cells remains at a roughly constant value irrespective of the potency of the compounds.

#### 3.2.7. Lactate Dehydrogenase Assays

An analysis of treated cells via flow cytometry after simple (PI) or double (PI/AnV) staining, together with the immunofluorescence experiments, revealed that the antiproliferative effects of the selected compounds through MTT assays are explained by the induction of mitotic arrest, which occurs 24 h after incubation, which leads to apoptotic cell death 72 h later. All these experiments are complementary and accounted for different aspects of the cell damage caused by the compounds. The LDH leakage assay measures the release of lactate dehydrogenase (LDH) into the culture medium after cell membrane damage. It is a very interesting experiment because it can detect low levels of cell membrane damage that are not detected by other methodologies. The selected compounds **8**, **14**, and **9** showed to be particularly potent against glioblastoma cell lines. This is an important issue, considering that the only approved drug for glioblastoma treatment, temozolomide, has a very low antitumoral effect and is associated with resistance mechanisms.

**Figure 8:**
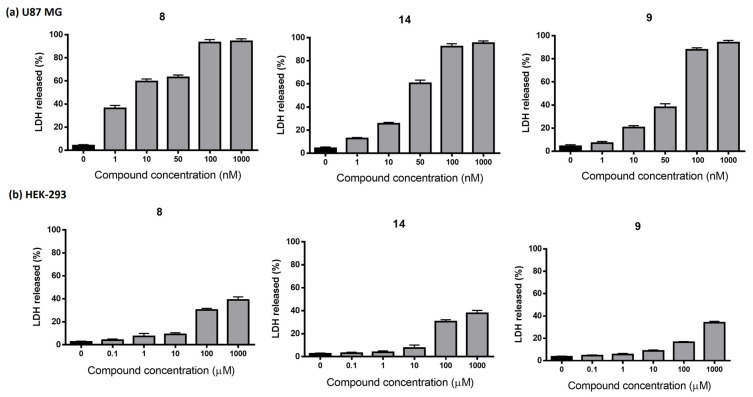
Dose–response experiments of LDH assays on U87MG (**a**) and non-tumoral HEK-293 (**b**) cells after 72 h of incubations with compounds **8**, **9**, or **14**. Concentrations are expressed in different units for each cell line. Untreated cells were used as negative controls. LDH release is defined by the ratio of LDH released/total LDH present in the cells, with the total LDH being 100%. The results are expressed as the mean of experiments run in quadruplicate.

To further study the effects caused by the compounds in glioblastoma cells and their capacity to discriminate between tumor and healthy cells, LDH experiments were carried out in both the glioblastoma U87MG cells and non-tumorigenic HEK-293 cells. The U87MG cells were treated for 72 h with concentrations of compounds from 1 nM to 1 µM, whereas the HEK-293 cells were incubated at higher concentrations, from 0.1 µM to 1 mM. These experiments revealed that the selected compounds caused further cellular damage than what was inferred by previous experiments, even at low nanomolar concentrations (Figure 8). An amount of 1 nM of compound **8** caused nearly 40% of LDH release in the U87MG cells, which was increased to over 60% at 10 nM and reached 100% at 100 nM, meaning that total cell death is achieved at that concentration. Compounds **9** and **14** also provoked 100% of cell death above 100 nM. Although the antiproliferative IC_50_ values of compounds **9** and **14** were in the double-digit nanomolar range, considerable amounts of released LDH, around 10% and 20%, were registered at concentrations as low as 1 and 10 nM, respectively, for both compounds, thus demonstrating a strong capacity to damage tumor cells. The LDH release values are consistent with the antiproliferative values obtained using the MTT method.

Consistent with the antiproliferative assays, the HEK-293 cells were less sensitive to the selected compounds than the U87MG cells. No significant cytotoxic effects were observed for the HEK-293 cells 72 h after the treatment with compound **14** in a concentration range between 0.1 and 1 µM, whereas the three compounds displayed roughly 10% of LDH release at 10 µM. It is important to note that the three compounds reached their maximum cytotoxic effect in the U87MG cells at 100 nM and, for that concentration, no significant cell damage was detected by the LDH assays. At a higher concentration of 100 µM, the LDH release was only 20% for compound **9**, and nearly 30% for compounds **8** and **14**. Interestingly, the maximum levels of cytotoxicity were not above 40% at concentrations as high as 1 mM. These results confirm the high selectivity of the compounds, with the ability to cause a higher cytotoxic effect in tumor cells compared with healthy cells.

### 3.3. Computational Studies

#### 3.3.1. Structural Effects of the Bridge Modifications

The DFT calculations show that the amines preferentially adopt an extended conformation. On the other hand, the preferred dispositions for the acylated analogs correspond to a folded arrangement that places the aromatic moieties in a close, non-planar disposition similar to that observed for Z-stilbenes, such as the active configuration of combretastatin A-4 and most ligands occupying sites A and B of the colchicine site [63]. These dispositions are those found as the preferred docked poses (see later), thus suggesting that it has an important role in the different observed binding modes. Furthermore, the pre-arrangement of the free ligands in the bound disposition should favor binding and result in a potency increase. 

#### 3.3.2. Docking Studies

**Figure 9:**
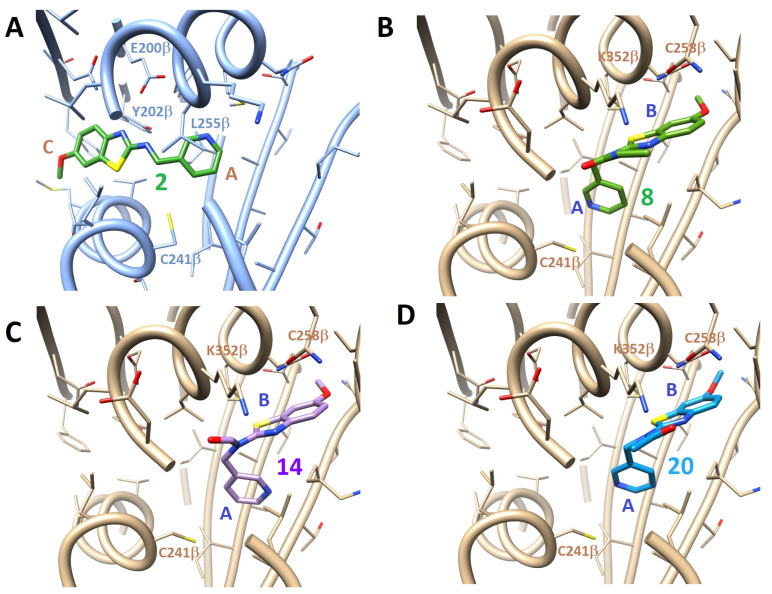
Docking poses for compounds **2** (panel (**A**)), **8** (panel (**B**)), **14** (panel (**C**)), and **20** (panel (**D**)) in complex with tubulin (5YL4 in blue, panel (**A**), and 5H7O in sepia, panels (**B**–**D**)). The colchicine site zones are indicated by letters, along with interacting sidechains.

The binding modes of the synthesized compounds (Figure 9) at the colchicine site of the tubulin were studied via ensemble docking experiments. X-ray crystal structures of the colchicine site ligands bound to the tubulin have shown that it can bind compounds of varied sizes and nature, more than what was initially thought, occupying different sub-pockets even for structurally related ligands [63]. Therefore, we accounted for the colchicine site flexibility by using in the docking experiments an ensemble of X-ray structures of tubulin in complexes with a set of diverse colchicine site ligands that represent different protein arrangements and ligand pharmacophore configurations, and therefore, efficiently explored the possible interaction space in the docking experiments [64]. We selected 60 X-ray crystal structures [65], plus 6 additional ones from a previous molecular dynamics simulation [66] obtained as described, to represent the different possible configurations of the colchicine site of the tubulin. 

Two docking programs with very different scoring functions (PLANTS [67] and AutoDock 4.2 [68]) were used for the docking experiments. For every docked ligand, we took the binding pose as the common pose that was the best scoring combination for the two docking programs as assessed via automated geometry and scoring comparison procedures. The geometry assessment consisted of the following: (a) sub-pocket occupancy assignments for each ligand pose calculated by measuring the lowest distances to the sub-pocket geometrical centers as determined by the pharmacophores derived from the X-ray structures of the colchicine site ligands in complex with tubulin; (b) RMSD calculations between the poses and unsubstituted scaffolds with the same bridges that place the phenyl rings at the centers of the A, B, or C sites of the colchicine site of the tubulin; and (c) the RMSDs of the poses with those of the pdb [69] X-ray structures of combretastatin A-4 (for A-B binding), MI-181 (for A-C binding), or ABT-751 (for A-B-C binding) (pdb IDs 5LYJ, 4YJ2, and 3HKC, respectively). Accordingly, every pose for every ligand is allocated to the corresponding sub-pockets. To make the inter program scores comparable, the individual scores were converted to relative scales where they rank between zero (worse) and one (best) and to Z-scores. Subsequently, we assigned a consensus binding mode for each ligand to the pair of poses common to the two programs with the best possible *Z*-values for each. The control experiments with representative colchicine site ligands of the known X-ray structures in complex with tubulin correctly retrieved the same experimental poses as before [16].

Amines **1** to **6** bind at the A (placed above sheets S9, S8, and S10 along the A to C direction and below helix H8) and C (placed with sheets S5, S6, and S7 below and helix H7 above) zones of the colchicine site of the tubulin (Figure 9A) [17]. The methoxy benzothiazole amines bind with the methoxy phenyl ring deepest in the β subunit occupying a hydrophobic pocket surrounded by the sidechains of Phe20β (H1), Phe169β (S5), Met235β (H7), the thiazole nitrogen, and the amine contacting Glu200β (S6) and Tyr202β (S6) at the C zone, and with the other aromatic rings occupying the A pocket, above the sidechains of Ile378β (S10), Ala316β (S8), and Ile318β (S8) and behind Leu255β (H8). 

The acylation of the methoxy benzothiazole amines **1**–**6** to formamides **13**–**18**, acetamides **19**–**24**, and ethylureas **7**–**12** results in an alternative binding mode at the B and A zones (Figure 9C,D). This change is due to the steric hindrance of the acyl moieties and the induction of a preference for a “folded” arrangement of the two aryl systems, a disposition required for the binding to the A–B zone, and a well-documented requirement for related systems. In these series, the methoxy-benzothiazole occupies the B zone, stacked behind helix H8 and with the sidechain of Asn258β (H8) piled above the phenyl ring that makes hydrophobic contacts with Met259β (H8), Thr314β (S8), and the methylenes of Lys352β (S9). The acyl moieties protrude toward the interfacial space between the subunits, and the other aromatic moieties occupy the A zone, filling from above the pocket formed by the sidechains of Ala316β (S8), Ile318β (S9), Lys352β (S9), and Ala354β (S9), capped by Ala250β (H7), Leu255β (H8), and with the pyridine ring orienting its nitrogen atoms toward the thiol group of Cys241β (H7). The optimal arrangement of the pyridine nitrogen is observed for the 3-pyridyl analogs, which is in good agreement with the biological results.

The ensemble docking approach used not only selects the most favorable poses for each ligand, but also provides information about the preferred protein structures that give these poses. In this respect, the amine derivatives **1**–**6** binding at zones A–C are selected for the protein structures that have ligands in these same sub-pockets. The most frequent pdb ID retrieved is 5YL4, whose ligand is a benzophenone analog of plinabulin (Figure S25 in the Supplementary Material) that deeply inserts the benzophenone moiety in the subunit. For the acyl derivatives binding within the A and B zones, the most retrieved structures are 5H7O, with a 2-(1*H*-indol-4-yl)-4-(3,4,5-trimethoxyphenyl)-1*H*-imidazo [4,5-c]pyridine ligand; 5JVD, with a (2*E*)-3-(3-hydroxy-4-methoxyphenyl)-1-(7-methoxy-2*H*-1,3-benzodioxol-5-yl)-2-methylprop-2-en-1-one; and 5LYJ, the complex of tubulin with combretastatin A-4 (Figure S25 in the Supplementary Material). These three have a trimetho-xyphenyl ring or related moiety within the A pocket. Additionally, as previously observed, the proteins in every pair of consensus poses are different for AutoDock and PLANTS, which probably reflects that the different scoring functions of both programs select different protein configurations. These results validate the use of as many protein structures as possible in the docking experiments (ensemble docking strategy) and reinforce the application of consensus scoring approaches. In summary, the docking studies support the argument that ligands can bind to the colchicine site on the tubulin, targeting zones A–C for amines **1**–**6** and zones A–B for the rest of the amide derivatives. The most favorable interactions were found for the pyridine derivatives with ethylurea and formamide functionalities, thus explaining the antimitotic activity and the structure–activity relationships observed for each series of compounds.

## 4. Conclusions

One of the main limitations of the colchicine site ligands is their low aqueous solubility due to the hydrophobic nature of the binding site. We succeeded in synthesizing a new series of water-soluble benzothiazole derivatives that are able to target the colchicine binding site, inhibit tubulin polymerization, and behave as antiproliferative agents in different kinds of cancer cells. The replacement of the olefin linker of MI-181 or CA-4 by methylamine, acetamide, formamide, or ethylurea functionalities was proven to significantly increase, in most cases, the polar surface area and the water solubilities of the synthesized 6-methoxy derivatives with respect to the reference compounds. The structure–activity relationship studies revealed that the combination of 6-methoxibenzothiazole with a pyridine ring provides an optimal potency as antiproliferative agents and tubulin polymerization inhibitors, particularly when combined with ethylurea or formamide groups. Whereas the pyridine derivatives with amino groups (**1**–**6**) showed antiproliferative IC_50_ values in the micromolar range, the best performing compounds (pyridine-based formamide and ethylurea derivatives) reached nanomolar values for some cell lines, showing an especially potent antiproliferative profile against the difficult-to-treat glioblastoma cell lines. The studies on the mechanism of action of the most potent compounds revealed that compounds **8**, **9**, and **14** disrupt the microtubule network of treated cancer cells and arrest the cell cycle at the G2/M phase 24 h after the treatment, followed by the induction of apoptotic cell death. The compounds exhibited a high selectivity toward cancer cells with respect to the non-tumoral HEK-293 cells and were not susceptible to multidrug resistance efflux pumps such as MDR1/P-gp. Binding at the colchicine site is supported by computational studies that suggest highly favorable interactions at the A–B zones for the most biologically active compounds and provide a structural explanation for the structure–activity relationships. The ensemble of results sustains the success of the strategy of design and provides new possibilities to discover synthetically accessible anticancer drugs, targeting the colchicine site of the tubulin, with improved water solubility. The acylation of the amino group of the benzothiazole derivatives was favorable in terms of water solubility and biological activity, so the incorporation of new polar functionalities on the amino group could lead to new active colchicine site ligands. The pharmacodynamic and pharmacokinetic properties of the obtained compounds show a high potential for further development. According to the high potency, aqueous solubility, and selecti-vity against glioblastoma cell lines of compounds **8** and **14**, they are good candidates to be used in a glioblastoma mouse orthotopic xenograft model to study their capacity to reduce the volume of the tumor. This could open new doors in the development of novel chemotherapy agents for the treatment of glioblastoma.

## Figures and Tables

**Table 1:** Experimental aqueous solubility, calculated topological polar surface area (TPSA), and calculated logarithm of the partition coefficient (Log P).

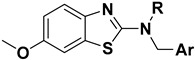					
No.	Ar	R	Solubility ^1^ (µg/mL)	TPSA ^2^ (Å^2^)	Log P*_o_*_/*w*_ ^2^
**1**	2-Pyr	H	48	75.28	2.83
**2**	3-Pyr	H	57	75.28	2.81
**3**	4-Pyr	H	55	75.28	2.79
**4**	Imid	H	86	91.07	2.44
**5**	Ph	H	5	62.39	3.64
**6**	Naph	H	3	62.39	4.47
**7**	2-Pyr	CONHEt	98	95.59	2.79
**8**	3-Pyr	CONHEt	102	95.59	2.70
**9**	4-Pyr	CONHEt	106	95.59	2.69
**10**	Imid	CONHEt	178	111.38	2.29
**11**	Ph	CONHEt	16	82.70	3.46
**12**	Naph	CONHEt	7	82.70	4.31
**13**	2-Pyr	CHO	90	83.56	2.50
**14**	3-Pyr	CHO	95	83.56	2.51
**15**	4-Pyr	CHO	93	83.56	2.51
**16**	Imid	CHO	165	99.35	2.16
**17**	Ph	CHO	12	70.67	3.23
**18**	Naph	CHO	6	70.67	4.15
**19**	2-Pyr	COMe	87	83.56	2.58
**20**	3-Pyr	COMe	93	83.56	2.54
**21**	4-Pyr	COMe	90	83.56	2.54
**22**	Imid	COMe	171	99.35	2.25
**23**	Ph	COMe	13	70.67	3.29
**24**	Naph	COMe	8	70.67	4.19
**MI181**			5.6	54.02	4.04
**CA-4**			1.1	57.15	3.27
**ABT-751**			40	108.93	2.27

^1^ Water solubilities were measured using the shaking flask methodology in buffered media at pH 7.0. Data are shown as the mean of three independent experiments. Standard deviations were, in all cases, below 1% of the mean values. ^2^ TPSA and LogP*_o_*_/*w*_ values were calculated using SwissAdme software [42].

**Table 2:** Antiproliferative activity against human tumor cell lines and the non-tumorigenic cell line HEK-293, expressed as the IC_50_ in µM ^1^.

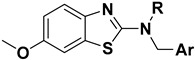												
No.	Ar	R	HeLa	HepG2	MCF7	U87MG	T98G	GL261	LNCaP	PC-3	HT-29	HEK-293
**1**	2-Pyr	H	1.78	2.40	1.53	1.61	2.40	2.17	1.52	2.11	3.51	>10
**2**	3-Pyr	H	1.27	1.19	1.17	1.32	2.11	1.91	1.43	1.55	2.23	>10
**3**	4-Pyr	H	1.45	1.23	1.74	1.51	2.27	2.03	2.01	3.31	3.39	>10
**4**	Imid	H	9.33	>10	>10	>10	>10	>10	>10	>10	>10	>10
**5**	Ph	H	4.36	5.78	6.31	8.31	>10	7.25	5.97	>10	>10	>10
**6**	Naph	H	3.71	4.51	4.23	6.54	>10	6.25	4.32	>10	>10	>10
**7**	2-Pyr	CONHEt	0.275	0.198	0.251	0.062	0.125	0.096	0.247	0.330	0.303	8.81
**8**	3-Pyr	CONHEt	0.085	0.094	0.034	0.009	0.052	0.025	0.088	0.161	0.208	7.97
**9**	4-Pyr	CONHEt	0.209	0.103	0.203	0.065	0.181	0.077	0.221	0.313	0.311	8.03
**10**	Imid	CONHEt	>10	>10	>10	>10	>10	>10	>10	>10	>10	>10
**11**	Ph	CONHEt	2.71	2.45	2.04	2.91	>10	2.93	2.37	>10	>10	>10
**12**	Naph	CONHEt	>10	>10	>10	>10	>10	>10	>10	>10	>10	>10
**13**	2-Pyr	CHO	0.331	0.321	0.221	0.254	0.327	0.195	0.315	0.423	0.557	9.31
**14**	3-Pyr	CHO	0.107	0.083	0.015	0.037	0.075	0.035	0.093	0.195	0.322	8.22
**15**	4-Pyr	CHO	0.311	0.239	0.207	0.214	0.257	0.260	0.285	0.412	0.471	8.51
**16**	Imid	CHO	>10	>10	>10	>10	>10	>10	>10	>10	>10	>10
**17**	Ph	CHO	3.40	5.55	5.02	5.27	>10	6.41	4.01	>10	>10	>10
**18**	Naph	CHO	>10	>10	>10	>10	>10	>10	>10	>10	>10	>10
**19**	2-Pyr	COMe	1.55	1.88	2.03	1.41	2.44	1.38	2.07	3.01	2.54	>10
**20**	3-Pyr	COMe	1.03	0.813	0.911	0.632	0.825	0.622	1.39	1.62	1.52	9.90
**21**	4-Pyr	COMe	1.22	1.49	1.97	1.27	2.30	1.59	1.81	2.63	2.23	>10
**22**	Imid	COMe	>10	>10	>10	>10	>10	>10	>10	>10	>10	>10
**23**	Ph	COMe	3.90	4.29	6.30	7.51	>10	6.33	4.95	>10	>10	>10
**24**	Naph	COMe	>10	>10	>10	>10	>10	>10	>10	>10	>10	>10
**ABT-751**			0.388	0.327	0.180	0.375	0.421	0.309	0.325	0.422	0.213	1.23
**TMZ**			>10	>10	>10	135	241	179	>10	>10	>10	>10
**DTX**			0.051	0.055	0.013	0.022	0.059	0.035	0.010	0.020	0.307	1.55

^1^ IC_50_ is the required drug concentration to inhibit the growth of the corresponding cell line by 50% with respect to the untreated cells (negative controls) 72 h after drug exposure. Values were obtained from concentration–response curves using the MTT assay as described in the experimental section. Data are shown as the mean of three independent experiments performed in triplicate. Standard deviations were, in all cases, below 1% of the mean values.

**Table 3:** Antiproliferative activity against human tumor HT-29 cells, expressed as the IC_50_ values in µM, in the presence of 10 µM verapamil.

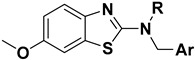							
No.	Ar	R	IC_50_ ^1^	No.	Ar	R	IC_50_ ^1^
**1**	2-Pyr	H	3.47	**13**	2-Pyr	CHO	0.540
**2**	3-Pyr	H	2.15	**14**	3-Pyr	CHO	0.307
**3**	4-Pyr	H	3.27	**15**	4-Pyr	CHO	0.459
**4**	Imid	H	>10	**16**	Imid	CHO	>10
**5**	Ph	H	>10	**17**	Ph	CHO	>10
**6**	Naph	H	>10	**18**	Naph	CHO	>10
**7**	2-Pyr	CONHEt	0.315	**19**	2-Pyr	COMe	2.93
**8**	3-Pyr	CONHEt	0.215	**20**	3-Pyr	COMe	1.48
**9**	4-Pyr	CONHEt	0.305	**21**	4-Pyr	COMe	2.55
**10**	Imid	CONHEt	>10	**22**	Imid	COMe	>10
**11**	Ph	CONHEt	>10	**23**	Ph	COMe	>10
**12**	Naph	CONHEt	>10	**24**	Naph	COMe	>10
**ABT-751**			0.250	**TMZ**			>10

^1^ IC_50_ value determined in human colon adenocarcinoma HT-29 cell line in the presence of 10 µM verapamil, a P-gp/MDR1 inhibitor. Data are shown as the mean of three independent experiments performed in triplicate. Standard deviations were, in all cases, below 1% of the mean values.

**Table 4:** Effect of the compounds on tubulin polymerization inhibition (TPI).

No.	Ar	R	% TPI	IC_50_	No.	Ar	R	% TPI ^1^	IC_50_ ^2^
**1**	2-Pyr	H	37	>10	**13**	2-Pyr	CHO	80	5.0
**2**	3-Pyr	H	42	>10	**14**	3-Pyr	CHO	87	4.1
**3**	4-Pyr	H	29	>10	**15**	4-Pyr	CHO	85	4.5
**4**	Imid	H	0	>10	**16**	Imid	CHO	0	>10
**5**	Ph	H	0	>10	**17**	Ph	CHO	4	>10
**6**	Naph	H	7	>10	**18**	Naph	CHO	0	>10
**7**	2-Pyr	CONHEt	87	4.2	**19**	2-Pyr	COMe	43	>10
**8**	3-Pyr	CONHEt	100	2.9	**20**	3-Pyr	COMe	57	8.1
**9**	4-Pyr	CONHEt	96	3.8	**21**	4-Pyr	COMe	41	>10
**10**	Imid	CONHEt	0	>10	**22**	Imid	COMe	0	>10
**11**	Ph	CONHEt	6	>10	**23**	Ph	COMe	3	>10
**12**	Naph	CONHEt	0	>10	**24**	Naph	COMe	0	>10
**ABT-751**				4.4	**MI-181**				4.6
**CA-4**				2.8					

^1^ Percentage of TPI, with respect to the negative control, for compounds tested at 10 µM. Data are shown as the mean of two independent experiments performed in triplicate. Standard deviations were, in all cases, below 1% of the mean values. ^2^ Concentration inhibiting 50% of microtubular protein polymerization in vitro.

## Data Availability

Data sharing is not applicable to this article.

## Supplementary Materials

The following supporting information can be downloaded at https://www.mdpi.com/article/10.3390/pharmaceutics15061698/s1.

## References

[1] World Health Organization. https://www.who.int/news-room/fact-sheets/detail/cancer.

[2] Dolgin E. (2022). Cancer drug approvals and setbacks in 2022. Nat. Cancer.

[3] Yamauchi T., Uzui K., Shigemi H., Negoro E., Yoshida A., Ueda T. (2013). Aurora B inhibitor barasertib and cytarabine exert a greater-than-additive cytotoxicity in acute myeloid leukemia cells. Cancer Sci..

[4] Yang Y., Yang W.S., Yu T., Yi Y.S., Park J.G., Jeong D., Kim J.H., Oh J.S., Yoon K., Kim J.H. (2014). Novel anti-inflammatory function of NSC95397 by the suppression of multiple kinases. Biochem. Pharmacol..

[5] Murase Y., Ono H., Ogawa K., Yoshioka R., Ishikawa Y., Ueda H., Akahoshi K., Ban D., Kudo A., Tanaka S. (2021). Inhibitor library screening identifies ispinesib as a new potential chemotherapeutic agent for pancreatic cancers. Cancer Sci..

[6] Qin Z., Li X., Zhang J., Tang J., Han P., Xu Z., Yu Y., Yang C., Wang C., Xu T. (2016). Chemotherapy with or without estramustine for treatment of castration-resistant prostate cancer: A systematic review and meta-analysis. Medicine.

[7] Zhu L., Chen L. (2019). Progress in research on paclitaxel and tumor immunotherapy. Cell. Mol. Biol. Lett..

[8] Yang C.H., Horwitz S.B. (2017). Taxol^®^: The First Microtubule Stabilizing Agent. Int. J. Mol. Sci..

[9] Haque A., Rahman M.A., Faizi M.S.H., Khan M.S. (2018). Next Generation Antineoplastic Agents: A Review on Structurally Modified Vinblastine (VBL) Analogues. Curr. Med. Chem..

[10] Dumontet C., Jordan M.A. (2010). Microtubule-binding agents: A dynamic field of cancer therapeutics. Nat. Rev. Drug Discov..

[11] Choudhary S., Kaku K., Robles A.J., Hamel E., Mooberry S.L., Gangjee A. (2023). Simple monocyclic pyrimidine analogs as microtubule targeting agents binding to the colchicine site. Bioorg. Med. Chem..

[12] Chen Z.H., Xu R.M., Zheng G.H., Jin Y.Z., Li Y., Chen X.Y., Tian Y.S. (2023). Development of Combretastatin A-4 Analogues as Potential Anticancer Agents with Improved Aqueous Solubility. Molecules.

[13] Grillone K., Riillo C., Rocca R., Ascrizzi S., Spanò V., Scionti F., Polerà N., Maruca A., Barreca M., Juli G. (2022). The New Microtubule-Targeting Agent SIX2G Induces Immunogenic Cell Death in Multiple Myeloma. Int. J. Mol. Sci..

[14] Pérez-Pérez M.-J., Priego E.M., Bueno O., Solange Martins M., Canela M.-D., Liekens S. (2016). Blocking blood flow to solid tumors by destabilizing tubulin: An approach to targeting tumor growth. J. Med. Chem..

[15] Wang J., Miller D.D., Li W. (2022). Molecular interactions at the colchicine binding site in tubulin: An X-ray crystallography perspective. Drug Discov. Today.

[16] Álvarez R., Medarde M., Peláez P. (2014). New ligands of the tubulin colchicine site based on X-ray structures. Curr. Top. Med. Chem..

[17] Massarotti A., Coluccia A., Silvestri R., Sorba G., Brancale A. (2012). The tubulin colchicine domain: A molecular modeling perspective. Chem. Med. Chem..

[18] Wang Y., Zhang H., Gigant B., Yu Y., Wu Y., Chen X., Lai Q., Yang Z., Chen Q., Yang J. (2016). Structures of a diverse set of colchicine binding site inhibitors in complex with tubulin provide a rationale for drug discovery. FEBS J..

[19] Luo Y., Hradil V.P., Frost D.J., Rosenberg S.H., Gordon G.B., Morgan S.J., Gagne G.D., Cox B.F., Tahir S.K., Fox G.B. (2009). ABT-751, a novel tubulin-binding agent, decreases tumor perfusion and disrupts tumor vasculature. Anti-Cancer Drugs.

[20] Young S.L., Chaplin D.J. (2004). Combretastatin A4 phosphate: Background and current clinical status. Expert. Opin. Investig. Drugs.

[21] González M., Ellahioui Y., Álvarez R., Gallego-Yerga L., Caballero E., Vicente-Blázquez A., Ramudo L., Marín M., Sanz C., Medarde M. (2019). The Masked Polar Group Incorporation (MPGI) Strategy in Drug Design: Effects of Nitrogen Substitutions on Combretastatin and Isocombretastatin Tubulin Inhibitors. Molecules.

[22] Vicente-Blázquez A., González M., Medarde M., Mollinedo F., Peláez R. (2021). New indolesulfonamide derivatives targeting the colchicine site of tubulin: Synthesis, anti-tumour activity, structure-activity relationships, and molecular modelling. J. Enzyme Inhib. Med. Chem..

[23] Álvarez R., Aramburu L., Gajate C., Vicente-Blázquez A., Mollinedo F., Medarde M., Peláez R. (2021). Methylsulfanylpyridine based diheteroaryl isocombretastatin analogs as potent anti-proliferative agents. Eur. J. Med. Chem..

[24] McLoughlin E.C., O’Boyle N.M. (2020). Colchicine-Binding Site Inhibitors from Chemistry to Clinic: A Review. Pharmaceuticals.

[25] Álvarez R., Aramburu L., Gajate C., Vicente-Blázquez A., Mollinedo F., Medarde M., Peláez R. (2020). Potent colchicine-site ligands with improved intrinsic solubility by replacement of the 3,4,5-trimethoxyphenyl ring with a 2-methylsulfanyl-6-methoxypyridine ring. Bioorg. Chem..

[26] De la Roche N.M., Mühlethaler T., Di Martino R.M.C., Ortega J.A., Gioia D., Roy B., Prota A.E., Steinmetz M.O., Cavalli A. (2022). Novel fragment-derived colchicine-site binders as microtubule-destabilizing agents. Eur. J. Med. Chem..

[27] Amoroso R., De Lellis L., Florio R., Moreno N., Agamennone M., De Filippis B., Giampietro L., Maccallini C., Fernández I., Recio R. (2022). Benzothiazole Derivatives Endowed with Antiproliferative Activity in Paraganglioma and Pancreatic Cancer Cells: Structure-Activity Relationship Studies and Target Prediction Analysis. Pharmaceuticals.

[28] Ammazzalorso A., D’Angelo A., Giancristofaro A., De Filippis B., Di Matteo M., Fantacuzzi M., Giampietro L., Linciano P., Maccallini C., Amoroso R. (2012). Fibrate-derived *N*-(methylsulfonyl)amides with antagonistic properties on PPARα. Eur. J. Med. Chem..

[29] Ammazzalorso A., De Lellis L., Florio R., Bruno I., De Filippis B., Fantacuzzi M., Giampietro L., Maccallini C., Perconti S., Verginelli F. (2017). Cytotoxic effect of a family of peroxisome proliferator-activated receptor antagonists in colorectal and pancreatic cancer cell lines. Chem. Biol. Drug Des..

[30] Ashraf M., Shaik T.B., Malik M.S., Syed R., Mallipeddi P.L., Vardhan M.V.P.S.V., Kamal A. (2016). Design and synthesis of cis-restricted benzimidazole and benzothiazole mimics of combretastatin A-4 as antimitotic agents with apoptosis inducing ability. Bioorg. Med. Chem. Lett..

[31] Shaik T.B., Hussaini S.M.A., Nayak V.L., Sucharitha M.L., Malik M.S., Kamal A. (2017). Rational design and synthesis of 2-anilinopyridinyl-benzothiazole Schiff bases as antimitotic agents. Bioorg. Med. Chem. Lett..

[32] Subba Rao A.V., Swapna K., Shaik S.P., Lakshma Nayak V., Srinivasa Reddy T., Sunkari S., Shaik T.B., Bagul C., Kamal A. (2017). Synthesis and biological evaluation of cis-restricted triazole/tetrazole mimics of combretastatin-benzothiazole hybrids as tubulin polymerization inhibitors and apoptosis inducers. Bioorg. Med. Chem..

[33] Joshi R., Mukherjee D.D., Chakrabarty S., Martin A., Jadhao M., Chakrabarti G., Sarkar A., Ghosh S.K. (2018). Unveiling the Potential of Unfused Bichromophoric Naphthalimide to Induce Cytotoxicity by Binding to Tubulin: Breaks Monotony of Naphthalimides as Conventional Intercalators. J. Phys. Chem. B.

[34] Fu D.J., Liu S.M., Li F.H., Yang J.J., Li J. (2020). Antiproliferative benzothiazoles incorporating a trimethoxyphenyl scaffold as novel colchicine site tubulin polymerization inhibitors. J. Enzyme Inhib. Med. Chem..

[35] Song J., Gao Q.L., Wu B.W., Zhu T., Cui X.X., Jin C.J., Wang S.Y., Wang S.H., Fu D.J., Liu H.M. (2020). Discovery of tertiary amide derivatives incorporating benzothiazole moiety as anti-gastric cancer agents in vitro via inhibiting tubulin polymerization and activating the Hippo signaling pathway. Eur. J. Med. Chem..

[36] Mohapatra S., Gupta V., Mondal P., Chatterjee S., Bhunia D., Ghosh S. (2021). A Small Molecule with Bridged Carbonyl and Tri-fluoro-aceto-phenone Groups Impedes Microtubule Dynamics and Subsequently Triggers Cancer Cell Apoptosis. Chem. Med. Chem..

[37] Gao L., Meiring J.C.M., Kraus Y., Wranik M., Weinert T., Pritzl S.D., Bingham R., Ntouliou E., Jansen K.I., Olieric N. (2021). A Robust, GFP-Orthogonal Photoswitchable Inhibitor Scaffold Extends Optical Control over the Microtubule Cytoskeleton. Cell Chem. Biol..

[38] Dorléans A., Gigant B., Ravelli R.B., Mailliet P., Mikol V., Knossow M. (2009). Variations in the colchicine-binding domain provide insight into the structural switch of tubulin. Proc. Natl. Acad. Sci. USA.

[39] Vasquez R.J., Howell B., Yvon A.M., Wadsworth P., Cassimeris L. (1997). Nanomolar concentrations of nocodazole alter microtubule dynamic instability in vivo and in vitro. Mol. Biol. Cell.

[40] Senese S., Lo Y.C., Huang D., Zangle T.A., Gholkar A.A., Robert L., Homet B., Ribas A., Summers M.K., Teitell M.A. (2014). Chemical dissection of the cell cycle: Probes for cell biology and anti-cancer drug development. Cell Death Dis..

[41] McNamara D.E., Senese S., Yeates T.O., Torres J.Z. (2015). Structures of potent anticancer compounds bound to tubulin. Protein Sci..

[42] Swiss Institute of Bioinformatics. http://www.swissadme.ch/.

[43] Daina A., Michielin O., Zoete V. (2017). SwissADME: A free web tool to evaluate pharmacokinetics, drug-likeness and medicinal chemistry friendliness of small molecules. Sci. Rep..

[44] Ertl P., Rohde B., Selzer P. (2000). Fast calculation of molecular polar surface area as a sum of fragment-based contributions and its application to the prediction of drug transport properties. J. Med. Chem..

[45] Shelanski M.L., Gaskin F., Cantor C.R. (1973). Microtubule assembly in the absence of added nucleotides. Proc. Natl. Acad. Sci. USA.

[46] Posadas I., López-Hernández B., Clemente M.I., Jimenez J.L., Ortega P., de la Mata J., Gómez R., Muñoz-Fernández M.A., Ceña V. (2009). Highly efficient transfection of rat cortical neurons using carbosilane dendrimers unveils a neuroprotective role for HIF-1alpha in early chemical hypoxia-mediated neurotoxicity. Pharm. Res..

[47] González M., Alcolea P.J., Alvarez R., Medarde M., Larraga V., Peláez R. (2021). New diarylsulfonamide inhibitors of *Leishmania infantum* amastigotes. Int. J. Parasitol. Drugs Drug Resist..

[48] Berthold M.R., Cebron N., Dill F., Gabriel T.R. (2007). KNIME: The Konstanz Information Miner. Studies in Classification, Data Analysis, and Knowledge Organization.

[49] Velázquez-Libera J.L., Duran-Verdugo F., Valdes-Jimenez A., Núñez-Vivanco G., Caballero J. (2020). LigRMSD: A web server for automatic structure matching and RMSD calculations among identical and similar compounds in protein-ligand docking. Bioinformatics.

[50] Pettersen E.F., Goddard T.D., Huang C.C., Couch G.S., Greenblatt D.M., Meng E.C., Ferrin T.E. (2004). UCSF Chimera—A visualization system for exploratory research and analysis. J. Comput. Chem..

[51] Marvin 17.8 ChemAxon. http://www.chemaxon.com.

[52] OpenEye Scientific. https://www.eyesopen.com.

[53] García-Pérez C., Peláez R., Theron R., López-Perez J.L. (2017). JADOPPT: Java based AutoDock preparing and processing tool. Bioinformatics.

[54] Li Y.S., Escobar L., Zhu Y.J., Cohen Y., Ballester P., Rebek J., Yu Y. (2019). Relative hydrophilicities of cis and trans formamides. Proc. Natl. Acad. Sci. USA.

[55] Hughes J.D., Blagg J., Price D.A., Bailey S., Decrescenzo G.A., Devraj R.V., Ellsworth E., Fobian Y.M., Gibbs M.E., Gilles R.W. (2008). Physiochemical drug properties associated with in vivo toxicological outcomes. Bioorg. Med. Chem. Lett..

[56] González M., Ovejero-Sánchez M., Vicente-Blázquez A., Álvarez R., Herrero A.B., Medarde M., González-Sarmiento R., Peláez R. (2021). Microtubule Destabilizing Sulfonamides as an Alternative to Taxane-Based Chemotherapy. Int. J. Mol. Sci..

[57] González M., Ellahioui Y., Gallego L., Vicente-Blázquez A., Álvarez R., Medarde M., Peláez R. (2023). Novel amino analogs of the trimethoxyphenyl ring in potent colchicine site ligands improve solubility by the masked polar group incorporation (MPGI) strategy. Bioorg. Chem..

[58] González M., Ovejero-Sánchez M., Vicente-Blázquez A., Medarde M., González-Sarmiento R., Peláez R. (2021). Methoxy and bromo scans on N-(5-methoxyphenyl) methoxybenzenesulphonamides reveal potent cytotoxic compounds, especially against the human breast adenocarcinoma MCF7 cell line. J. Enzyme Inhib. Med. Chem..

[59] Greene L.M., O’Boyle N.M., Nolan D.P., Meegan M.J., Zisterer D.M. (2012). The vascular targeting agent Combretastatin-A4 directly induces autophagy in adenocarcinoma-derived colon cancer cells. Biochem. Pharmacol..

[60] Aprile S., Del Grosso E., Grosa G. (2010). Identification of the human UDP-glucuronosyltransferases involved in the glucuronidation of combretastatin A-4. Drug Metab. Dispos..

[61] Kavallaris M. (2010). Microtubules and resistance to tubulin-binding agents. Nat. Rev. Cancer.

[62] Kagawa S., Gu J., Honda T., McDonnell T.J., Swisher S.G., Roth J.A., Fang B. (2001). Deficiency of caspase-3 in MCF7 cells blocks Bax-mediated nuclear fragmentation but not cell death. Clin. Cancer Res..

[63] Vicente-Blázquez A., González M., Álvarez R., Del Mazo S., Medarde M., Peláez R. (2019). Antitubulin sulfonamides: The successful combination of an established drug class and a multifaceted target. Med. Res. Rev..

[64] Gallego-Yerga L., Ochoa R., Lans I., Peña-Varas C., Alegría-Arcos M., Cossio P., Ramírez D., Peláez R. (2021). Application of ensemble pharmacophore-based virtual screening to the discovery of novel antimitotic tubulin inhibitors. Comput. Struct. Biotechnol. J..

[65] Álvarez R., Aramburu L., Puebla P., Caballero E., González M., Vicente A., Medarde M., Peláez R. (2016). Pyridine Based Antitumour Compounds Acting at the Colchicine Site. Curr. Med. Chem..

[66] Álvarez R., Puebla P., Díaz J.F., Bento A.C., García-Navas R., De la Iglesia-Vicente J., Mollinedo F., Andreu J.M., Medarde M., Peláez R. (2013). Endowing indole-based tubulin inhibitors with an anchor for derivatization: Highly potent 3-substituted indolephenstatins and indoleisocombretastatins. J. Med. Chem..

[67] Korb O., Stutzle T., Exner T.E. (2009). Empirical scoring functions for advanced protein-ligand docking with PLANTS. J. Chem. Inf. Model..

[68] Forli S., Huey R., Pique M.E., Sanner M.F., Goodsell D.S., Olson A.J. (2016). Computational protein-ligand docking and virtual drug screening with the AutoDock suite. Nat. Protoc..

[69] Worldwide Protein Data Bank. http://www.wwpdb.org/.

