# Supplementary material for: Potent and Selective Benzothiazole-Based Antimitotics with Improved Water Solubility: Design, Synthesis, and Evaluation as Novel Anticancer Agents
Source: Pharmaceutics. 2023 Jun 9;15(6):1698. doi: 10.3390/pharmaceutics15061698 (PMC10301603; doi:10.3390/pharmaceutics15061698)
Supplement: Supplementary file 1 [file pharmaceutics-15-01698-s001.zip › pharmaceutics-2439300-supplementary.pdf]

Supplementary material

Potent and selective benzothiazole-based antimitotics with improved water solubility: design, synthesis, and evaluation as novel anticancer agents

Laura Gallego-Yerga [1,2,3,*], Valentín Ceña [4,5] and Rafael Peláez [1,2,3,*]

NMR spectra of compounds **1** - **24**……………………………………………………………………………………2

Supplementary figure S25……………………………………………………………………………………………27

Supplementary table S1………………………………………………………………………………………………28

^{1}H NMR

^{13}C NMR

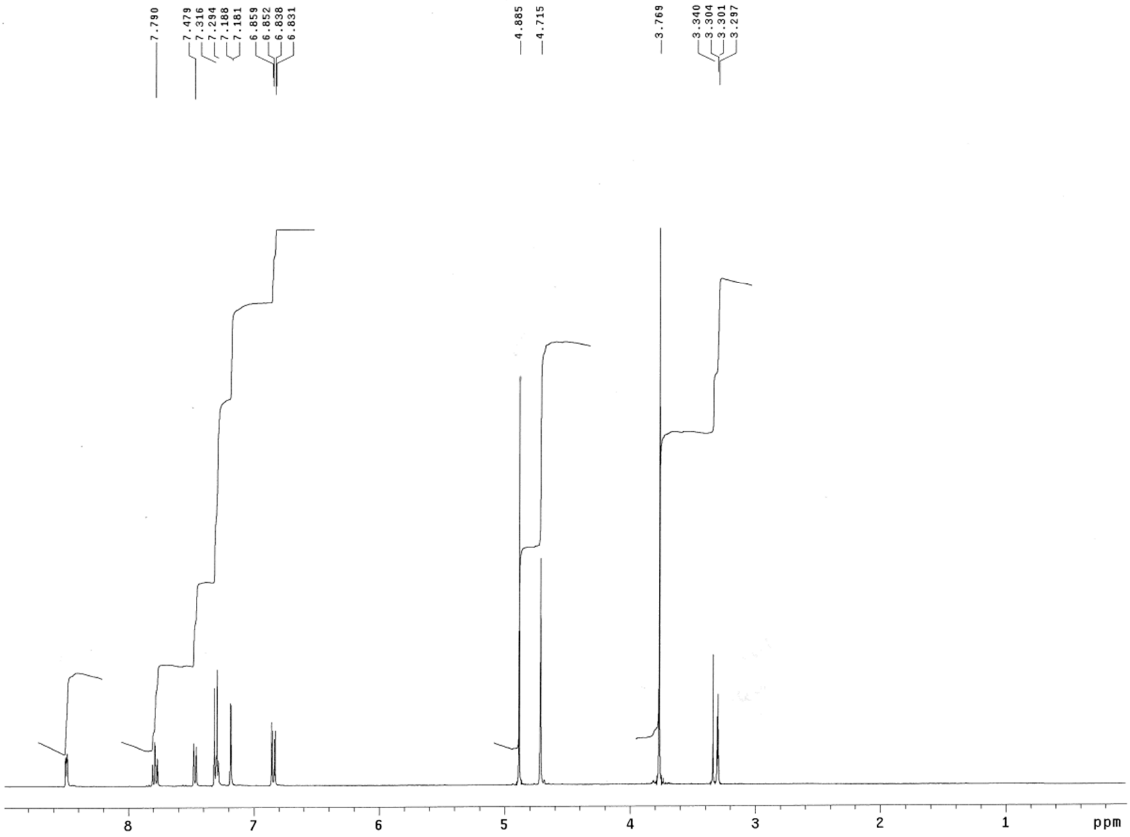

Figure S1a. ^{1}H NMR (MeOD, 400 MHz) spectrum of compound **1**.

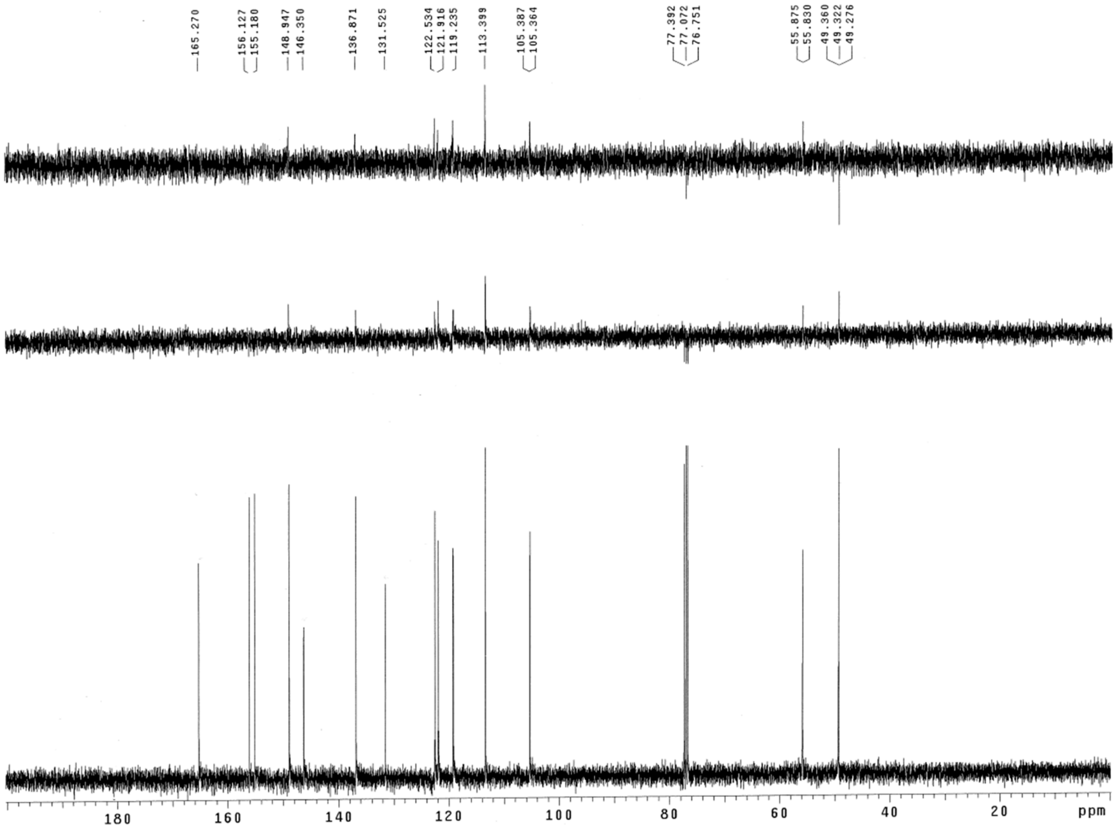

Figure S1b. ^{13}C NMR ($CDCl_3$, 100 MHz) spectra of compound **1**.

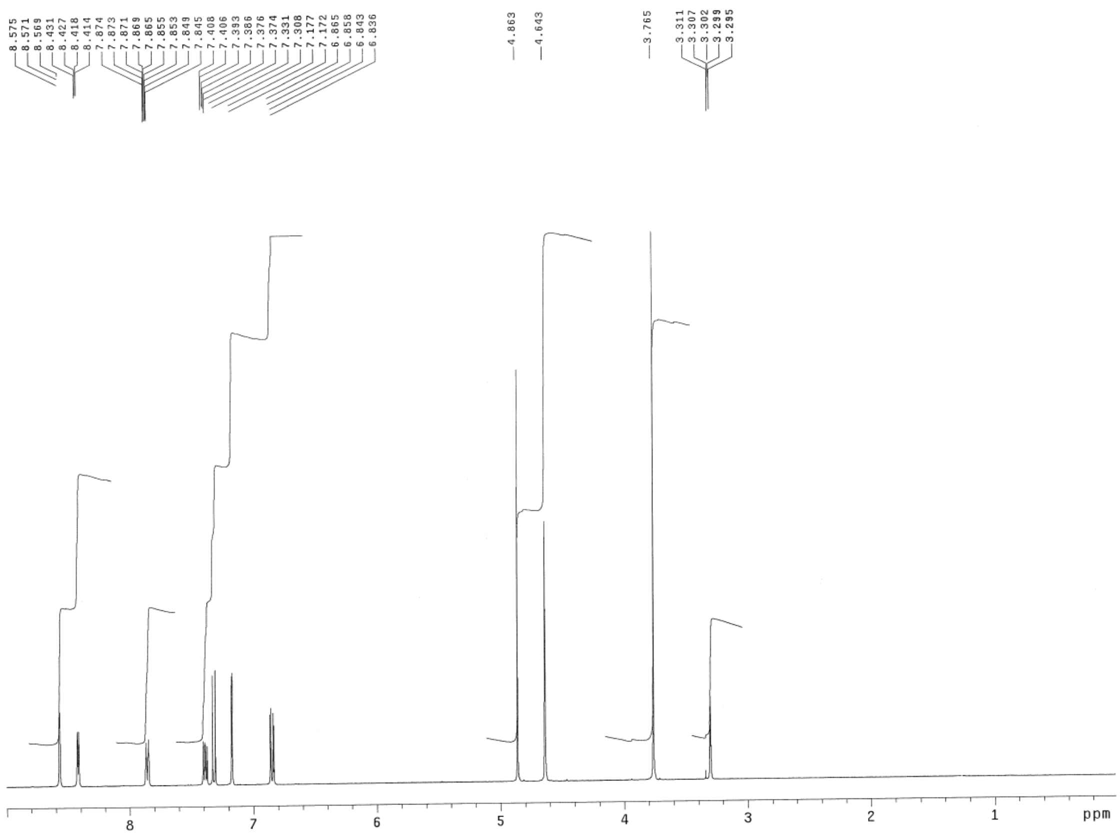

Figure S2a. ^{1}H NMR (MeOD, 400 MHz) spectrum of compound **2**.

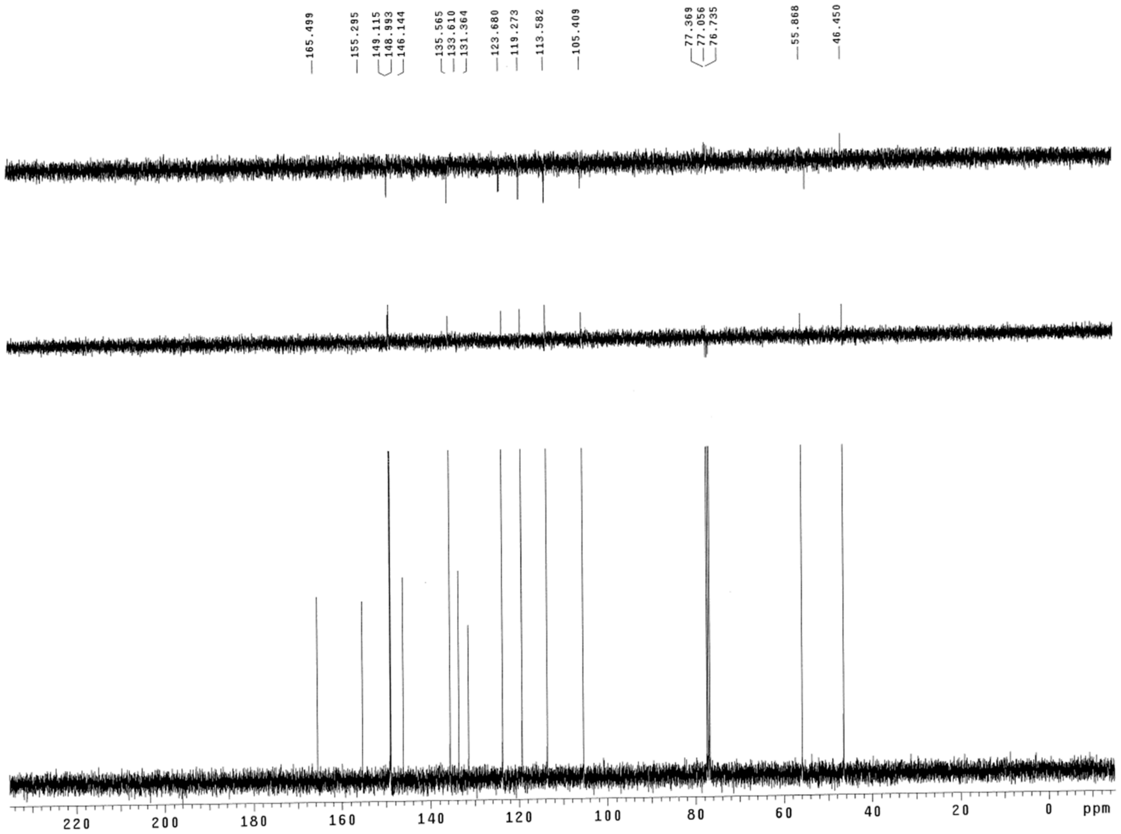

Figure S2b. ^{13}C NMR ($CDCl_3$, 100 MHz) spectra of compound **2**.

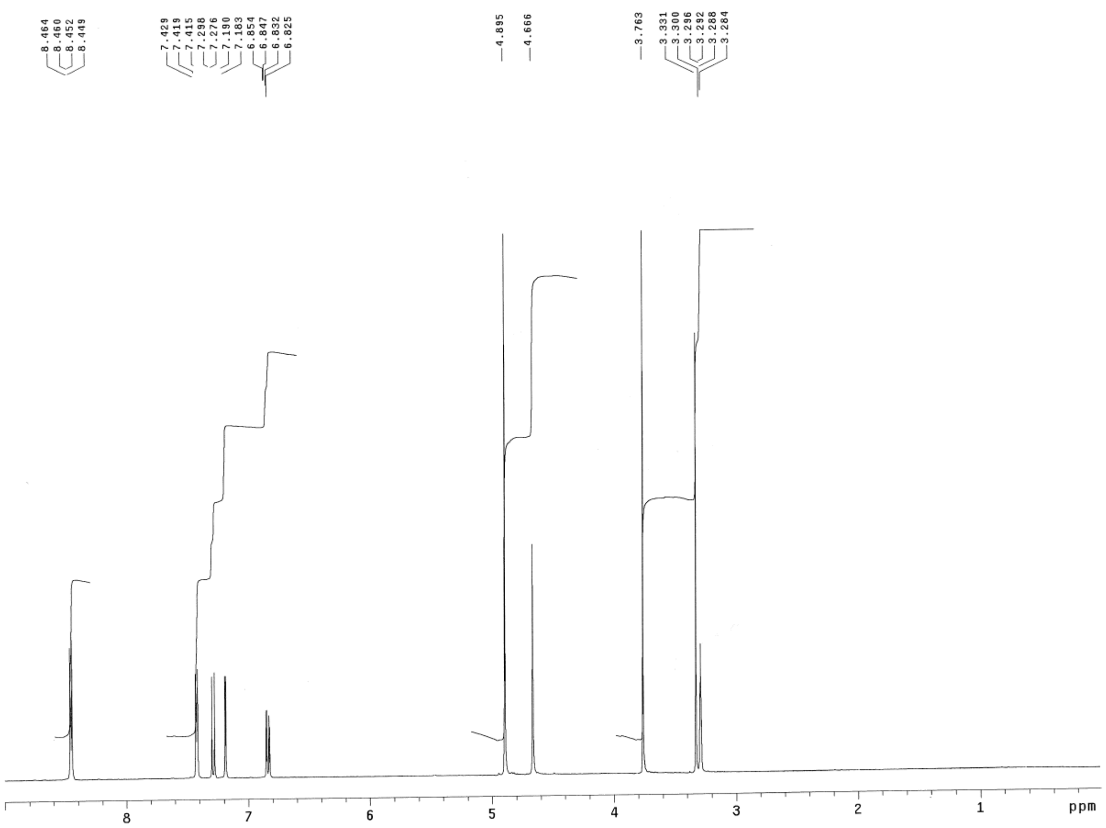

Figure S3a. ^{1}H NMR (MeOD, 400 MHz) spectrum of compound **3**.

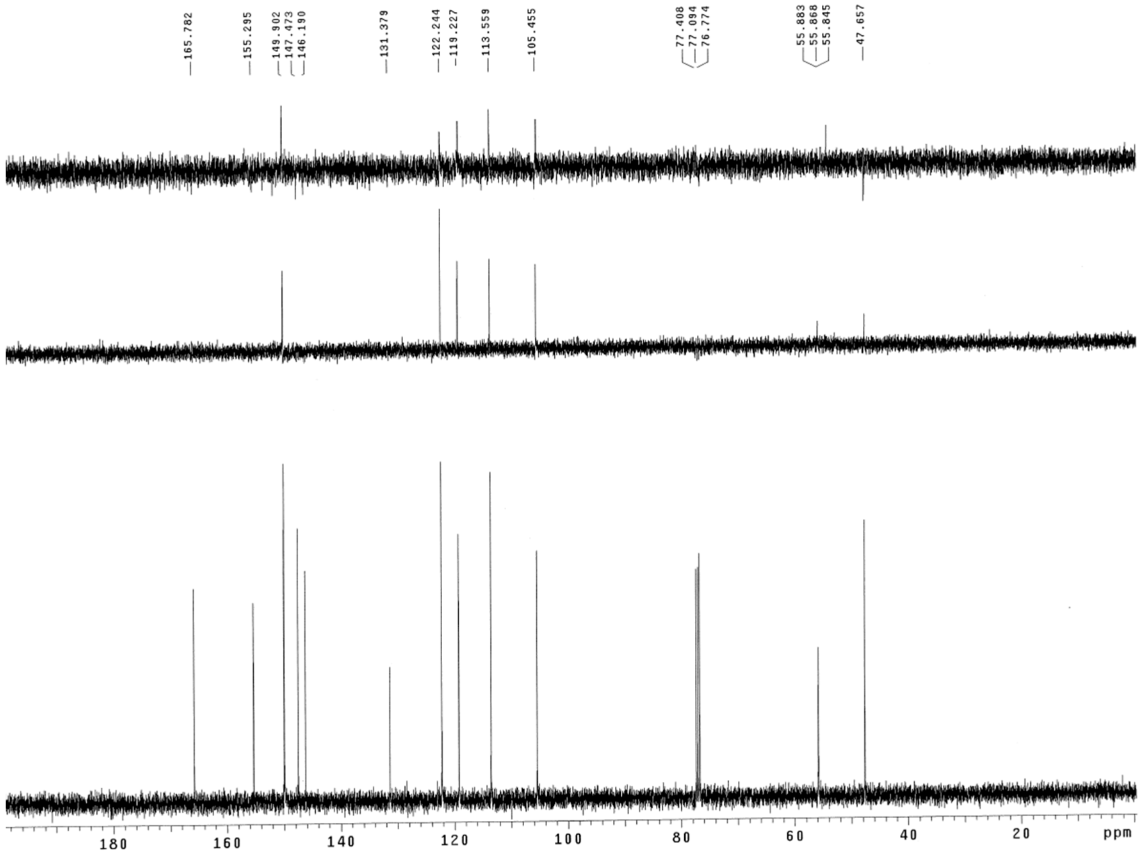

Figure S3b. ^{13}C NMR ($CDCl_3$, 100 MHz) spectra of compound **3**.

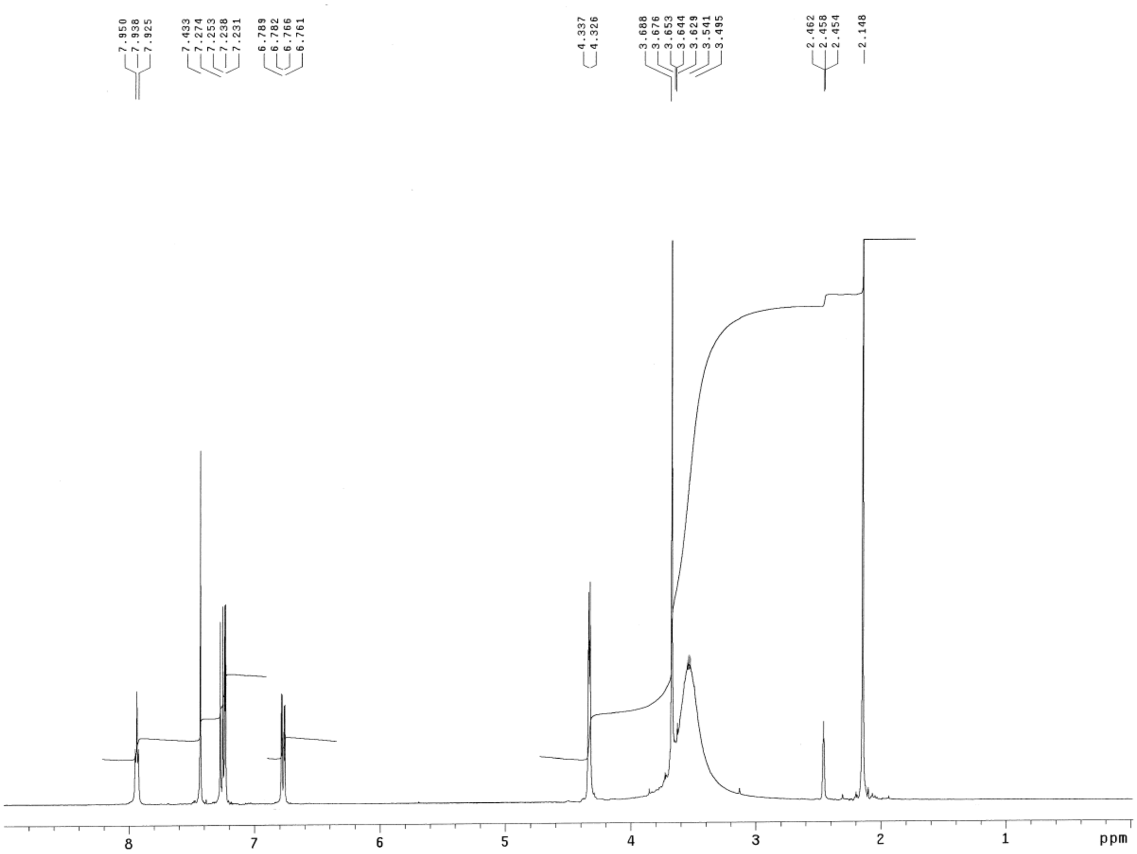

Figure S4a. ^{1}H NMR (DMSO-d_6, 400 MHz) spectrum of compound **4**.

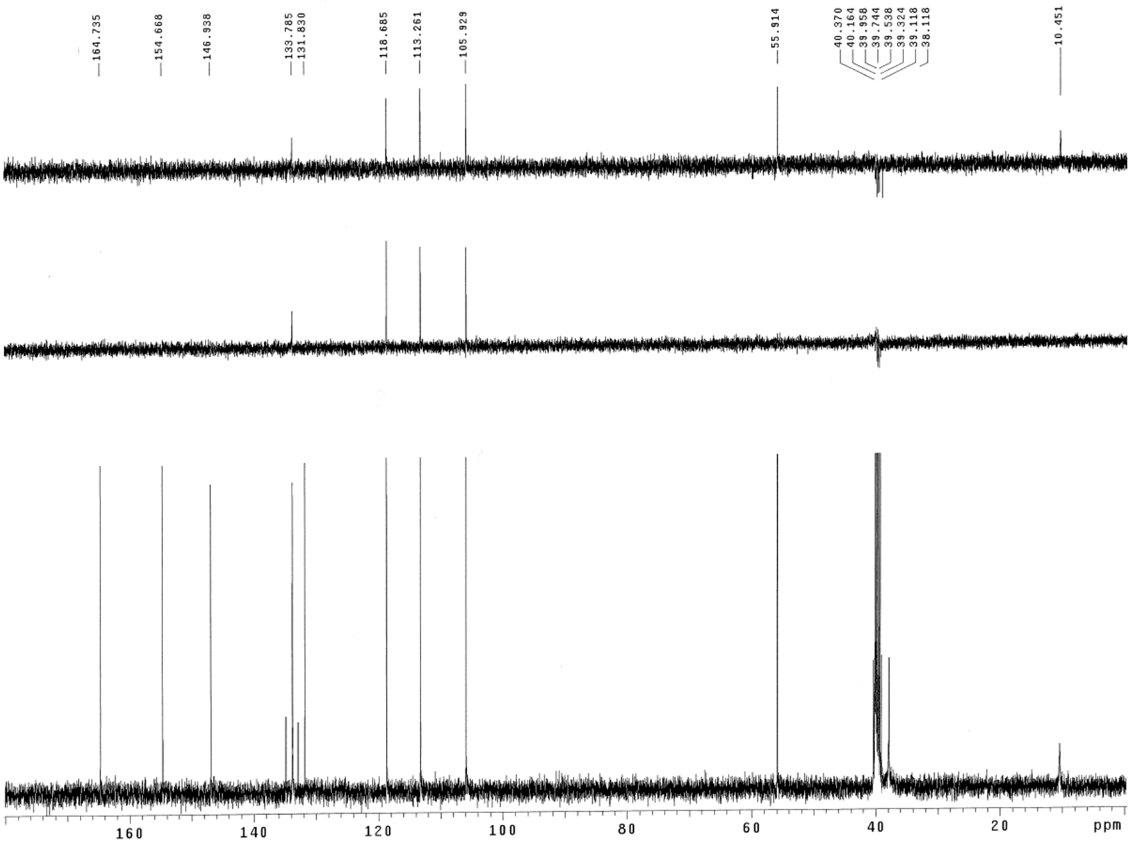

Figure S4b. ^{13}C NMR (DMSO-d_6, 100 MHz) spectra of compound **4**.

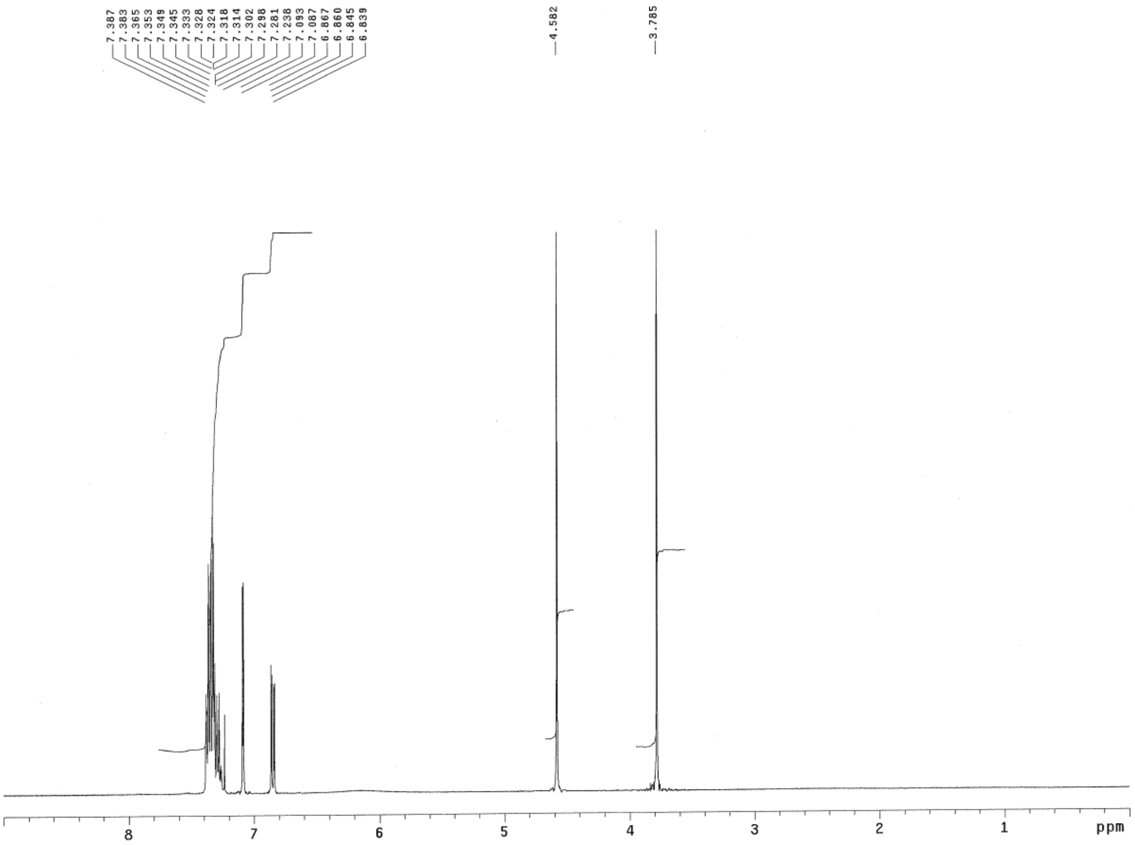

Figure S5a. ^{1}H NMR ($CDCl_3$, 400 MHz) spectrum of compound **5**.

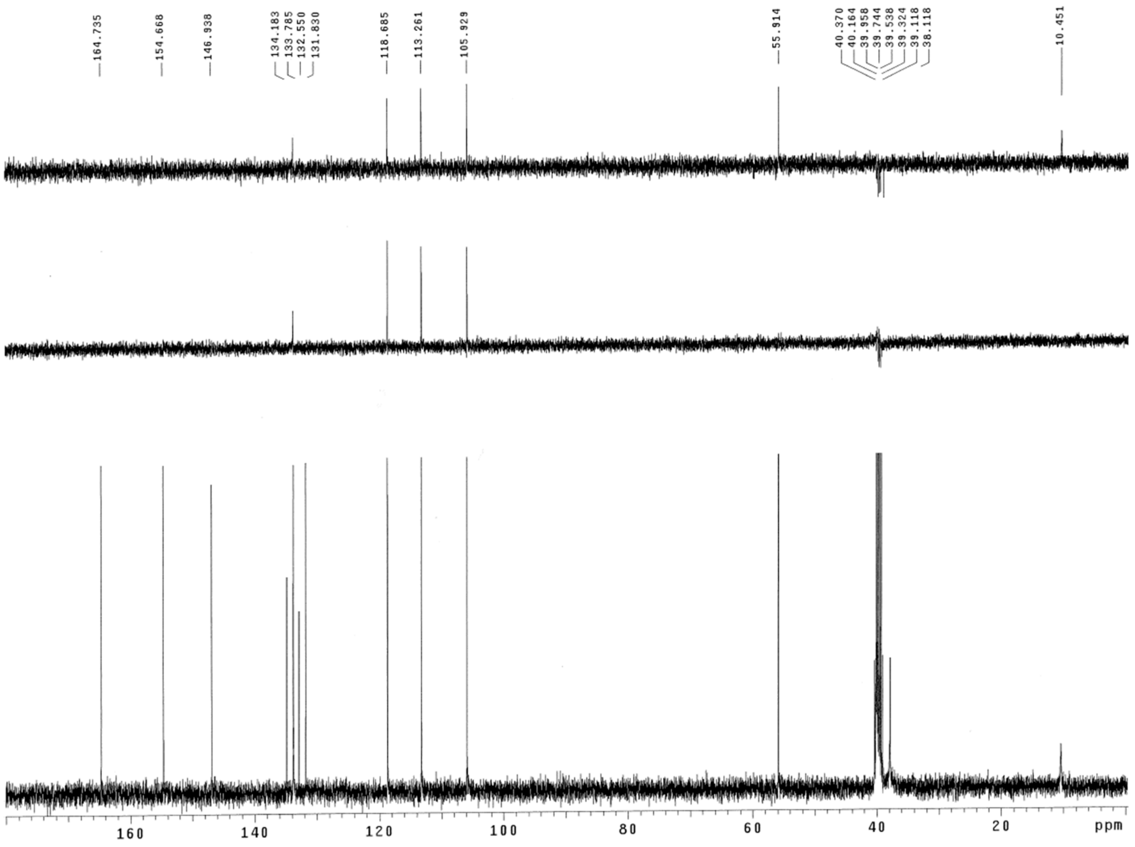

Figure S5b. ^{13}C NMR ($CDCl_3$, 100 MHz) spectra of compound **5**.

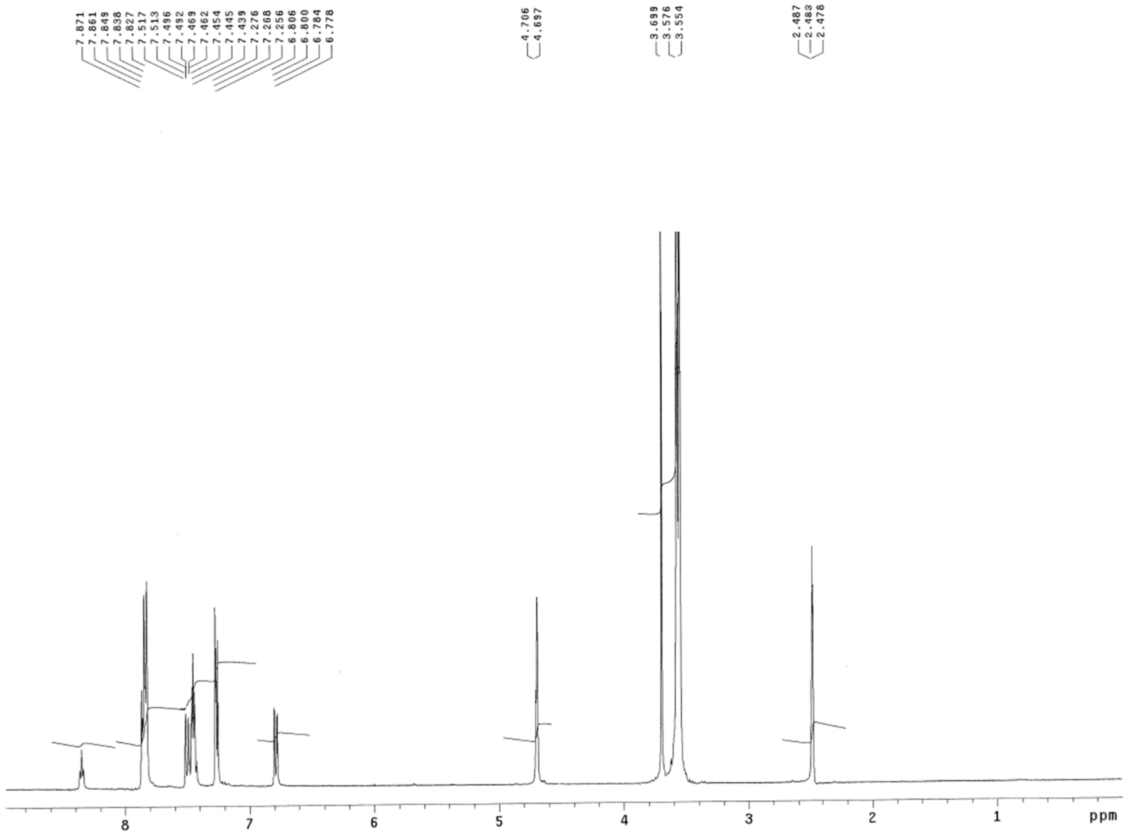

Figure S6a. ^{1}H NMR ($CDCl_3$, 400 MHz) spectrum of compound **6**.

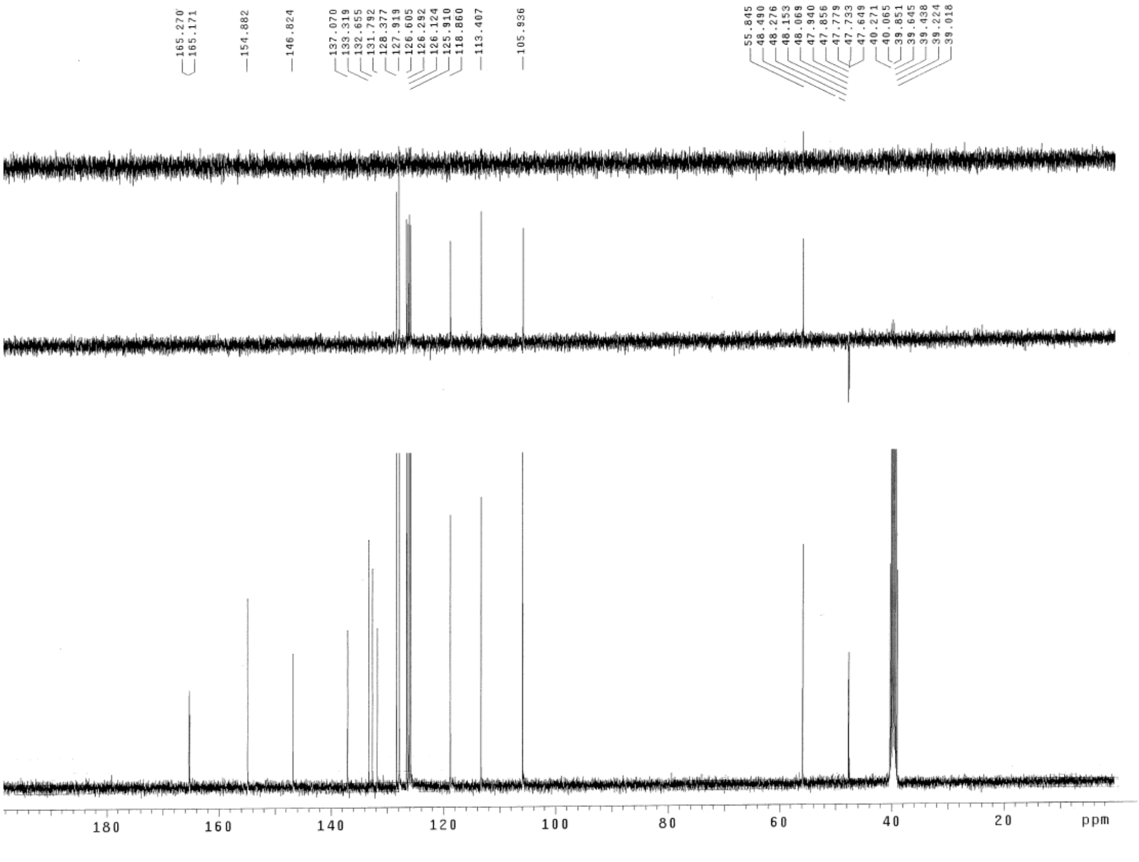

Figure S6b. ^{13}C NMR (DMSO-d_6, 100 MHz) spectra of compound **6**.

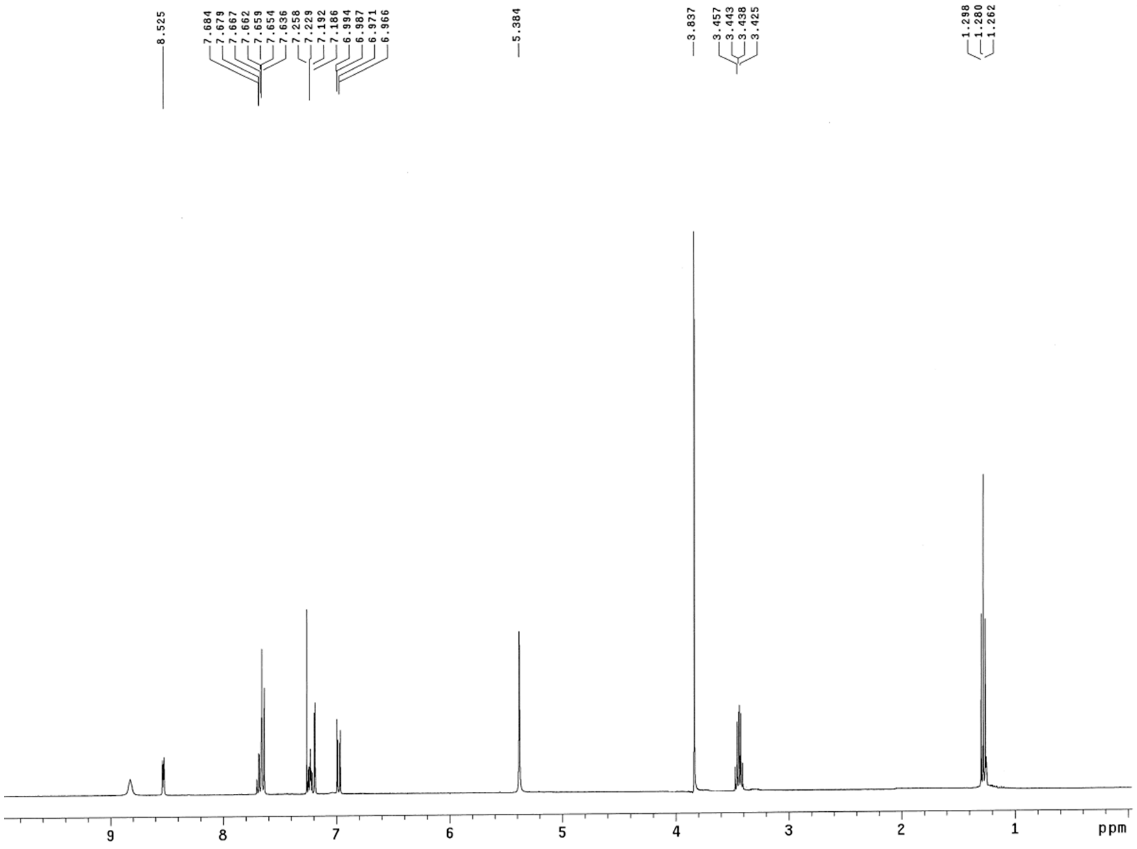

Figure S7a. ^{1}H NMR ($CDCl_3$, 400 MHz) spectrum of compound **7**.

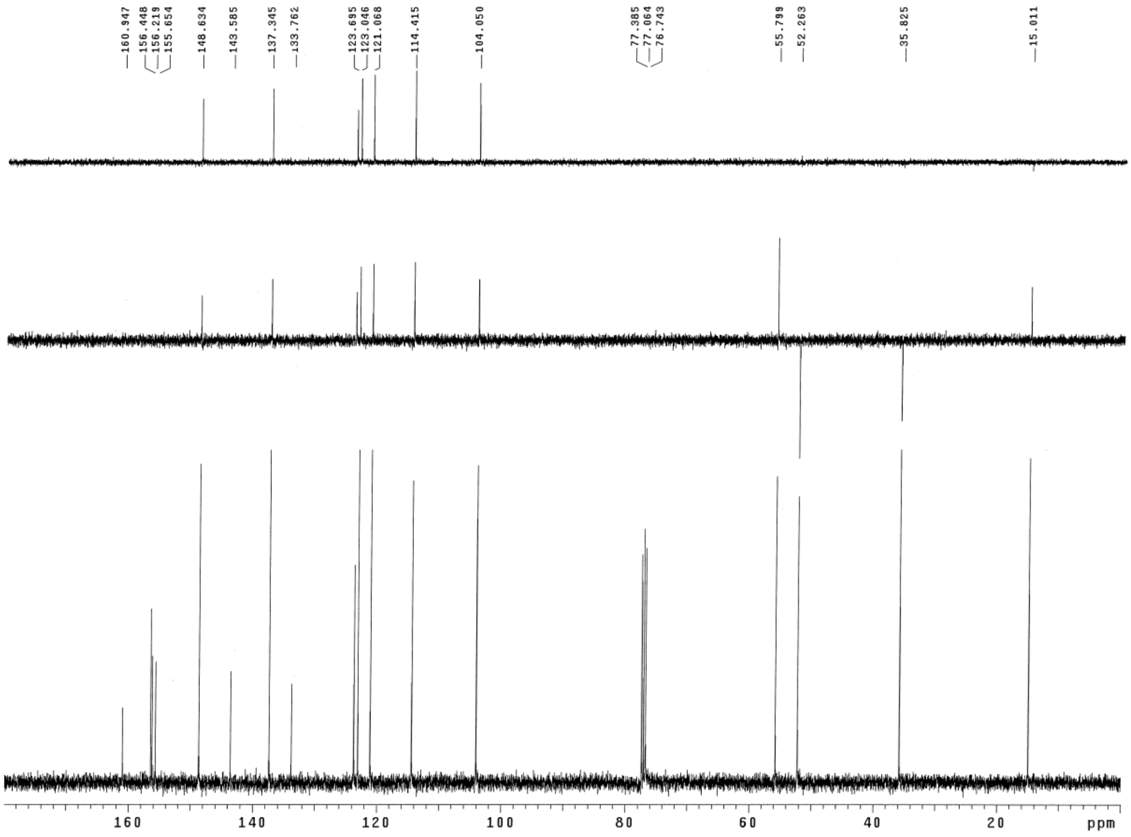

Figure S7b. ^{13}C NMR ($CDCl_3$, 100 MHz) spectra of compound **7**.

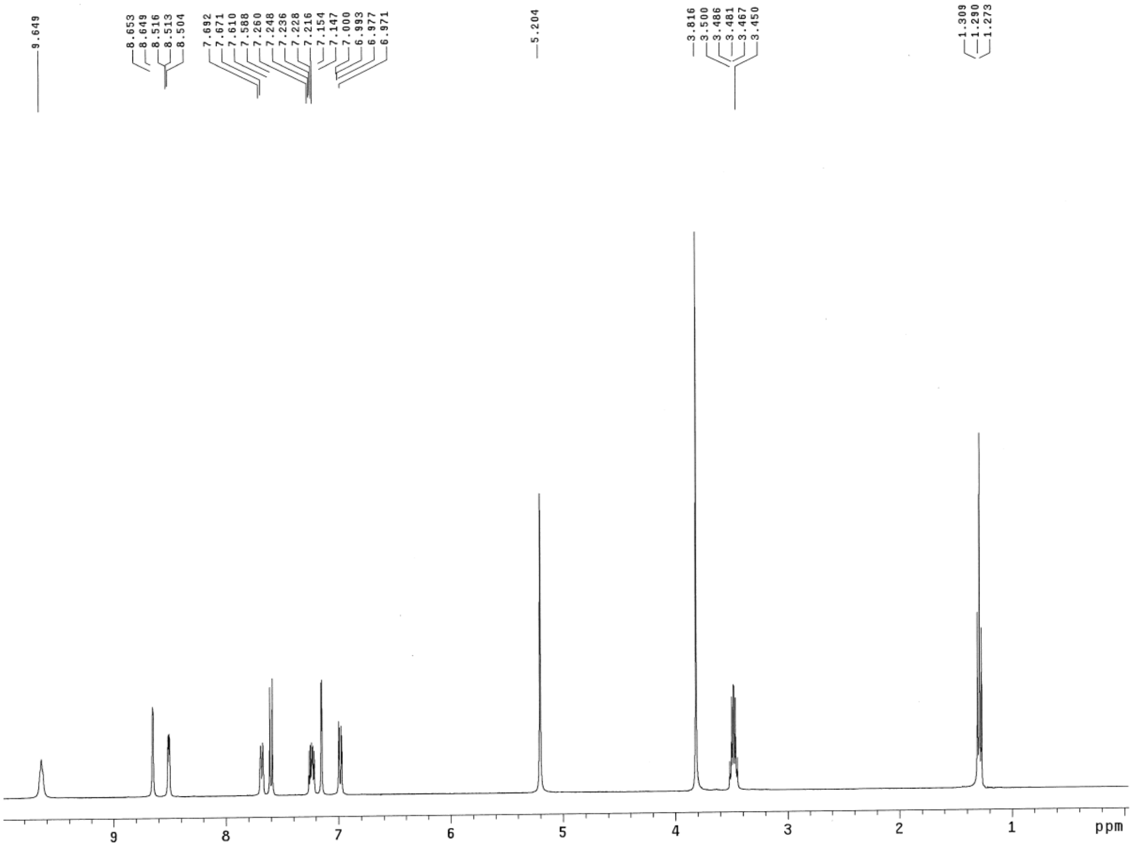

Figure S8a. ^{1}H NMR ($CDCl_3$, 400 MHz) spectrum of compound **8**.

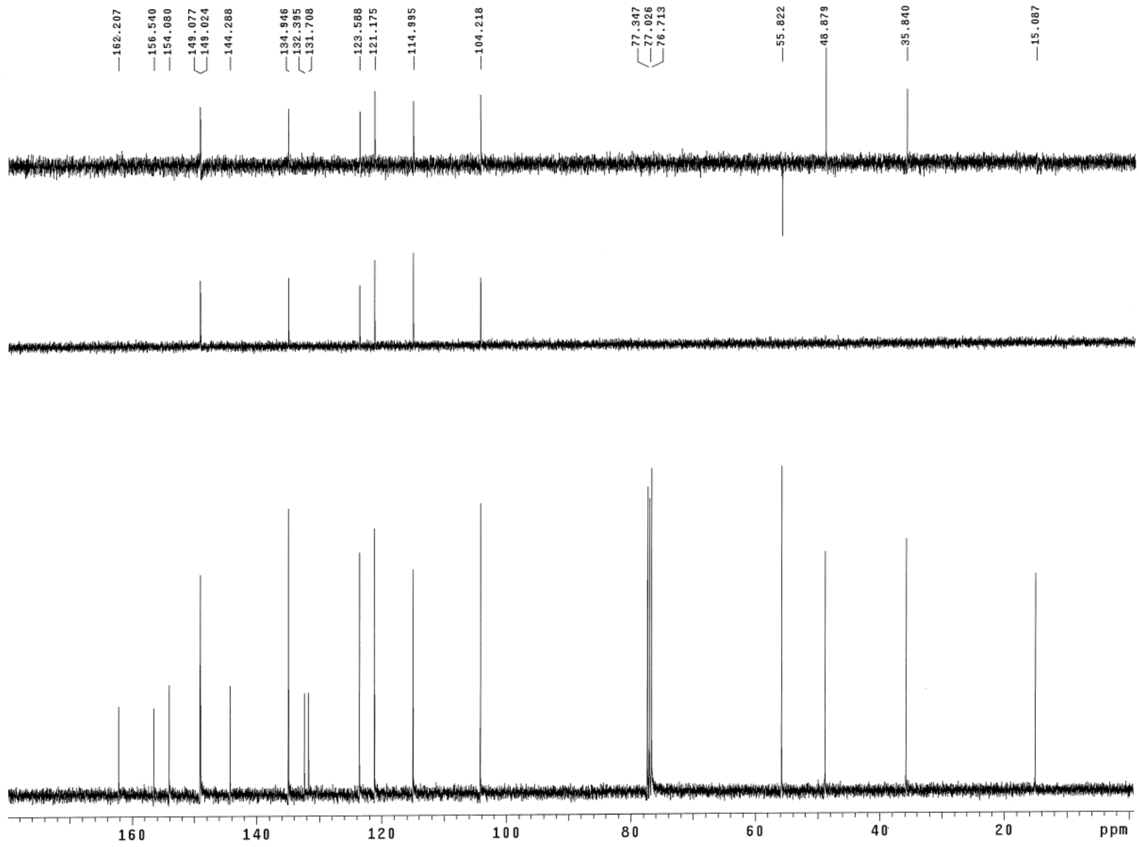

Figure S8b. ^{13}C NMR ($CDCl_3$, 100 MHz) spectra of compound **8**.

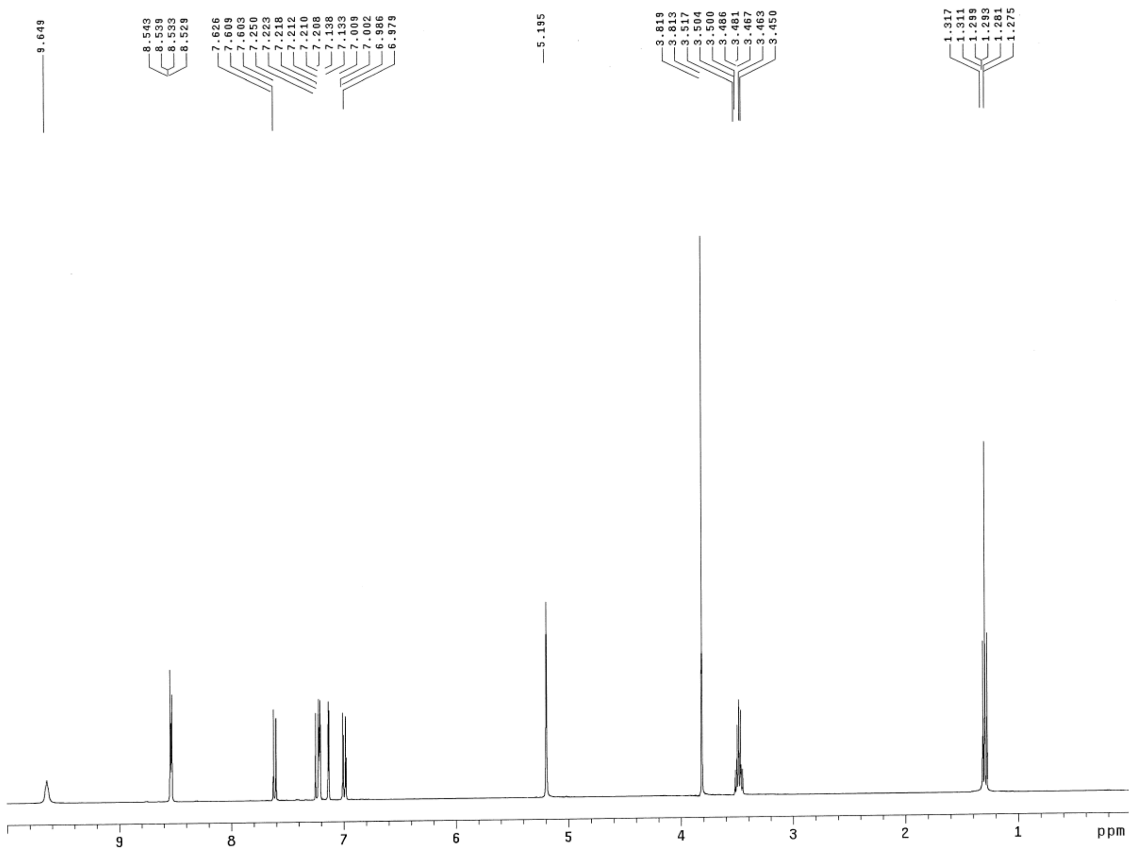

Figure S9a. ^{1}H NMR ($CDCl_3$, 400 MHz) spectrum of compound **9**.

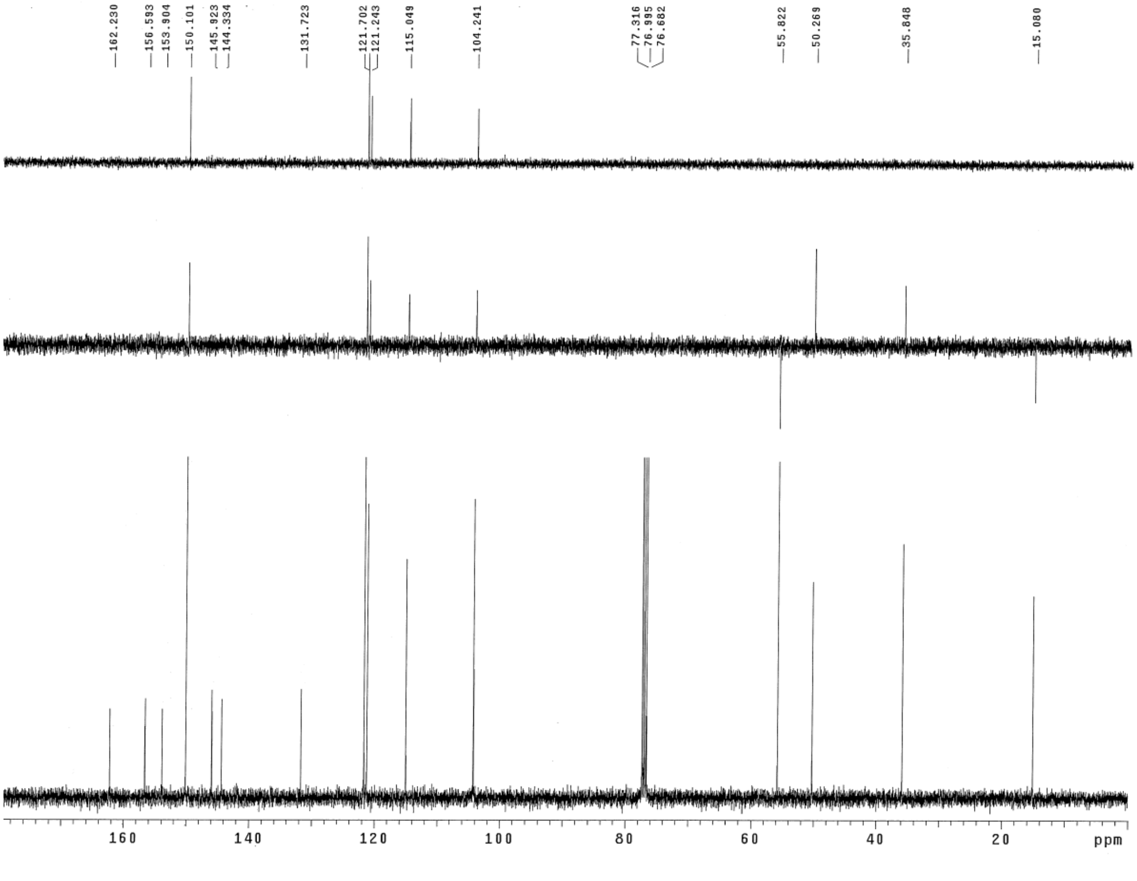

Figure S9b. ^{13}C NMR ($CDCl_3$, 100 MHz) spectra of compound **9**.

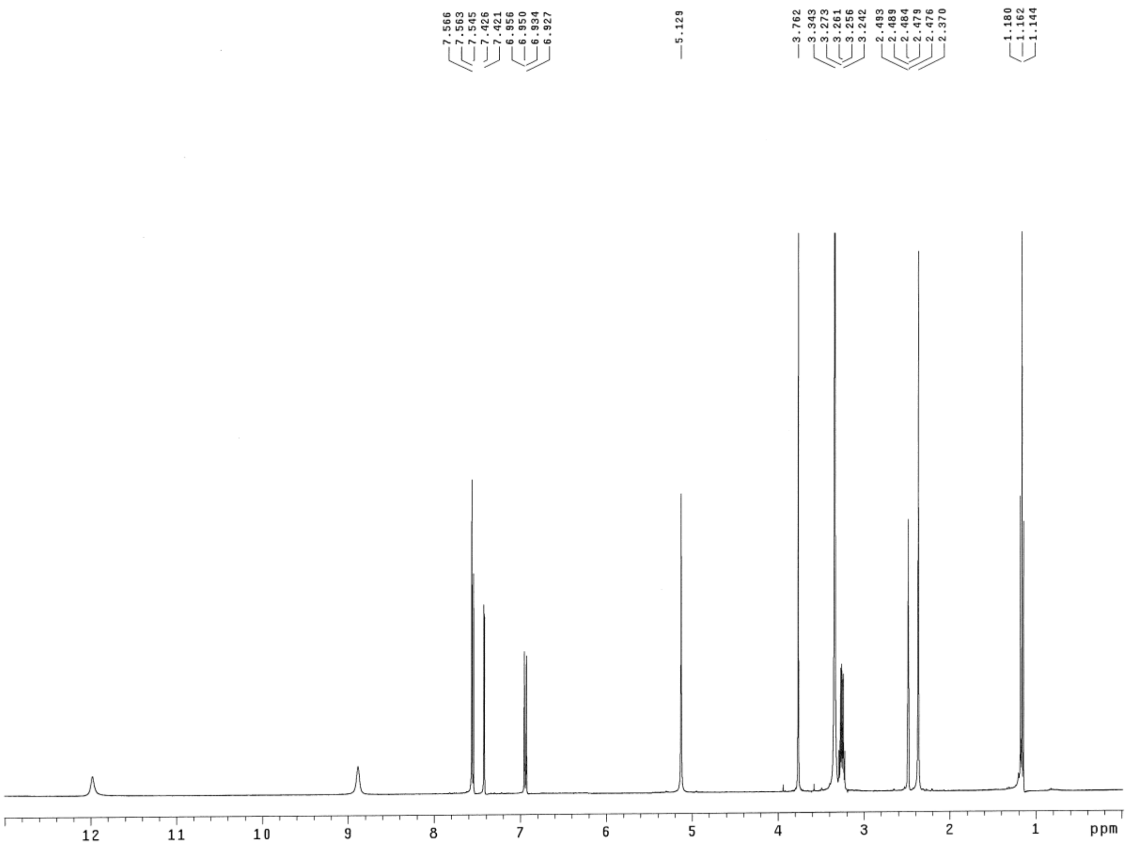

Figure S10a. ^{1}H NMR (DMSO-d_6, 400 MHz) spectrum of compound **10**.

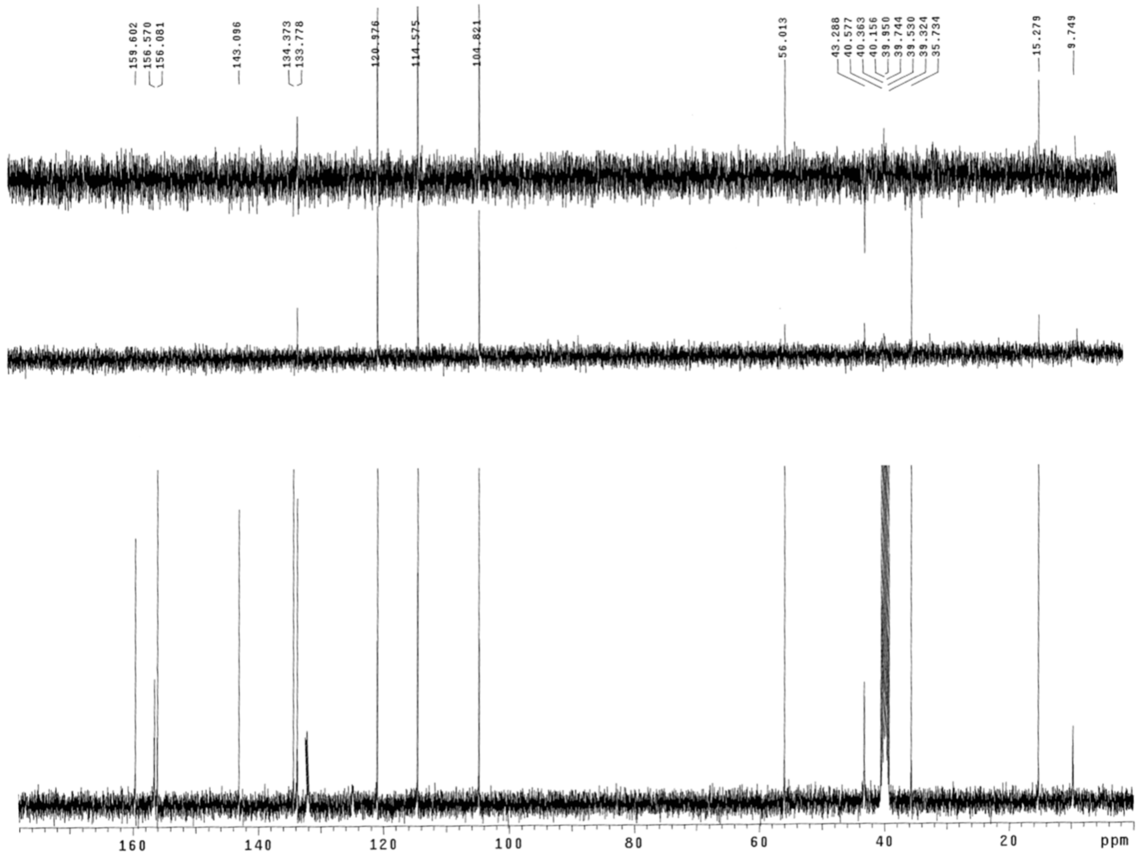

Figure S10b. ^{13}C NMR (DMSO-d_6, 100 MHz) spectra of compound **10**.

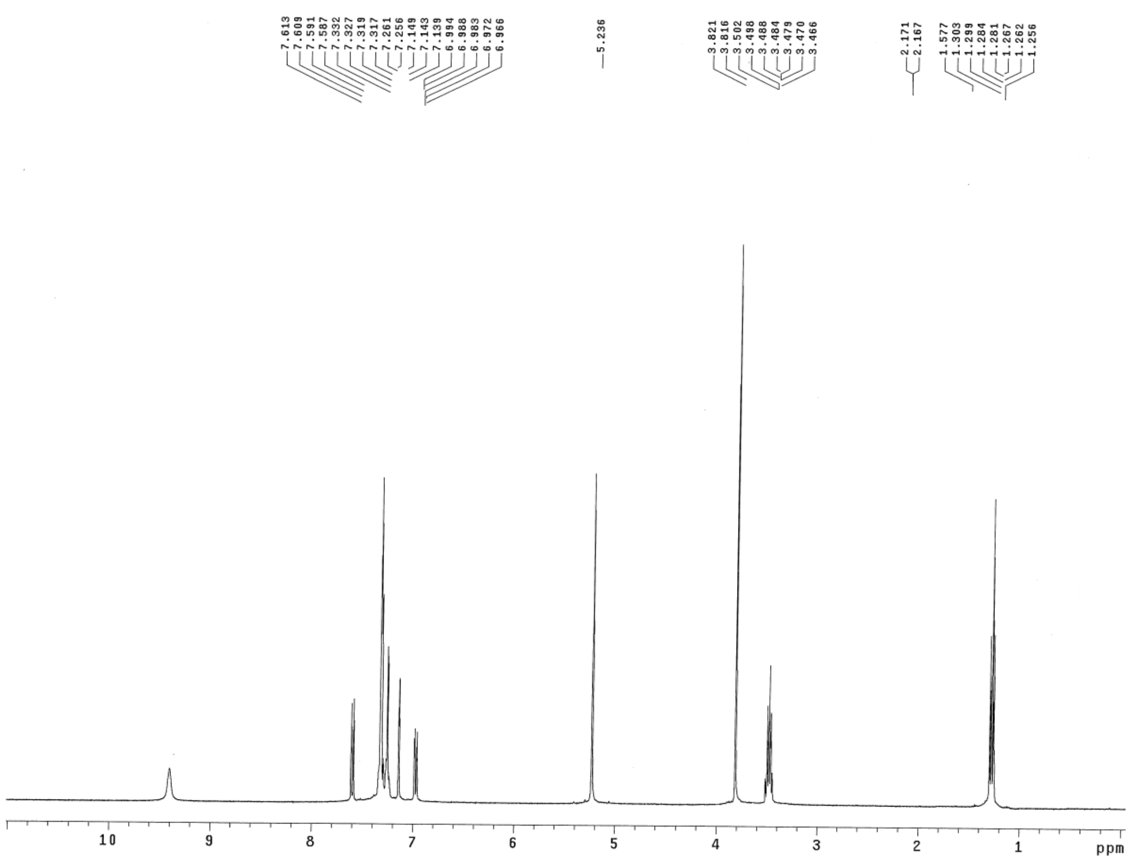

Figure S11a. ^{1}H NMR ($CDCl_3$, 400 MHz) spectrum of compound **11**.

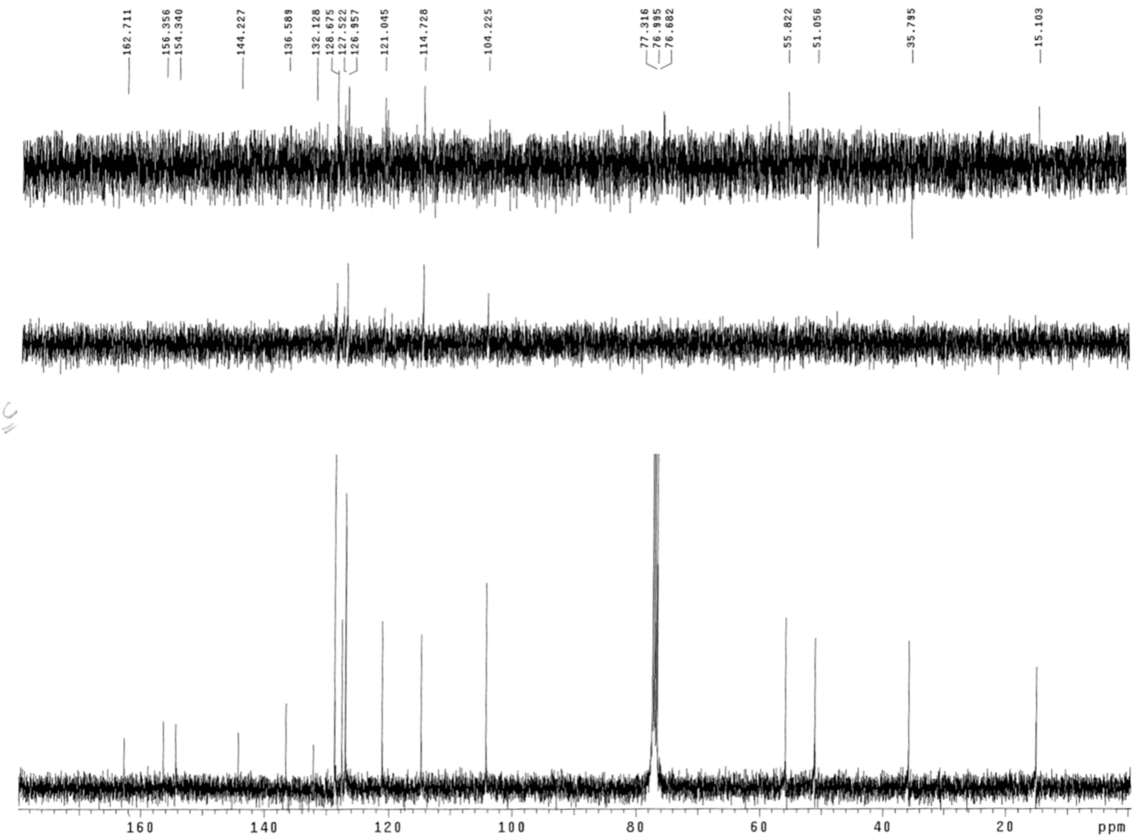

Figure S11b. ^{13}C NMR ($CDCl_3$, 100 MHz) spectra of compound **11**.

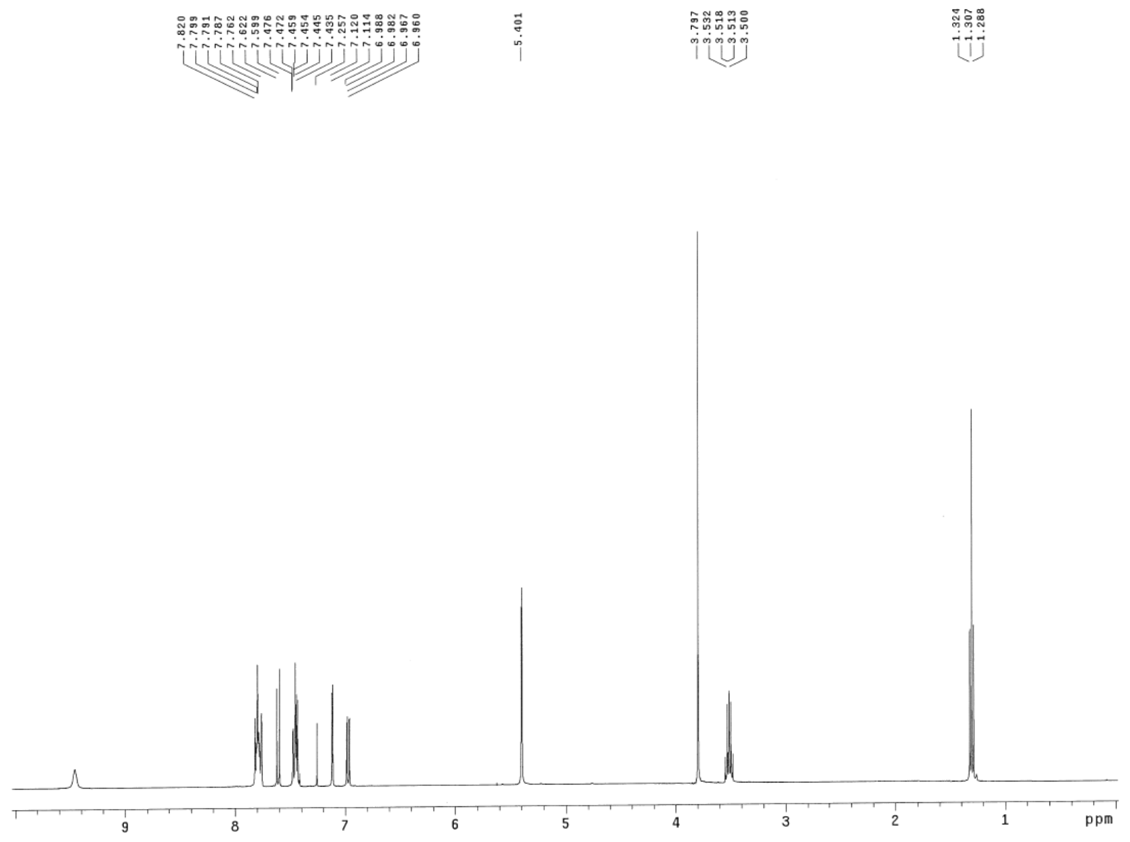

Figure S12a. ^{1}H NMR ($CDCl_3$, 400 MHz) spectrum of compound **12**.

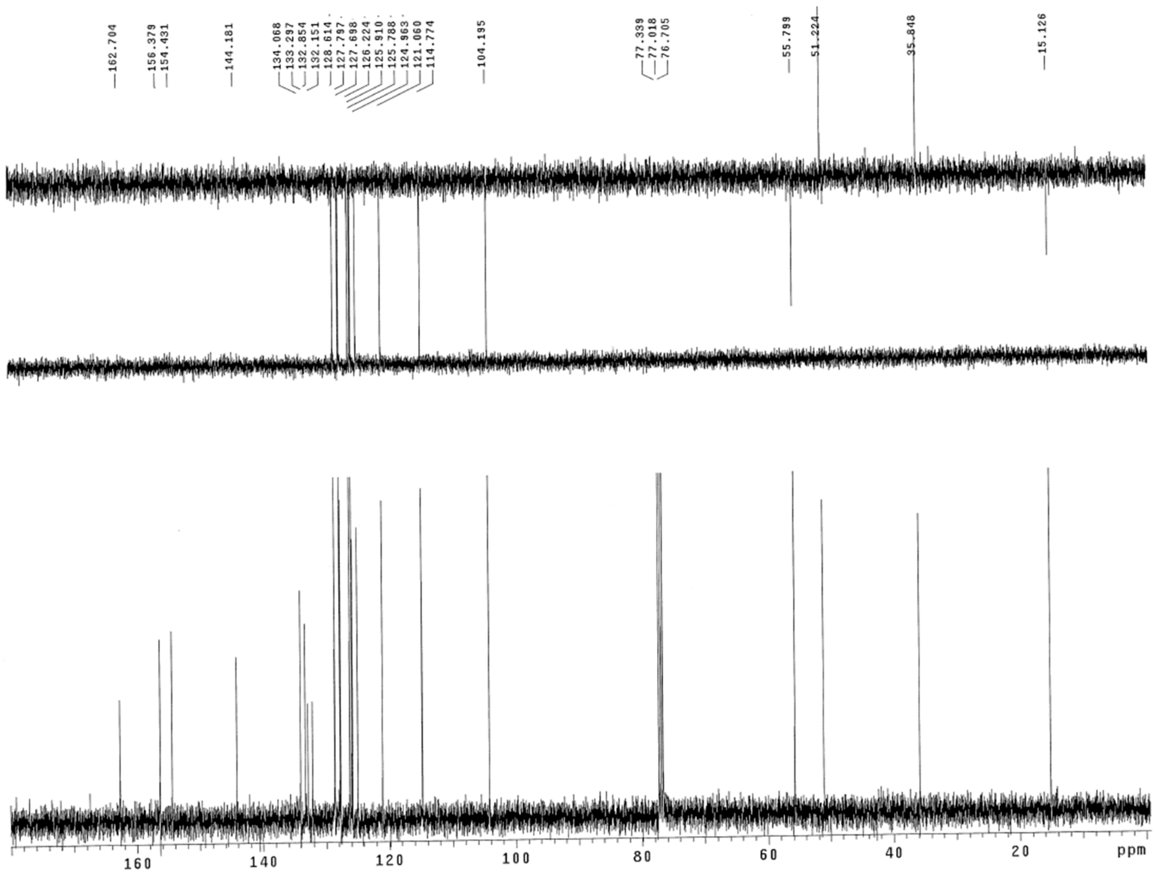

Figure S12b. ^{13}C NMR ($CDCl_3$, 100 MHz) spectra of compound **12.**

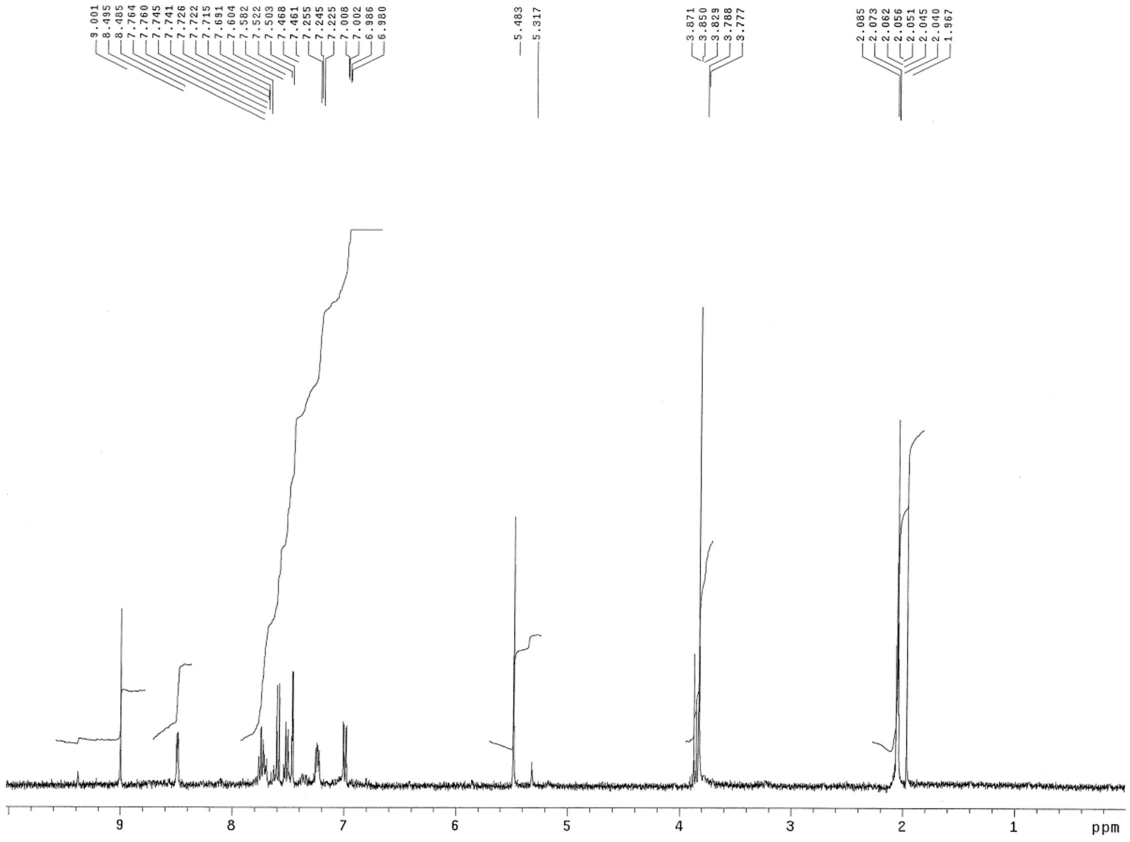

Figure S13a. ^{1}H NMR (acetone-d_6, 400 MHz) spectrum of compound **13**.

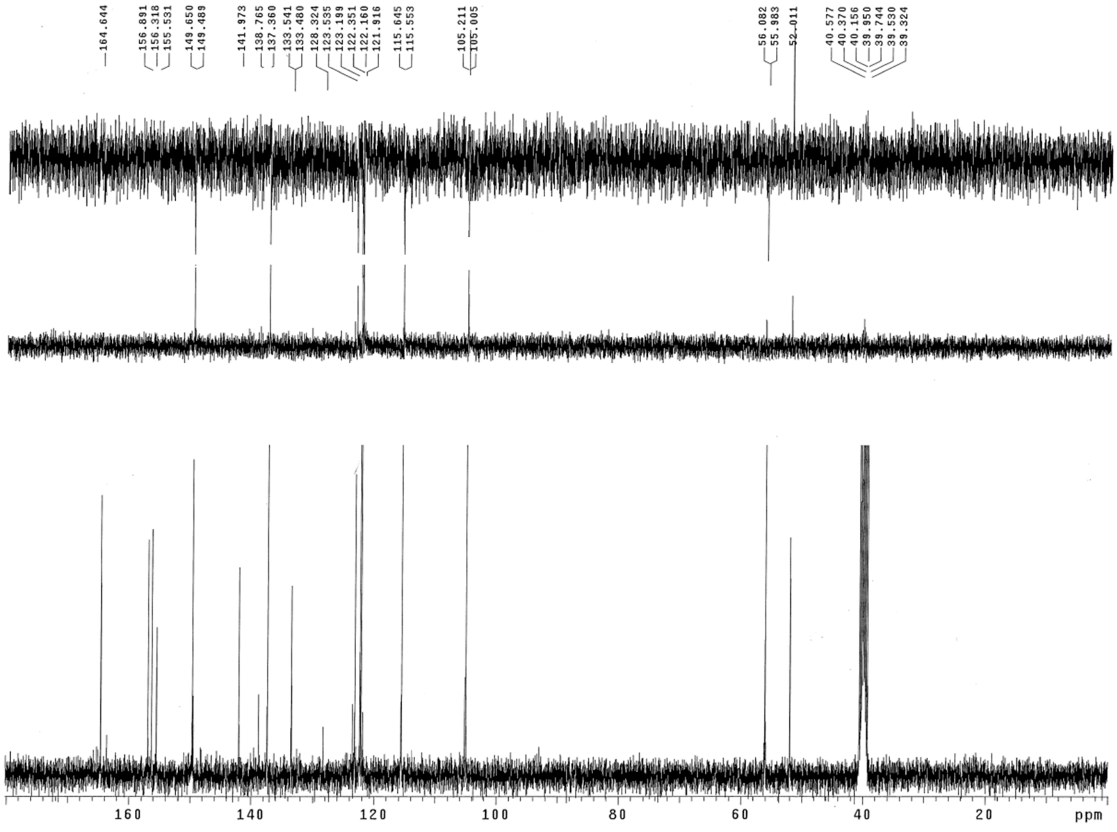

Figure S13b. ^{13}C NMR (DMSO-d_6, 100 MHz) spectra of compound **13**.
